# Analysis of Genes Expression of *Spodoptera exigua* Larvae upon AcMNPV Infection

**DOI:** 10.1371/journal.pone.0042462

**Published:** 2012-07-31

**Authors:** Jae Young Choi, Jong Yul Roh, Yong Wang, Zou Zhen, Xue Ying Tao, Joo Hyun Lee, Qin Liu, Jae Su Kim, Sang Woon Shin, Yeon Ho Je

**Affiliations:** 1 Department of Agricultural Biotechnology, College of Agriculture and Life Science, Seoul National University, Seoul, Korea; 2 Division of Medical Entomology, Korea National Institute of Health, Chungbuk, Korea; 3 School of Life Science, Lanzhou University, Lanzhou, China; 4 State Key Laboratory of Integrated Management of Pest Insects and Rodents, Institute of Zoology, Chinese Academy of Sciences, Beijing, China; 5 Department of Agricultural Biology, College of Agricultural Life Science, Chonbuk National University, Jeonju, Korea; 6 Department of Entomology and Institute for Integrative Genome Biology, University of California Riverside, Riverside, California, United States of America; 7 Research Institute for Agriculture and Life Sciences, Seoul National University, Seoul, Korea; U. Kentucky, United States of America

## Abstract

**Background:**

The impact of *Autographa californica* multicapsid nucleopolyhedrovirus (AcMNPV) infection on host gene expression in *Spodoptera exigua* 4th instar larvae was investigated through the use of 454 sequencing-based RNA-seq of cDNA libraries developed from insects challenged with active AcMNPV or heat-inactivated AcMNPV.

**Methodology/Principal Findings:**

By comparing the two cDNA libraries, we show that 201 host genes are significantly up-regulated and 234 genes are significantly down-regulated by active AcMNPV infection. Down-regulated host genes included genes encoding antimicrobial peptides, namely three gloverin isoforms and an attacin, indicating that the viral infection actively repressed the expression of a portion of the host immune gene repertoire. Another interesting group of down-regulated host genes included genes encoding two juvenile hormone binding proteins and a hexamerin, all of which are involved in juvenile hormone regulation. The expression of these genes was enhanced by the topical application of Juvenile Hormone III (JHIII) in the insects challenged with heat-inactivated AcMNPV. However, infection with the active virus strongly suppresses the expression of these three genes, regardless of the absence or presence of JHIII.

**Conclusions/Significance:**

Using RNA-seq, we have identified groups of immune-regulated and juvenile hormone-regulated genes that are suppressed by infection with active AcMNPV. This information and further studies on the regulation of host gene expression by AcMNPV will provide the tools needed to enhance the utility of the virus as an effective protein expression system and as an insecticide.

## Introduction

Baculoviruses are large DNA viruses that primarily infect insects. Most baculoviruses are quite host-specific, infecting only a single species or a few closely related species, except for *Autographa californica* multicapsid nucleopolyhedrovirus (AcMNPV), which can infect a wide range of lepidopteran insects [1]. The defining features of baculoviruses include circular and supercoiled double-stranded DNA genomes, rod-shaped enveloped nucleocapsids, the production of occluded virions, and the encoding of their own RNA polymerase, and they are obligate parasites of arthropod hosts. Baculovirus gene expression during the viral replication cycle is mediated by two types of RNA polymerases: host RNA polymerase II for the transcription of early and delayed early virus genes and a virus-encoded RNA polymerase for the transcription of late and very late genes. The viral genome sizes vary from approximately 80 to over 180 kb, and they encode between 90 and 180 genes. In general, the virions exist in two different morphological forms: occluded derived virus (ODV) and budded virus (BV). BV spreads the virus from cell to cell in infected insects, whereas ODV spreads the virus between insect hosts [2].

Baculoviruses have long been used for two major purposes: as viral insecticides to control insect pests in agriculture and forestry, and as the basis of a popular eukaryotic protein expression system [3]. They are natural pathogens of insects and have been used to control insect pests such as the codling moth (*Cydia Pomonella*) [4], the velvet bean caterpillar (*Anticarsia gemmatalis*) [5], and the cotton bollworm (*Helicoverpa armigera*) [6]. The high level of very late viral gene expression makes baculoviruses highly suitable as vectors for eukaryotic gene expression. Proteins expressed by the baculovirus expression vector system under the control of the polyhedrin gene (polh) promoter (one of the very late genes), can reach levels of up to 50% of the total cellular protein under optimal conditions. Baculovirus expression, in combination with insect cells or larvae, also results in appropriate posttranslational modifications, in contrast to proteins produced from prokaryotic expression systems. Foreign proteins expressed by baculovirus have been used in a number of vaccines, such as the animal vaccines directed against classical swine fever, or hog cholera [7], [8], and human vaccines against cervical cancer [9] and prostate cancer [10].

The remaining challenges for baculovirus expression systems include the need to improve protein quality by combining various post-translational modifications (such as folding, glycosylation, and preventing degradation) and the need to stabilise the viral genome and the expression of the heterogeneous genes over longer periods of times. In addition, the slow speed of host killing by baculovirus, host adaptations and the complexity of producing standardised viral preparations limit the usage of this virus for insect control. Understanding how baculoviruses interact with their host cells at a molecular level will make it possible to engineer these viruses in a way that will enhance their usefulness as effective insecticides and protein expression systems.

Many viral proteins have been reported or predicted to be involved in host-virus interactions, resulting in host morphological changes after viral infection, inhibition of host apoptosis or molting, regulation of host stress, and host disintegration. During the viral replication cycle, an electron-dense, chromatin-like structure, the virogenic stroma, can be found near the centre of the nuclei of infected cells [11], [12]. In the case of AcMNPV, this host cell morphological change is attributed to two viral proteins: the single-stranded DNA binding protein dbp (Ac25) and PP31 (Ac36) [13], [14]. These two proteins are predicted to be a superoxide dismutase (Ac31, vSOD) and a flavin adenine dinucleotide (FAD)-linked sulfhydryl oxidase (Ac92, p33), based on an HHpred program-based protein homology comparison, and they have been implicated in protection from oxidative stress. Inhibition of host cell apoptosis or host molting is thought to prolong the infection stage, thereby allowing the virus to replicate over a longer period. AcMNPV encodes two copies of a member of the inhibitor of apoptosis (iap) gene family, iap-1 (Ac27) and iap-2 (Ac71). P35 (Ac135) is also an inhibitor of apoptosis and is able to block AcMNPV-induced apoptosis in *S. frugiperda* cells [15]. A viral ubiquitin encoded by AcMNPV Ac35 may regulate host apoptosis to stabilise a short-lived viral protein [16]. When insects were infected with a virus that did not express viral chitinase (Ac126) or cathepsin (Ac127), they remained intact for several days after death, indicating that these viral proteins play a role in the dissemination of the virus by degrading the insect upon death [17].

Baculovirus infection is also reported to affect the expression of host genes. *Bombyx mori* NPV (BmNPV) infection triggered a global down-regulation of host gene expression in insect cells beginning at approximately 12–18 h post infection (hpi) [18]. Down-regulation of host mRNAs following AcMNPV infection in *Spodoptera frugiperda* (Sf9) cells has been reported in multiple studies [19], [20], [21]. A global analysis using a differential display method found that AcMNPV infection in Sf9 cells caused global down-regulation of host mRNA levels at later time points during the infection (12–24 hpi), but up-regulated the heat shock protein cognate 70 (hsc70) at earlier points [22]. A comprehensive microarray analysis followed by qRT-PCR analysis identified the up-regulation of several host genes, including hsp70 [23].

To identify the effect of AcMNPV infection on the expression of host transcripts in *Spodoptera exigua* larvae, we used 454 sequencing to analyse the transcriptome. This is a newer alternative to traditional EST sequencing and is a much more cost effective means of sequencing transcriptomes. In addition, this method allows *de novo* sequencing, assembly and annotation of expressed genes in a non-model organism for which genome sequences are currently unavailable. The 454 sequencing technique can also be used to investigate transcriptome-wide differential gene expression between differently treated samples. For this study, we sequenced the cDNA libraries from insects treated with active AcMNPV and heat-inactivated AcMNPV 12 h after treatment. The combined read sequences from the two transcriptomes were then used to construct a pool of contigs. The read numbers from the two transcriptomes were compared to identify host genes that were up- or down-regulated after viral infection. Out of 5,945 total contigs, 201 genes are significantly up-regulated and 234 genes significantly down-regulated by active AcMNPV infection, as compared to heat-inactivated AcMNPV infection. Two small groups of host genes, a group of genes encoding antimicrobial peptides/proteins (AMPs) (gloverins and an attacin) and a group of three juvenile hormone-related genes, were down-regulated. The genes encoding the AMPs were strongly induced by challenge with the heat-inactivated AcMNPV, but this induction was suppressed by active AcMNPV. The genes encoding the two juvenile hormone binding proteins and a hexamerin were induced by challenge with the heat-inactivated AcMNPV and additionally enhanced by the application of Juvenile Hormone III (JHIII). This up-regulation was not observed in insects infected with active AcMNPV. These results strongly suggest that the active virus can suppress the expression of specific host genes.

## Results and Discussion

### 454 sequencing results and contig assembly

As described in the Methods, cDNA libraries from *Spodoptera exigua* larvae challenged with active AcMNPV or heat-inactivated AcMNPV were subjected to a 1/8-plate production run on the 454 GS-FLX sequencing instrument, resulting in 77,616 and 74,928 reads, respectively. Files containing these data were deposited in the Short Read Archive of the National Center for Biotechnology Information (NCBI) with accession numbers of SRX110132 (AcMNPV-challenged) and SRX110248 (heat-inactivated AcMNPV-challenged), respectively. After the sequence reads of viral origin were removed, a total of 130,335 total reads were obtained from the two cDNA libraries, which were assembled to create 5,945 contigs (>100 bp, average length of 667 bp) (Table S1). A total of 3,607 contigs were at least 500 bp in length, a greater number than that obtained by 454 pyrosequencing in other insects such as *Anopheles funestus* [24], *Meliteae cinxia* [25] and *Zygaena filipendulae* [26]. The average number of reads assembled into a contig was 21.9. All files of assembled contigs and singletons from AcMNPV-challenged, heat-inactivated AcMNPV-challenged, and combined EST libraries are available by request.

**Figure 1:**
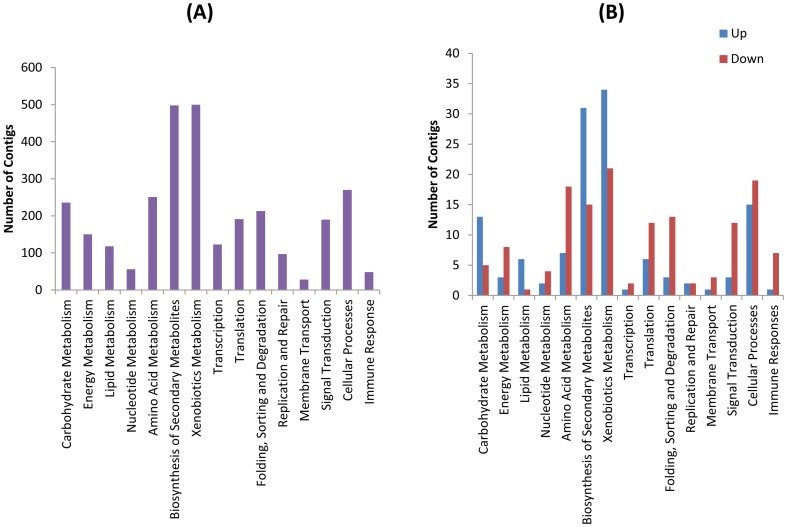
KEGG analysis of total (A), UP and DOWN (B) contigs. (A) Functional groups defined by KEGG were classified as Carbohydrate metabolism, Lipid metabolism, Nucleotide metabolism, Amino acid metabolism, Biosynthesis of other secondary metabolites, Xenobiotics biodegradation and metabolism, Transcription, Translation, Folding Sorting and Degradation, Replication and Repair, Membrane Transport, Signal Transduction, and Cellular Processes. The immune response category was directly defined by comparing to *Drosophila* immune genes using BLASTN. (B) The contigs that showed significantly different expression (a binominal probability of <0.1) were classified as UP or DOWN, where UP indicates genes that are up-regulated by active AcMNPV infection compared to heat-inactivated AcMNPV challenge and DOWN indicates genes that are down-regulated by active AcMNPV infection compared to heat-inactivated AcMNPV challenge.

To obtain an overview of the functional categories represented by the *S. exigua* transcriptome, we compared the 5,945 contigs with a *Drosophila* database using BLASTX. A *Drosophila*-based gene ontology search categorised 2,699 hits into 15 functional groups (Figure 1A). Enzymes involved in the metabolism of secondary metabolites and xenobiotics represented the two largest groups, accounting for 30% of the total number of contigs with a putative function. Metabolism of carbohydrates (236), energy (150), amino acids (251), lipids (118), and nucleotides (56) accounted for another 30%. While genes related to metabolism represented the largest collection of contigs overall, the genes involved in transcription (123) and translation (191), which constituted the basic genetic information processing machinery, were also highly expressed. Relatively low abundance genes that were identified by this analysis included genes encoding proteins involved in membrane transport and other cellular processes, including genes related to cell mobility, growth, death, and communication. The apparent low abundance of these transcripts could be due to the low homology to *Drosophila* proteins or to an inherent bias in the library construction.

### Comparison of transcriptome profiles between active AcMNPV-infected and heat-inactivated AcMNPV-treated samples

The significant (binominal probability of <0.1) differences in the expression of different contigs were determined by comparing the read number of each contig between the active AcMNPV-infected sample (A-read) and the heat-inactivated AcMNPV-treated sample (I-read). Using this method, we identified 201 host genes that are significantly up-regulated and 234 genes that are significantly down-regulated by infection with active AcMNPV (Table S2 & S3). The distribution of gene functions between these two groups is quite distinctive (Figure 1B). Genes related to carbohydrate and lipid metabolism were concentrated in the up-regulated gene (UP) cohort. Likewise, twice as many genes related to secondary metabolism and xenobiotic metabolism were present in the UP group as compared to the group of down-regulated genes (DOWN). The UP group includes eight genes encoding cytochrome P450 family proteins, and one gene each encoding glutathione S-transferase, thioredoxin peroxidase, superoxide dismutase, and methylenetetrahydrofolate dehydrogenase, which are key enzymes in the detoxification of xenobiotic compounds. However, genes related to amino acid and nucleotide metabolism, such as alanine aminotransferase, amidophosphoribosyltransferase, and glutamine synthetase, were more suppressed in the presence of the viruses. More genes involved in translation, degradation, and signal transduction were also down-regulated in the live virus-treated samples as compared to the heat-inactivated samples. Eight genes related to the host immune response are found in the suppressed gene repertoire, including the gene encoding attacin and three genes encoding gloverin, all of which are immune effector molecules.

### Viral gene expression 12 h post infection

We characterised the sequences of viral origin from both the A-read and the I-read by performing a BLASTN homology search to AcMNPV open reading frame (ORF) sequences. The A-read from the active AcMNPV challenged-cDNA library included 614 read sequences that originated from AcMNPV, whereas the I-read from the heat-inactivated AcMNPV sample only produced one sequence (Table S4). Out of 155 ORFs, the expression of 93 AcMNPV ORFs was detected by RNA-seq in host insect larvae at 12 hpi (Table S5).

To obtain an overview of AcMNPV gene expression 12 hpi, we grouped the AcMNPV ORFs based on their known functions and on their abundance in the A-read (Table 1). Here, we found that 5 of 6 ORFs encoding viral *per os* infectivity factors, which are involved in the initiation of midgut infections [27], were not detected (i.e., belonged to the no frequency group). This was expected because the virus was directly injected into the insect hemocoel cavity. We also found that 22 of 25 ORFs encoding viral structural proteins belonged primarily to the low frequency (# of A-reads is less than 10) or no frequency (# of A-reads is zero) groups. In addition, two of three ORFs encoding the viral RNA polymerase belonged to the no frequency group. Both viral structural proteins and the viral RNA polymerase are involved in the late or very late stages of the viral replication cycle. Furthermore, we did not detect any expression of the two ORFs encoding a chitinase and a cathepsin, which are involved in insect disintegration. In contrast, 10 of 11 ORFs encoding viral proteins involved in DNA replication belonged to the high frequency or low frequency (# of A-reads is 10 or greater than 10) groups. These results indicate that, at 12 hpi, the viruses are primarily in the early stages of the viral replication cycle.

**Table 1:** An overview of AcMNPV gene expression 12 hpi.

	High frequency (# of A-reads > = 10)	Low frequency (# of A-reads <10)	No frequency
Structural proteins			
Occlusion bodies		Ac8 (polyhedrin), Ac131 (polyhedrin envelope), Ac137 (p10)	
Baculovirus envelope proteins	Ac128 (Gp64)	Ac16 (BV/ODV-E26), Ac23 (Fusion protein-F), Ac94 (ODV-E25), Ac143 (ODV-E18)	Ac46 (ODV-E66), Ac109 (ODV-EC43), Ac148 (ODV-E56)*
Nucleocapsid associated factors	Ac100 (P6.9), Ac144 (ODV-EC27)	Ac9 (PP78/83), Ac80 (GP41), Ac89 (major capsid protein), Ac101 (BV/ODV-C42), Ac104 (VP80), Ac142 (p49)	Ac54 (vp1054), Ac66, Ac77 (VLF-1), Ac92 (P33), Ac98 (38K), Ac129 (P24), Ac141
Viral transcription activators	Ac147 (IE1)	Ac151 (ie2/ie-n), Ac153 (pe38)	
DNA replication (essential)	Ac67 (LEF-3), Ac147 (IE1)	Ac6 (LEF-2), Ac14 (LEF-1), Ac65 (DNA pol), Ac95 (p143)	
DNA replication (influential)	Ac25 (DBP), Ac139 (ME53)	Ac49 (PCNA), Ac125 (LEF-7)	Ac37 (LEF-11)
Viral RNA polymerase		Ac50 (LEF-8)	Ac40 (p47), Ac62 (LEF-9)
*per os* infectivity factors		Ac119 (pif-1)	Ac22 (pif-2), Ac96 (pif-4), Ac115 (pif-3), Ac 138 (P74-pif), Ac148 (pif-5)*
Proteins involved in host-virus interaction			
Oxidative stress			Ac31 (vSOD), Ac92 (p33)
Prolonged infection (Inhibition of apoptosis or molting)	Ac15 (EGT), Ac135 (p35)		Ac27 (iap-1), Ac71 (iap-2), Ac35 (viral ubiquitin)
Insect disintegration			Ac126 (chitinase), Ac127 (cathepsin)
Actin assembly		Ac9 (pp78/83), Ac20/21 (arif-1)	
Virogenic stroma	Ac25 (dbp), Ac36 (pp31)		

* indicates that the viral protein is a baculovirus envelope protein, which has role in *per os* infectivity [46].

BLASTN analysis using the viral ORF sequences showed that 614 read sequences from the active AcMNPV challenged-cDNA library (A-read) originated from the virus itself. The viral ORFs are grouped based on their known functions and the number of A-reads.

### A global down-regulation of host mRNA levels was not observed

**Figure 2:**
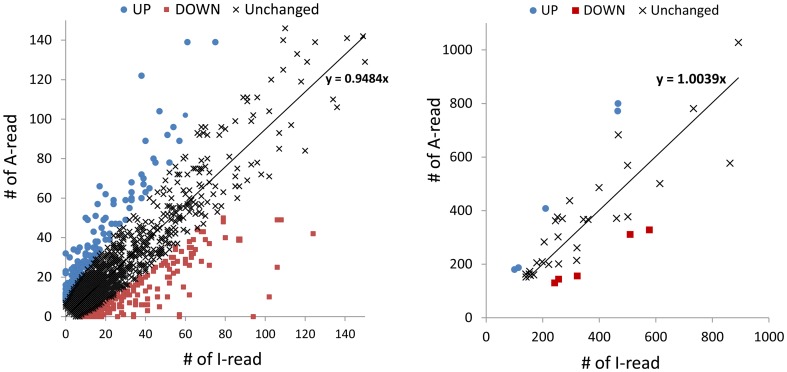
Graph of the numbers of I-reads and A-reads for each contig. The number of I-reads and A-reads of each contig were graphed on an x,y plot. For convenience, contigs were plotted on two separate graphs: for contigs shown in the left panel, the number of I- or A-reads is smaller than 150; for contigs shown in the right panel, the number of I or A-reads is equal to or larger than 150. The linear trendline (with the intercept set as zero) and the slope are indicated by a line and an equation. UP and DOWN contigs are indicated as green circles and red boxes, respectively.

**Figure 3:**
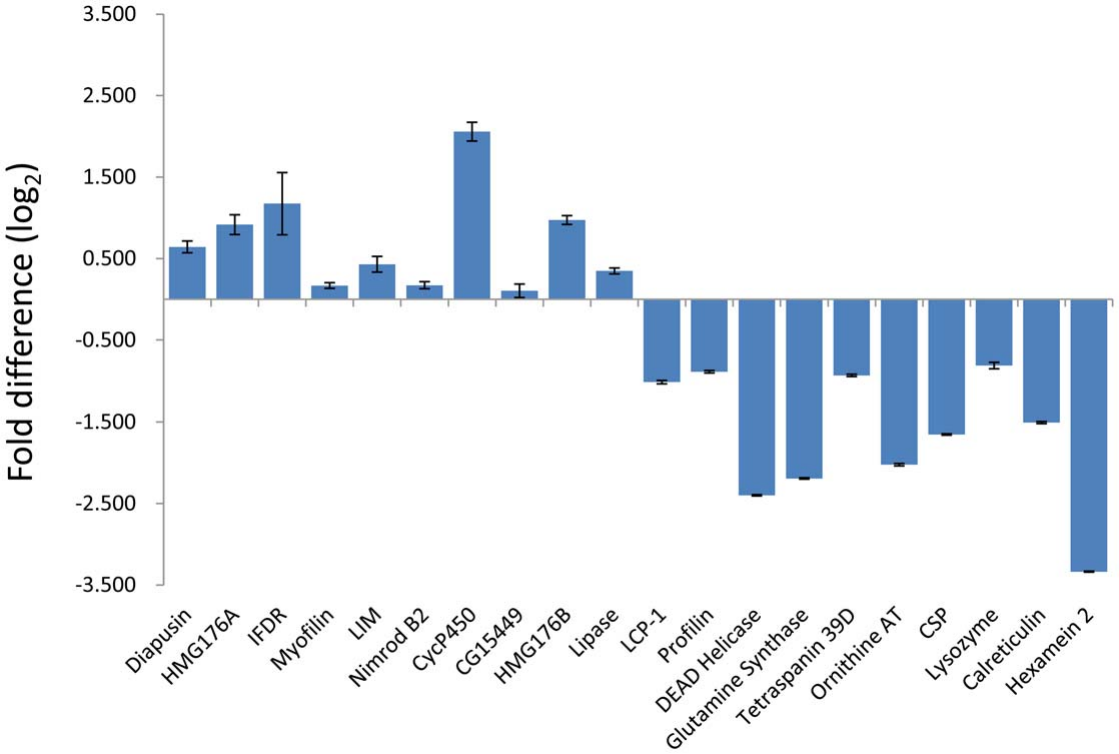
Validation of the RNA-seq results by quantitative real-time PCR (qPCR). The expression profiles of 10 UP and 10 DOWN contigs (randomly selected) were analysed by qPCR to validate the RNA-seq results. The tested contigs are indicated in red in Tables S2 and S3.

The number of I-reads and A-reads for each contig were graphed on an x,y plot, showing that the expression of the majority of host genes is not significantly affected (p>0.1) (Figure 2). Only approximately 7.3% of contigs were up- (UP) or down- (DOWN) regulated by active AcMNPV infection. To confirm the expression profiles of the UP and DOWN gene categories, total RNA was isolated from 5th instar larvae 12 hpi with active or heat-inactivated AcMNPV. When 10 UP and 10 DOWN genes were tested by quantitative real-time PCR (qPCR), their expression profiles all matched the results obtained by RNA-seq (Figure 3, indicated in red in Table S2 and S3).

**Figure 4:**
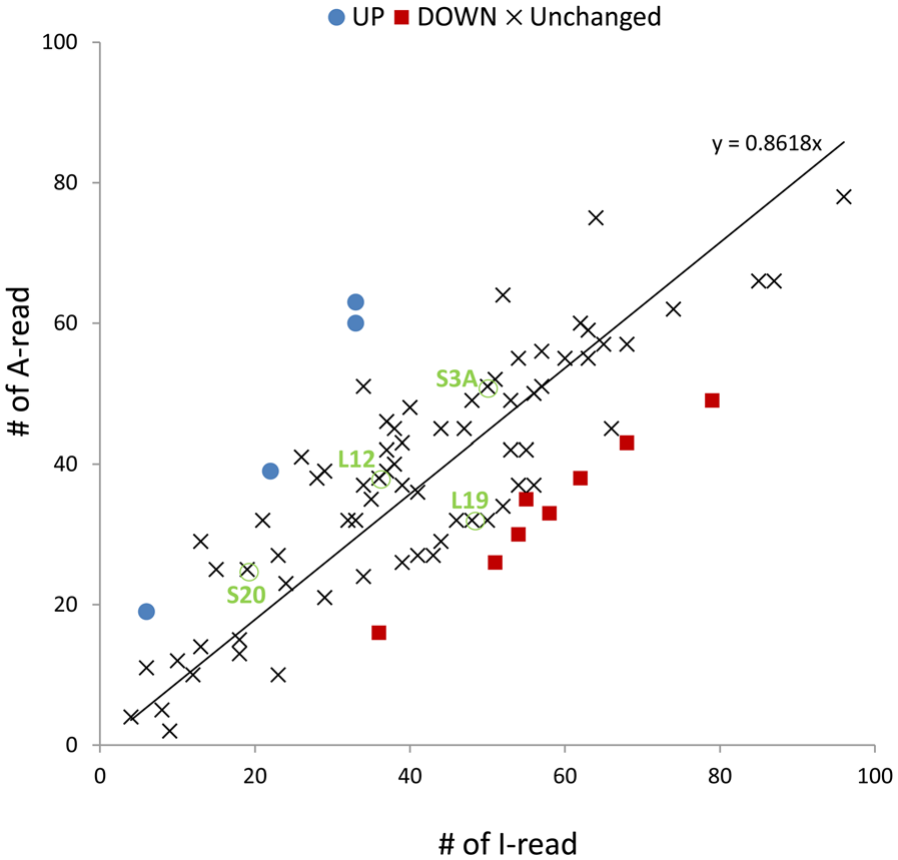
Graph of the number of reads from contigs encoding ribosomal proteins. The expression of four ribosomal protein genes, RpS20, RpSL12, RpL19 and RpS3A (indicated by green circles), which have been cited as examples of global down-regulation of host transcription in Sf9 cells [45], was not significantly altered in our experiments, clearly indicating that there is no global down-regulation of host transcripts.

A global down-regulation of host transcription at late time points of infection has been reported in several studies [19], [20], [21]. A differential display approach showed that the decrease in host mRNA levels began between 12 and 24 hpi in Sf9 cells [22]. By means of a microarray approach, transcripts for the majority of host genes in Sf9 cells were shown to decline substantially 12 hpi [23]. In our experimental approach, we infected the insect larvae with AcMNPV, and no global down-regulation of host gene expression was observed. Only a small number of genes were significantly down-regulated by active AcMNPV infection (234 DOWN of 5,945 total contigs). We detected a similar number of contigs in the DOWN and UP groups (234 and 201, respectively). When the number of I-reads and A-reads for the contigs encoding ribosomal proteins were graphed, we observed that the expression of the majority of genes encoding ribosomal proteins are not significantly affected (Figure 4). Out of 84 contigs encoding ribosomal proteins, only 4 host genes are significantly up-regulated and 8 are down-regulated by active AcMNPV infection. The down-regulation of 4 genes encoding ribosomal protein (RpS20, RpSL12, RpL19 and RpS3A) in Sf9 cells at 18 h or 24 h after infection with AcMNPV has been reported as evidence of a global down-regulation in host gene expression [23]. However, the expression of these genes was not significantly altered in our experiments (Figure 4), clearly indicating that there is no global down-regulation of host transcripts.

Although there were previous reports that describe a global down-regulation of the host mRNA by AcMNPV infection, those studies were carried out *in vitro* system using Sf9 cells, which were infected with the virus at an MOI (multiplicities of infection) of 10. Because most cultured cells (over 99%) were infected with the virus simultaneously under these infection conditions, transcripts for the majority of host genes in Sf9 cells were shown to decline substantially between 12–24 hpi. In contrast, in this study, *in vivo S. exigua* larvae were used as host system, in which only several parts of tissues including hemocyte and fatbody could be actually infected with injected viruses and most of tissues were remained as non-infected at 12 hpi, at which time point total RNA was isolated from whole body of the infected larvae. This was further supported by the viral gene expression at 12 hpi (Table 1) suggesting that, in *S. exigua* larvae, the viruses are primarily in the early stages of the viral replication cycle at 12 hpi. More prolonged infection times are needed to observe global host gene down-regulation in *S. exigua* larvae.

### Suppression of host immune gene activation by AcMNPV

**Figure 5:**
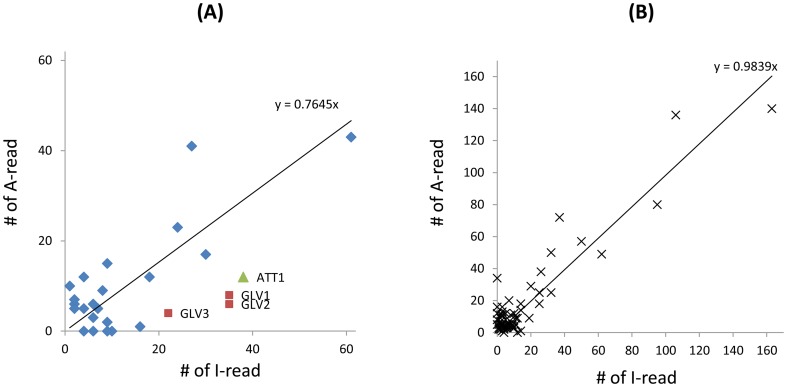
Graph of the number of reads from contigs encoding host immune-related peptides/proteins (A) and serine proteases (B). Three gloverin genes and one attacin gene are indicated by red boxes and a green triangle, respectively. The linear trendline (with the intercept set at zero) and the slope are indicated by a line and an equation.

When the number of I-reads and A-reads for the contigs encoding immune-related peptides/proteins were graphed, we observed that the expression of several genes involved in the host immune response was significantly down-regulated in larvae infected with active AcMNPV (Figure 5A). These down-regulated genes include genes encoding three gloverins and one attacin. Gloverin is a glycine-rich antibacterial protein found in lepidoptera species [28], [29]. One recent report showed that *S. exigua* gloverin (GLV) acts as an antimicrobial peptide (AMP) against *Bacillus thuringiensis* [30]. Attacin is also a glycine-rich protein, originally isolated from the lepidopteran insect *Hyalophora cecropia* [31]. The expression of attacin cDNA has been reported in *S. exigua* [32].

**Figure 6:**
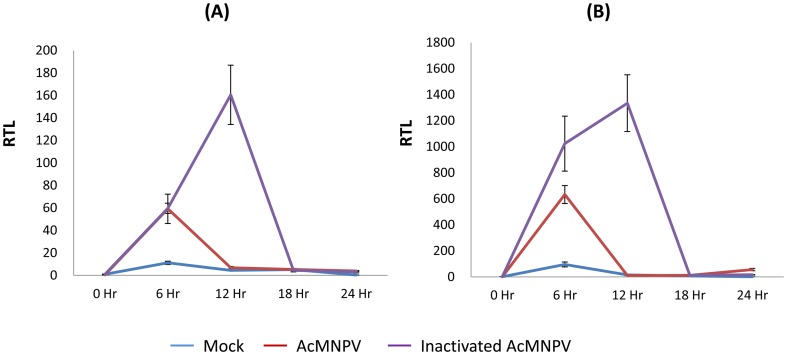
The expression profiles of the GLV1 (A) and ATT1 (B) genes. The relative expression levels of the GLV1 and ATT1 genes 6 h after each treatment were examined by qPCR. Mock, treatment with Sf9 growth medium; AcMNPV, culture medium with AcMNPV; Inactivated AcMNPV, culture medium with heat-inactivated AcMNPV.

We analysed the expression profiles of GLV1 and attacin by qPCR. Gene expression was slightly up-regulated at 6 hpi in the control insects, which were mock-injected with Sf9 growth medium. We observed 5–7 times greater expression of both AMP genes 6 hpi in the insects infected with active or heat-inactivated AcMNPV (Figure 6), as compared to mock injection, suggesting that AcMNPV infection elicits some type of immune response. Interestingly, the GLV1 and attacin expression decreased rapidly at 12 hpi with active AcMNPV, whereas these genes continued to be up-regulated until 12 hpi in the insects infected with heat-inactivated AcMNPV (Figure 6). This clearly indicates that the active virus can suppress the induction of these AMP genes. It has been reported that AMP genes are expressed as an acute immune response to bacterial challenge, and therefore are rapidly transcribed following challenge (1 to 5 h), the transcription rate increases over a period varying between 6 and 24 h, depending on the gene, and thereafter either stops or levels off [33]. Therefore, it could be postulated that these immune related genes were induced in *S. exigua* larvae upon hemocoelic injection of AcMNPV (regardless of active and heat-inactivated), but the further expression of them was suppressed by viral modulation of host immune mechanisms in *S. exigua* larvae injected with the active AcMNPV at 12 hpi. This result may also suggest a possible antiviral role for these glycine-rich AMPs, a hypothesis that will be addressed by further research.

### AcMNPV infection affects the hormonal regulation of host gene expression

The group of DOWN contigs included three genes encoding proteins that belong to the juvenile hormone binding protein (JHBP) family and three genes encoding hexamerin proteins (Table S3). Low molecular weight JHBPs of approximately 30 kDa have specific affinity for the juvenile hormone (JH) [34]. Insect hexamerins have been shown to be *bona fide* JH-binding proteins [35], [36] and reportedly bind to JHBP [37]. Both JHBP and hexamerin are proposed hemolymph carriers, which are involved in protecting JH from hydrolysis by esterases during transport from its site of synthesis to target tissues.

**Figure 7:**
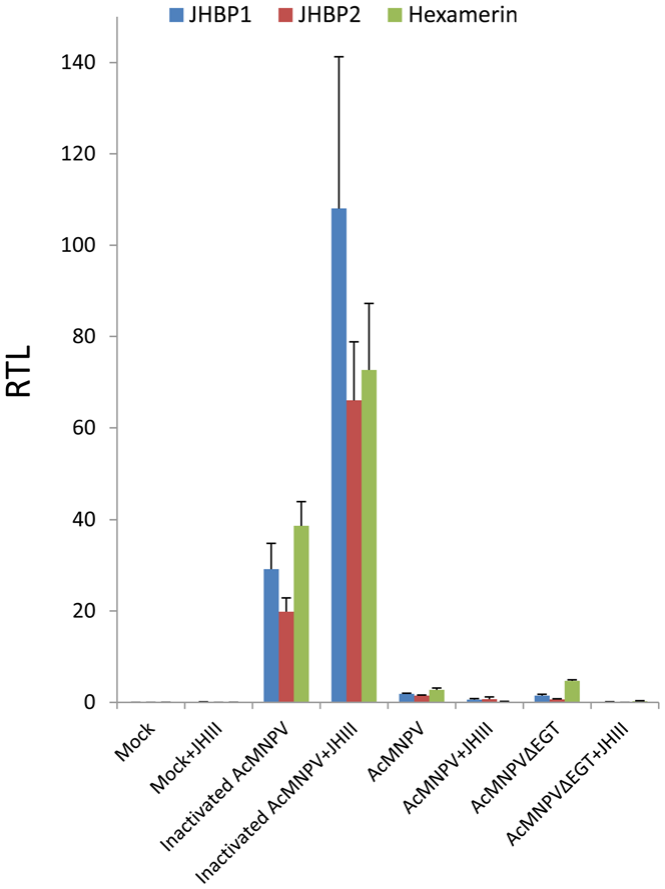
The expression profiles of two JHBP genes and one hexamerin gene. Relative expression levels of two JHBP genes and one hexamerin gene 12 h after each treatment were examined by qPCR. Mock, treatment with Sf9 growth medium; AcMNPV, culture medium with AcMNPV; Inactivated AcMNPV, culture medium with heat-inactivated AcMNPV; AcMNPVΔEGT, culture medium with AcMNPVΔEGT. JHIII was topically applied directly after each treatment.

We analysed the expression profile of two JHBP genes (JHBP1 and JHBP2) and one hexamerin gene (contig00429) by qPCR. The expression was strongly induced at 12 hpi with heat-inactivated AcMNPV (Figure 7). This induction was further enhanced by JHIII treatment (Figure 7), demonstrating that JH was involved in the regulation of these JH carrier genes. Interestingly, up-regulation of these genes was not observed 12 hpi with live AcMNPV, suggesting that the active viruses inhibit JH-related regulation of host genes.

We propose that JH may be involved in the host defence against baculovirus infection by interfering in the viral life cycle. In order for successful viral replication to occur, the baculovirus needs to control host molting and pupation in infected larvae. Host metamorphosis is regulated by two hormones: ecdysone, which causes molting, and JH, which allows larval molting but prevents pupation. Host insects may respond to AcMNPV infection by increasing JH trafficking, which could explain the activation of JH carrier genes by heat-inactivated AcMNPV. Live AcMNPV counteracts this measure by suppressing gene activation, thereby controlling host molting and pupation to be more favourable for the viral replication cycle.

Host ecdysone levels have been reported to be controlled by viral ecdysteroid UDP-glucosyltransferase (EGT). Viral EGT functions to block molting and pupation in infected insect larvae by inactivating ecdysone hormone [38]. This is thought to cause abnormal larval growth and prolongation of the larval instar, resulting in a higher virus yield [39]. Because a reciprocal interaction between JH and ecdysone in gene regulation has been reported [40], it is also possible that baculovirus infection affects the host ecdysone level, consequently triggering the activation of JH-dependent genes encoding the JHBPs and hexamerin. To test this hypothesis, we surveyed the gene expression profiles after infection with an AcMNPV EGT deletion mutant (AcMNPVΔEGT). This mutant was still able to suppress the gene activation, clearly indicating that the suppression of JH-related genes was not mediated through host ecdysone levels induced by viral EGT.

In this paper, we describe the suppression of host JH-related genes by AcMNPV. We hypothesise that the host insect increases the level of available juvenile hormone after the viral infection, but that active AcMNPV counteracts this measure through an unknown mechanism that does not involve viral EGT regulation of host ecdysone levels.

## Materials and Methods

### Insect cells, insects and viruses

The *S. frugiperda* cell line, Sf9, was maintained in TC-100 medium (WelGene, Korea) supplemented with 10% heat-inactivated (56°C, 30 min) fetal bovine serum (WelGene, Korea) at 27°C. The *S. exigua* larvae were obtained from a laboratory colony and raised at 25°C under a 16 h∶8 h light∶dark cycle with an artificial diet [41]. The wild-type AcMNPV C6 strain was propagated in Sf9 cells maintained in TC-100 medium. The AcMNPV virus was heat-inactivated by incubating at 60°C for 24 h.

### Viral infection, JHIII treatment and RNA extraction

Inoculation of the insect larvae was carried out as described previously [42]. Approximately 50 µl of a viral suspension (1×10^7^ PFU/ml) (containing active or heat-inactivated virus) with 6 mg/ml kanamycin was injected just underneath the dorsal cuticle of each *S. exigua* 5th instar larva using a Hamilton digital syringe (Hamilton, USA) fitted with a 22s-gauge needle. JHIII (Sigma, USA) was applied topically (10 mg/ml in acetone, 1 µl/larva) to *S. exigua* larvae. After incubation at 25°C for 12 h, total RNA was isolated from infected *S. exigua* larvae using TRIZOL Reagent (Invitrogen, USA) according to the manufacturer's instructions. The extracted total RNA was first treated with DNaseI (TaKaRa, Japan) to remove contaminating genomic DNA.

### 454 sequencing and sequence analysis

From the total RNA extracted from whole body of *S. exigua* 5th instar larvae, mRNAs were purified using the Oligotex mRNA Midi Kit (QIAGEN, Germany). Next, double-stranded cDNA was synthesised using the SMART cDNA Library Construction Kit (Clontech, USA). The cDNA was further purified using the QIAquick PCR Purification Kit (QIAGEN, Germany) and checked for quality using an Agilent 2100 Bioanalyzer. Approximately 5 µg of cDNA was used for sequencing on a Roche/454 GS-FLX Titanium sequencer (Roche, Germany). A 1/8 plate sequencing run was performed at MACROGEN Co. (Korea) according to the manufacturer's instructions. The raw 454 sequence files were processed to remove low quality regions and adaptor sequences using the SeqClean program (http://compbio.dfci.harvard.edu/tgi/software). The resulting sequences were then screened against the NCBI UniVec database and *E. coli* genome sequences to remove contaminating sequences. Sequences shorter than 100 bp were discarded. The processed sequences were assembled into contigs using the iAssembler program (http://bioinfo.bti.cornell.edu/tool/iAssembler).

### Analysis of relative transcription levels by qPCR

The 28S rRNA gene was used as a reference gene [43]. Single-strand cDNA was synthesised from the total RNA using the SuperScript™ III First-Synthesis System for RT-PCR (Invitrogen, USA) according to the manufacturer's instructions. Real-time PCR was conducted using the 2× DyNAmo™ HS SYBR® Green qPCR Kit (FINNZYMES, Finland) and a CFX96™ Real-Time System (BIO-RAD, USA). The cycling profile used for qPCR was as follows: a preheating step for enzyme activation at 95°C for 10 min, followed by 40 cycles of 95°C for 15 sec, 58°C for 15 sec and 72°C for 30 sec. The relative transcription levels were calculated using the 2^−ΔCt^ method [44]. The primers used for qPCR are listed in Table S6.

### Identification of functional classes

The contigs were analysed by comparing to *Drosophila* proteins using BLASTX (with a cut-off value of 1e-05). The resulting data set was used to reconstruct functional class profiles and pathways using the KEGG (Kyoto Encyclopedia of Genes and Genomes), except for the Immune response category, which was directly defined by comparing to *Drosophila* immune genes using BLASTN. The annotation results of *S. exigua* contigs derived from BLASTX were subsequently analysed in PROSITE, SMART, PFAM to confirm conserved domain structures.

## Supporting Information

Table S1 **List of assembled contigs with total reads from the cDNA libraries from both the AcMNPV-challenged and the heat-inactivated AcMNPV-challenged** ***S. exigua*** **.**
(XLS)

Table S2 **List of** ***S. exigua*** **genes significantly up-regulated by active AcMNPV infection.**
(DOC)

Table S3 **List of** ***S. exigua*** **genes significantly down-regulated by active AcMNPV infection.**
(DOC)

Table S4 **Number of read sequences originating from AcMNPV.**
(DOC)

Table S5 **Fasta sequence of the 93 AcMNPV unigenes assembled from the RNA-seq.**
(DOC)

Table S6 **Primer sequences used in quantitative real-time PCR analysis.**
(DOC)
