# Supplementary material for: Analysis of Genes Expression of Spodoptera exigua Larvae upon AcMNPV Infection
Source: PLoS One. 2012 Jul 31;7(7):e42462. doi: 10.1371/journal.pone.0042462 (PMC3409162; doi:10.1371/journal.pone.0042462)
Supplement: Table S2 — List of S. exigua genes significantly up-regulated by active AcMNPV infection. (DOC) [file pone.0042462.s002.doc]

Table S2. List of *S. exigua* genes significantly up-regulated by active AcMNP infection.

| Contig # | # T-read | # A-read | # I-read | Fold activation | Binomial probability | Gene prediction |
| --- | --- | --- | --- | --- | --- | --- |
| contig05744 | 1335 | 836 | 499 | 1.68 | 0 | hexamerin |
| contig05780 | 1266 | 800 | 466 | 1.72 | 0 | hexamerin |
| contig03325 | 1237 | 772 | 465 | 1.66 | 0 | hexamerin |
| contig05841 | 618 | 408 | 210 | 1.94 | 3.22629E-16 |  |
| contig03451 | 301 | 187 | 114 | 1.64 | 6.19987E-06 |  |
| contig03491 | 280 | 180 | 100 | 1.80 | 4.60488E-07 |  |
| contig03786 | 214 | 139 | 75 | 1.85 | 3.43543E-06 | desaturase |
| contig05000 | 200 | 139 | 61 | 2.28 | 1.00555E-08 |  |
| contig00730 | 162 | 102 | 60 | 1.70 | 0.000262929 | ornithine decarboxylase antizyme |
| contig05891 | 160 | 122 | 38 | 3.21 | 6.24593E-12 | **diapausin** |
| contig00069 | 151 | 104 | 47 | 2.21 | 1.13469E-06 |  |
| contig05895 | 150 | 96 | 54 | 1.78 | 0.000174866 | superoxide dismutase |
| contig00103 | 146 | 89 | 57 | 1.56 | 0.001968886 |  |
| contig05836 | 143 | 92 | 51 | 1.80 | 0.000179165 |  |
| contig04041 | 130 | 78 | 52 | 1.50 | 0.005201573 | cysteine and glycine-rich protein |
| contig00758 | 129 | 89 | 40 | 2.23 | 5.42678E-06 | **HMG176 [*Helicoverpa armigera*], HMG176A** |
| contig04885 | 124 | 80 | 44 | 1.82 | 0.000372237 | Ferritin 2 light chain homologue |
| contig00145 | 123 | 78 | 45 | 1.73 | 0.000843203 | cytochrome P450 |
| contig03421 | 110 | 72 | 38 | 1.89 | 0.000382034 |  |
| contig03917 | 109 | 70 | 39 | 1.79 | 0.000910469 |  |
| contig03333 | 107 | 65 | 42 | 1.55 | 0.00652314 |  |
| contig00298 | 106 | 67 | 39 | 1.72 | 0.001899229 | protein kinase |
| contig03581 | 103 | 63 | 40 | 1.58 | 0.006036579 | Sorbitol dehydrogenase |
| contig05807 | 101 | 68 | 33 | 2.06 | 0.000172644 |  |
| contig00016 | 98 | 60 | 38 | 1.58 | 0.006834922 | Chitin deacetylase-like |
| contig00509 | 96 | 63 | 33 | 1.91 | 0.000727057 | ubiquitin |
| contig01332 | 93 | 60 | 33 | 1.82 | 0.001616582 | ribosomal protein L31 [Spodoptera frugiperda] |
| contig05708 | 92 | 59 | 33 | 1.79 | 0.002085913 | pugilist |
| contig00183 | 89 | 59 | 30 | 1.97 | 0.000725003 |  |
| contig00886 | 87 | 55 | 32 | 1.72 | 0.004077167 |  |
| contig00274 | 83 | 66 | 17 | 3.88 | 2.10704E-08 | **interferon-related developmental regulator 2, IFDR** |
| contig00148 | 83 | 59 | 24 | 2.46 | 4.74117E-05 | **myofilin** |
| contig03458 | 82 | 62 | 20 | 3.10 | 1.28394E-06 |  |
| contig03440 | 81 | 57 | 24 | 2.38 | 9.53529E-05 | HMG176 [*Helicoverpa armigera*] |
| contig06172 | 79 | 49 | 30 | 1.63 | 0.009172826 |  |

Table S2. Continued.

| Contig # | # T-read | # A-read | # I-read | Fold activation | Binomial probability | Gene prediction |
| --- | --- | --- | --- | --- | --- | --- |
| contig05998 | 74 | 47 | 27 | 1.74 | 0.006218426 |  |
| contig02069 | 73 | 57 | 16 | 3.56 | 5.58169E-07 | **muscle LIM protein, LIM** |
| contig04799 | 72 | 47 | 25 | 1.88 | 0.003232384 | glucose transmembrane transporter |
| contig04127 | 70 | 50 | 20 | 2.50 | 0.000137122 | asteroid |
| contig03515 | 70 | 47 | 23 | 2.04 | 0.001517551 | **nimrod B2** |
| contig04808 | 68 | 49 | 19 | 2.58 | 0.000113558 |  |
| contig00461 | 66 | 51 | 15 | 3.40 | 3.63705E-06 | **cytochrome P450** |
| contig01000 | 64 | 42 | 22 | 1.91 | 0.004355644 | Trehalose-6-phosphate synthase |
| contig05816 | 63 | 45 | 18 | 2.50 | 0.000280669 |  |
| contig04171 | 61 | 42 | 19 | 2.21 | 0.001287959 | **CG15449 [*Drosophila melanogaster*]** |
| contig04218 | 61 | 39 | 22 | 1.77 | 0.009601151 | ribosomal protein L30 |
| contig04148 | 60 | 50 | 10 | 5.00 | 6.53939E-08 | aldehyde dehydrogenase 1 family, member L1 |
| contig05850 | 60 | 49 | 11 | 4.45 | 2.97245E-07 | **HMG176 [*Helicoverpa armigera*], HMG176B** |
| contig02305 | 59 | 39 | 20 | 1.95 | 0.004847794 | CG6910 [Drosophila melanogaster] |
| contig03956 | 57 | 43 | 14 | 3.07 | 5.33923E-05 |  |
| contig03390 | 54 | 38 | 16 | 2.38 | 0.001171003 | repeptin |
| contig05612 | 53 | 38 | 15 | 2.53 | 0.000693928 |  |
| contig04530 | 51 | 34 | 17 | 2.00 | 0.006559672 | C-type lectin |
| contig01523 | 50 | 35 | 15 | 2.33 | 0.001999138 |  |
| contig01452 | 49 | 35 | 14 | 2.50 | 0.001199483 | cytochrome P450 |
| contig06302 | 49 | 34 | 15 | 2.27 | 0.002798794 |  |
| contig02958 | 49 | 33 | 16 | 2.06 | 0.005947436 | eukaryotic translation initiation factor |
| contig02268 | 48 | 32 | 16 | 2.00 | 0.008010833 | calponin/transgelin |
| contig02811 | 47 | 36 | 11 | 3.27 | 0.000123756 | diapausin |
| contig00324 | 46 | 35 | 11 | 3.18 | 0.000189584 | **lipase** |
| contig04652 | 46 | 31 | 15 | 2.07 | 0.007272245 | methylenetetrahydrofolate dehydrogenase |
| contig01877 | 45 | 31 | 14 | 2.21 | 0.004742769 | regucalcin-like |
| contig03459 | 45 | 31 | 14 | 2.21 | 0.004742769 |  |
| contig06372 | 45 | 31 | 14 | 2.21 | 0.004742769 |  |
| contig02765 | 44 | 30 | 14 | 2.14 | 0.006534481 | serine protease |
| contig03583 | 43 | 31 | 12 | 2.58 | 0.001743806 | Sorbitol dehydrogenase |
| contig03557 | 42 | 35 | 7 | 5.00 | 6.13416E-06 | 15-hydroxyprostaglandin dehydrogenase |
| contig01292 | 42 | 32 | 10 | 3.20 | 0.000334567 |  |
| contig03228 | 42 | 29 | 13 | 2.23 | 0.005802288 | overgrown hematopoietic organs at 23B |
| contig02371 | 42 | 29 | 13 | 2.23 | 0.005802288 |  |

Table S2. Continued.

| Contig # | # T-read | # A-read | # I-read | Fold activation | Binomial probability | Gene prediction |
| --- | --- | --- | --- | --- | --- | --- |
| contig02712 | 42 | 29 | 13 | 2.23 | 0.005802288 |  |
| contig03798 | 41 | 31 | 10 | 3.10 | 0.000509817 |  |
| contig03057 | 41 | 28 | 13 | 2.15 | 0.008012683 |  |
| contig06303 | 41 | 28 | 13 | 2.15 | 0.008012683 |  |
| contig00694 | 39 | 33 | 6 | 5.50 | 5.93468E-06 |  |
| contig05726 | 38 | 34 | 4 | 8.50 | 2.68537E-07 |  |
| contig00502 | 38 | 28 | 10 | 2.80 | 0.001719795 | cysteine synthase |
| contig01831 | 38 | 28 | 10 | 2.80 | 0.001719795 | zinc-containing alcohol dehydrogenase |
| contig00100 | 38 | 26 | 12 | 2.17 | 0.009849737 | ATPase, H+ transporting, lysosomal, V1 subunit C |
| contig02466 | 37 | 28 | 9 | 3.11 | 0.000905155 | endonuclease-reverse transcriptase |
| contig05712 | 37 | 27 | 10 | 2.70 | 0.002534435 |  |
| contig03684 | 37 | 26 | 11 | 2.36 | 0.006220887 | Vacuolar H[+]-ATPase C39 subunit |
| contig03801 | 36 | 26 | 10 | 2.60 | 0.003698906 | methylenetetrahydrofolate dehydrogenase |
| contig01436 | 36 | 25 | 11 | 2.27 | 0.008742868 |  |
| contig02250 | 36 | 25 | 11 | 2.27 | 0.008742868 | CG17272 [*Drosophila melanogaster*] |
| contig01672 | 35 | 25 | 10 | 2.50 | 0.005342864 | NADPH cytochrome P450 reductase |
| contig03527 | 34 | 28 | 6 | 4.67 | 7.82837E-05 | immune related protein [*Spodoptera frugiperda*] |
| contig06007 | 34 | 26 | 8 | 3.25 | 0.00105683 |  |
| contig05289 | 33 | 25 | 8 | 3.13 | 0.001616328 |  |
| contig05477 | 33 | 25 | 8 | 3.13 | 0.001616328 |  |
| contig01552 | 32 | 32 | 0 | ∞ | 2.32831E-10 | nardilysin (N-arginine dibasic convertase) |
| contig05874 | 32 | 30 | 2 | 15.00 | 1.15484E-07 |  |
| contig02739 | 32 | 26 | 6 | 4.33 | 0.000210989 | mitochondrial succinate dehydrogenase cytochrome b subunit |
| contig05932 | 30 | 23 | 7 | 3.29 | 0.001895986 | 3-hydroxyacyl-CoA dehydrogenase |
| contig00907 | 30 | 23 | 7 | 3.29 | 0.001895986 |  |
| contig02108 | 30 | 22 | 8 | 2.75 | 0.005450961 | Ubiquitin-conjugating enzyme protein UbcC |
| contig04823 | 30 | 22 | 8 | 2.75 | 0.005450961 |  |
| contig03654 | 29 | 25 | 4 | 6.25 | 4.42397E-05 |  |
| contig00722 | 29 | 22 | 7 | 3.14 | 0.002907179 |  |
| contig04596 | 29 | 22 | 7 | 3.14 | 0.002907179 |  |
| contig03544 | 29 | 21 | 8 | 2.63 | 0.007994743 | cytochrome P450 |
| contig03866 | 29 | 21 | 8 | 2.63 | 0.007994743 | apoptosis-inducing factor |
| contig03461 | 28 | 24 | 4 | 6.00 | 7.62753E-05 |  |
| contig00623 | 28 | 23 | 5 | 4.60 | 0.000366122 |  |
| contig05882 | 27 | 24 | 3 | 8.00 | 2.17929E-05 |  |

Table S2. Continued.

| Contig # | # T-read | # A-read | # I-read | Fold activation | Binomial probability | Gene prediction |
| --- | --- | --- | --- | --- | --- | --- |
| contig04035 | 27 | 22 | 5 | 4.40 | 0.000601485 |  |
| contig01118 | 27 | 21 | 6 | 3.50 | 0.002205446 | phosphoglycolate phosphatase |
| contig03744 | 27 | 21 | 6 | 3.50 | 0.002205446 |  |
| contig00334 | 27 | 20 | 7 | 2.86 | 0.006616339 |  |
| contig06179 | 26 | 22 | 4 | 5.50 | 0.000222772 | aldehyde dehydrogenase |
| contig02369 | 26 | 19 | 7 | 2.71 | 0.009801984 | CG33998 [*Drosophila melanogaster*] |
| contig00517 | 25 | 21 | 4 | 5.25 | 0.000376999 |  |
| contig01621 | 25 | 20 | 5 | 4.00 | 0.001583397 | alpha actinin |
| contig00432 | 25 | 20 | 5 | 4.00 | 0.001583397 | R06B9.1 [*Caenorhabditis elegans*] |
| contig02348 | 25 | 19 | 6 | 3.17 | 0.005277991 | Ribosomal protein S27A |
| contig03344 | 25 | 19 | 6 | 3.17 | 0.005277991 |  |
| contig03740 | 24 | 21 | 3 | 7.00 | 0.00012064 | synaptic vesicle protein |
| contig00842 | 24 | 20 | 4 | 5.00 | 0.000633359 | ribonuclease L inhibitor |
| contig06342 | 24 | 19 | 5 | 3.80 | 0.002533436 |  |
| contig01308 | 24 | 18 | 6 | 3.00 | 0.008022547 | 4-coumarate-CoA ligase |
| contig02031 | 24 | 18 | 6 | 3.00 | 0.008022547 | Heat-shock-protein-70Ab [*Drosophila melanogaster*] |
| contig00357 | 23 | 23 | 0 | ∞ | 1.19209E-07 | cytochrome P450 |
| contig00278 | 23 | 19 | 4 | 4.75 | 0.001055598 | plexin A |
| contig06367 | 23 | 19 | 4 | 4.75 | 0.001055598 |  |
| contig02234 | 23 | 18 | 5 | 3.60 | 0.004011273 |  |
| contig04484 | 22 | 22 | 0 | ∞ | 2.38419E-07 |  |
| contig03942 | 21 | 18 | 3 | 6.00 | 0.000634193 |  |
| contig00354 | 21 | 16 | 5 | 3.20 | 0.009703159 |  |
| contig01612 | 21 | 16 | 5 | 3.20 | 0.009703159 | oxidoreductase |
| contig03593 | 21 | 16 | 5 | 3.20 | 0.009703159 |  |
| contig01073 | 19 | 17 | 2 | 8.50 | 0.000326157 | CG17928 [*Drosophila melanogaster*] |
| contig02537 | 19 | 16 | 3 | 5.33 | 0.001848221 |  |
| contig02716 | 19 | 15 | 4 | 3.75 | 0.007392883 | CG10320 [*Drosophila melanogaster*] |
| contig05415 | 19 | 15 | 4 | 3.75 | 0.007392883 | acyl-CoA dehydrogenase |
| contig02860 | 18 | 17 | 1 | 17.00 | 6.86646E-05 |  |
| contig02977 | 18 | 15 | 3 | 5.00 | 0.003112793 |  |
| contig00367 | 17 | 16 | 1 | 16.00 | 0.0001297 | defense response protein [*Glossina morsitans morsitans*] |
| contig06079 | 17 | 16 | 1 | 16.00 | 0.0001297 | methylenetetrahydrofolate dehydrogenase |
| contig04702 | 17 | 16 | 1 | 16.00 | 0.0001297 | cytochrome P450 |
| contig06384 | 17 | 15 | 2 | 7.50 | 0.001037598 |  |

Table S2. Continued.

| Contig # | # T-read | # A-read | # I-read | Fold activation | Binomial probability | Gene prediction |
| --- | --- | --- | --- | --- | --- | --- |
| contig03655 | 17 | 14 | 3 | 4.67 | 0.005187988 |  |
| contig03768 | 16 | 16 | 0 | ∞ | 1.52588E-05 | KDEL endoplasmic reticulum protein retention receptor |
| contig03580 | 16 | 16 | 0 | ∞ | 1.52588E-05 |  |
| contig05942 | 16 | 16 | 0 | ∞ | 1.52588E-05 |  |
| contig01304 | 16 | 14 | 2 | 7.00 | 0.001831055 |  |
| contig04482 | 16 | 13 | 3 | 4.33 | 0.008544922 |  |
| contig00152 | 16 | 13 | 3 | 4.33 | 0.008544922 | sugarbabe [*Drosophila melanogaster*] |
| contig02678 | 15 | 14 | 1 | 14.00 | 0.000457764 | serine protease |
| contig01335 | 15 | 14 | 1 | 14.00 | 0.000457764 | 3-hydroxyacyl-coa dehyrogenase |
| contig05025 | 15 | 14 | 1 | 14.00 | 0.000457764 |  |
| contig00628 | 15 | 13 | 2 | 6.50 | 0.003204346 | sugar transporter |
| contig04582 | 14 | 14 | 0 | ∞ | 6.10352E-05 | cytochrome P450 |
| contig04682 | 14 | 13 | 1 | 13.00 | 0.000854492 | isopentenyl-diphosphate delta isomerase |
| contig02803 | 14 | 13 | 1 | 13.00 | 0.000854492 | cytochrome P450 |
| contig03925 | 14 | 13 | 1 | 13.00 | 0.000854492 |  |
| contig01103 | 14 | 12 | 2 | 6.00 | 0.005554199 | protein disulfide isomerase |
| contig00519 | 14 | 12 | 2 | 6.00 | 0.005554199 |  |
| contig01370 | 13 | 12 | 1 | 12.00 | 0.001586914 | protein tyrosine phosphatase |
| contig01517 | 13 | 12 | 1 | 12.00 | 0.001586914 | bent [*Drosophila melanogaster*] |
| contig05921 | 13 | 12 | 1 | 12.00 | 0.001586914 |  |
| contig05560 | 13 | 11 | 2 | 5.50 | 0.009521484 |  |
| contig01182 | 13 | 11 | 2 | 5.50 | 0.009521484 | thioredoxin peroxidase |
| contig01167 | 12 | 12 | 0 | ∞ | 0.000244141 | serine protease |
| contig02675 | 12 | 12 | 0 | ∞ | 0.000244141 | beta-1,4-galactosyltransferase |
| contig01655 | 12 | 12 | 0 | ∞ | 0.000244141 | G31248 [*Drosophila melanogaster*] |
| contig03154 | 12 | 11 | 1 | 11.00 | 0.002929688 |  |
| contig03555 | 12 | 11 | 1 | 11.00 | 0.002929688 |  |
| contig03689 | 12 | 11 | 1 | 11.00 | 0.002929688 |  |
| contig06285 | 12 | 11 | 1 | 11.00 | 0.002929688 |  |
| contig00817 | 11 | 11 | 0 | ∞ | 0.000488281 |  |
| contig05304 | 11 | 11 | 0 | ∞ | 0.000488281 |  |
| contig03004 | 11 | 10 | 1 | 10.00 | 0.005371094 | cecropin |
| contig02669 | 11 | 10 | 1 | 10.00 | 0.005371094 | glutathione S-transferase |
| contig00571 | 11 | 10 | 1 | 10.00 | 0.005371094 |  |
| contig00841 | 11 | 10 | 1 | 10.00 | 0.005371094 |  |

Table S2. Continued.

| Contig # | # T-read | # A-read | # I-read | Fold activation | Binomial probability | Gene prediction |
| --- | --- | --- | --- | --- | --- | --- |
| contig06046 | 11 | 10 | 1 | 10.00 | 0.005371094 |  |
| contig04483 | 10 | 10 | 0 | ∞ | 0.000976563 |  |
| contig02876 | 10 | 9 | 1 | 9.00 | 0.009765625 | diapausin |
| contig06063 | 10 | 9 | 1 | 9.00 | 0.009765625 | odorant binding protein |
| contig01466 | 10 | 9 | 1 | 9.00 | 0.009765625 | alcohol dehydrogenase |
| contig03428 | 10 | 9 | 1 | 9.00 | 0.009765625 | ATP-dependent RNA helicase |
| contig01538 | 10 | 9 | 1 | 9.00 | 0.009765625 |  |
| contig03160 | 10 | 9 | 1 | 9.00 | 0.009765625 |  |
| contig04772 | 10 | 9 | 1 | 9.00 | 0.009765625 |  |
| contig05154 | 10 | 9 | 1 | 9.00 | 0.009765625 |  |
| contig05992 | 10 | 9 | 1 | 9.00 | 0.009765625 |  |
| contig01773 | 10 | 9 | 1 | 9.00 | 0.009765625 |  |
| contig03270 | 9 | 9 | 0 | ∞ | 0.001953125 | eukaryotic initiation factor |
| contig01143 | 9 | 9 | 0 | ∞ | 0.001953125 |  |
| contig02737 | 9 | 9 | 0 | ∞ | 0.001953125 | CG11125 [*Drosophila melanogaster*] |
| contig04602 | 9 | 9 | 0 | ∞ | 0.001953125 |  |
| contig05125 | 9 | 9 | 0 | ∞ | 0.001953125 |  |
| contig05769 | 9 | 9 | 0 | ∞ | 0.001953125 |  |
| contig05833 | 9 | 9 | 0 | ∞ | 0.001953125 |  |
| contig03712 | 8 | 8 | 0 | ∞ | 0.00390625 | ribonucleoside-diphosphate reductase small chain |
| contig04566 | 8 | 8 | 0 | ∞ | 0.00390625 | synaptotagmin |
| contig01075 | 8 | 8 | 0 | ∞ | 0.00390625 |  |
| contig02227 | 8 | 8 | 0 | ∞ | 0.00390625 |  |
| contig02892 | 8 | 8 | 0 | ∞ | 0.00390625 | titin-like protein [Bombyx mori] |
| contig03218 | 8 | 8 | 0 | ∞ | 0.00390625 |  |
| contig06089 | 8 | 8 | 0 | ∞ | 0.00390625 |  |
