# Supplementary material for: Analysis of Genes Expression of Spodoptera exigua Larvae upon AcMNPV Infection
Source: PLoS One. 2012 Jul 31;7(7):e42462. doi: 10.1371/journal.pone.0042462 (PMC3409162; doi:10.1371/journal.pone.0042462)
Supplement: Table S3 — List of S. exigua genes significantly down-regulated by active AcMNPV infection. (DOC) [file pone.0042462.s003.doc]

Table S3. List of *S. exigua* genes significantly down-regulated by active AcMNP infection.

| Contig # | # T-read | # A-read | # I-read | Fold activation | Binomial probability | Gene prediction |
| --- | --- | --- | --- | --- | --- | --- |
| contig05994 | 905 | 328 | 577 | 1.76 | 2.34601E-17 | Lys |
| contig05861 | 820 | 311 | 509 | 1.64 | 9.39431E-13 | hexamerin |
| contig04967 | 478 | 156 | 322 | 2.06 | 6.41259E-15 | Glutathione S transferase |
| contig05896 | 400 | 144 | 256 | 1.78 | 5.20841E-09 | diapausin |
| contig05789 | 372 | 130 | 242 | 1.86 | 1.58428E-09 |  |
| contig00209 | 166 | 42 | 124 | 2.95 | 4.5488E-11 | **larval cuticular protein 1 [*Helicoverpa armigera*], LCP-1** |
| contig00304 | 157 | 49 | 108 | 2.20 | 7.96837E-07 | **profilin** |
| contig05985 | 155 | 49 | 106 | 2.16 | 1.50388E-06 | **DEAD box RNA helicase** |
| contig03280 | 131 | 25 | 106 | 4.24 | 1.75024E-13 |  |
| contig00181 | 129 | 50 | 79 | 1.58 | 0.002686382 |  |
| contig04129 | 128 | 49 | 79 | 1.61 | 0.002082467 | Ribosomal protein L26 |
| contig06242 | 127 | 48 | 79 | 1.65 | 0.001594388 | hexamerin |
| contig00359 | 126 | 39 | 87 | 2.23 | 6.48577E-06 | **glutamine synthetase** |
| contig04140 | 120 | 40 | 80 | 2.00 | 8.6183E-05 | **Tetraspanin 39D [*Drosophila melanogaster* ]** |
| contig00125 | 112 | 10 | 102 | 10.20 | 1.08996E-20 | **ornithine aminotransferase, Ornithine AT** |
| contig03433 | 112 | 43 | 69 | 1.60 | 0.003678286 | tubulin, beta 2c |
| contig00611 | 111 | 43 | 68 | 1.58 | 0.004532174 | Ribosomal protein L8 |
| contig00006 | 110 | 44 | 66 | 1.50 | 0.008455603 | Calmodulin |
| contig05431 | 109 | 37 | 72 | 1.95 | 0.000263951 | serine protease |
| contig05842 | 109 | 39 | 70 | 1.79 | 0.000910469 |  |
| contig06163 | 103 | 39 | 64 | 1.64 | 0.003772862 |  |
| contig05517 | 100 | 38 | 62 | 1.63 | 0.00447288 | Ribosomal protein S26 |
| contig04060 | 99 | 34 | 65 | 1.91 | 0.000604699 | peptidylprolyl isomerase F (cyclophilin F) |
| contig04227 | 98 | 26 | 72 | 2.77 | 1.20452E-06 | **chemosensory protein, CSP** |
| contig01569 | 98 | 35 | 63 | 1.80 | 0.001451976 | stathmin |
| contig05174 | 97 | 35 | 62 | 1.77 | 0.001866826 | jaculatory bulb protein |
| contig05810 | 96 | 28 | 68 | 2.43 | 1.65536E-05 | **lysozyme** |
| contig01245 | 96 | 32 | 64 | 2.00 | 0.000374889 | **calreticulin** |
| contig00174 | 95 | 32 | 63 | 1.97 | 0.000499852 | thymosin |
| contig00429 | 94 | 0 | 94 | ∞ | 5.04871E-29 | **hexamerin** |
| contig03564 | 93 | 30 | 63 | 2.10 | 0.000222107 | acyl-CoA binding protein |
| contig01066 | 91 | 33 | 58 | 1.76 | 0.00267541 | Ribosomal protein L13A |
| contig05857 | 90 | 35 | 55 | 1.57 | 0.00914793 | Ribosomal protein L27A |
| contig00122 | 89 | 29 | 60 | 2.07 | 0.000362502 | sterol carrier protein 2/3-oxoacyl-CoA thiolase |
| contig00254 | 89 | 34 | 55 | 1.62 | 0.007115056 | dCMP deaminase |

Table S3. Continued.

| Contig # | # T-read | # A-read | # I-read | Fold activation | Binomial probability | Gene prediction |
| --- | --- | --- | --- | --- | --- | --- |
| contig05286 | 88 | 26 | 62 | 2.38 | 4.72224E-05 | chemosensory protein |
| contig01948 | 87 | 27 | 60 | 2.22 | 0.000150333 | bifunctional purine biosynthesis protein |
| contig03566 | 84 | 28 | 56 | 2.00 | 0.000790414 | zinc finger protein |
| contig01821 | 84 | 30 | 54 | 1.80 | 0.002798248 | Ribosomal protein S11 |
| contig05906 | 83 | 30 | 53 | 1.77 | 0.003597748 | mitochondrial NADH-ubiquinone oxidoreductase |
| contig03729 | 81 | 27 | 54 | 2.00 | 0.000953855 |  |
| contig03558 | 79 | 23 | 56 | 2.43 | 8.05202E-05 | Pupal cuticle protein |
| contig00450 | 78 | 28 | 50 | 1.79 | 0.004040688 | ADP ribosylation factor |
| contig00371 | 77 | 26 | 51 | 1.96 | 0.001535827 | ribosomal protein S13 |
| contig03983 | 77 | 26 | 51 | 1.96 | 0.001535827 | vitellogenin |
| contig00243 | 76 | 22 | 54 | 2.45 | 9.61747E-05 | phosphoserine phosphatase |
| contig00850 | 76 | 22 | 54 | 2.45 | 9.61747E-05 | superoxide dismutase |
| contig00988 | 76 | 24 | 52 | 2.17 | 0.000498645 | cytochrome c oxidase subunit Va |
| contig00456 | 76 | 27 | 49 | 1.81 | 0.003767541 | protein disulfide isomerase |
| contig00189 | 74 | 15 | 59 | 3.93 | 9.65623E-08 |  |
| contig00398 | 74 | 26 | 48 | 1.85 | 0.003497864 | phosphoribosylaminoimidazole carboxylase |
| contig03328 | 73 | 11 | 62 | 5.64 | 3.76771E-10 |  |
| contig06280 | 72 | 20 | 52 | 2.60 | 6.6079E-05 | chemosensory protein |
| contig03481 | 71 | 20 | 51 | 2.55 | 9.54474E-05 |  |
| contig03757 | 70 | 25 | 45 | 1.80 | 0.005468243 | Lipid storage droplet |
| contig03571 | 69 | 17 | 52 | 3.06 | 1.01046E-05 | **juvenile hormone binding protein, JHBP1** |
| contig02365 | 68 | 24 | 44 | 1.83 | 0.005094636 | Heat shock protein cognate 3 [*Drosophila melanogaster* ] |
| contig05625 | 67 | 23 | 44 | 1.91 | 0.003596214 |  |
| contig01280 | 62 | 21 | 41 | 1.95 | 0.003992674 | Enolase |
| contig01227 | 61 | 10 | 51 | 5.10 | 3.91081E-08 | chemosensory protein |
| contig06401 | 60 | 14 | 46 | 3.29 | 1.50452E-05 |  |
| contig01789 | 60 | 20 | 40 | 2.00 | 0.003635846 | cystathionase, cystathionine gamma-lyase |
| contig03265 | 58 | 1 | 57 | 57.00 | 2.01228E-16 |  |
| contig04097 | 57 | 0 | 57 | ∞ | 6.93889E-18 |  |
| contig02353 | 57 | 15 | 42 | 2.80 | 0.000153058 | alanine aminotransferase |
| contig01319 | 57 | 19 | 38 | 2.00 | 0.004419964 | scavenger receptor cysteine-rich protein |
| contig01147 | 57 | 20 | 37 | 1.85 | 0.008397932 | Isocitrate dehydrogenase |
| contig03358 | 56 | 10 | 46 | 4.60 | 4.94147E-07 | purine nucleoside phosphorylase |
| contig01100 | 56 | 18 | 38 | 2.11 | 0.002946643 | chemosensory protein |
| contig04842 | 56 | 19 | 37 | 1.95 | 0.005893286 | Saposin-related [*Drosophila melanogaster*] |

Table S3. Continued.

| Contig # | # T-read | # A-read | # I-read | Fold activation | Binomial probability | Gene prediction |
| --- | --- | --- | --- | --- | --- | --- |
| contig04946 | 55 | 17 | 38 | 2.24 | 0.00189427 | transketolase |
| contig05707 | 53 | 13 | 40 | 3.08 | 9.34134E-05 | aldehyde dehydrogenase |
| contig03341 | 53 | 17 | 36 | 2.12 | 0.003586995 |  |
| contig05406 | 52 | 5 | 47 | 9.40 | 5.77085E-10 | cuticular protein |
| contig00133 | 52 | 16 | 36 | 2.25 | 0.002301091 | peptidylprolyl isomerase |
| contig01170 | 52 | 16 | 36 | 2.25 | 0.002301091 | ribosomal protein L17 |
| contig01582 | 51 | 16 | 35 | 2.19 | 0.003186127 | sparc [*Drosophila melanogaster*] |
| contig00126 | 50 | 9 | 41 | 4.56 | 2.22527E-06 | glutathione S transferase |
| contig03561 | 49 | 15 | 34 | 2.27 | 0.002798794 | sterol carrier protein 2/3-oxoacyl-CoA thiolase |
| contig04173 | 48 | 16 | 32 | 2.00 | 0.008010833 |  |
| contig01157 | 47 | 6 | 41 | 6.83 | 7.6295E-08 | cuticular protein RR-1 motif |
| contig00607 | 47 | 11 | 36 | 3.27 | 0.000123756 | cytochrome P450 |
| contig00267 | 47 | 13 | 34 | 2.62 | 0.000999569 | carboxypeptidase |
| contig02051 | 45 | 6 | 39 | 6.50 | 2.31497E-07 | cuticular protein |
| contig00124 | 44 | 3 | 41 | 13.67 | 7.52834E-10 |  |
| contig00609 | 44 | 6 | 38 | 6.33 | 4.01261E-07 | cytochrome P450 |
| contig05650 | 44 | 13 | 31 | 2.38 | 0.002951056 | NADPH-specific isocitrate dehydrogenase |
| contig00520 | 43 | 8 | 35 | 4.38 | 1.64856E-05 | **gloverin, GLV1** |
| contig01343 | 43 | 11 | 32 | 2.91 | 0.000653927 | Heat shock protein 60 [*Drosophila melanogaster*] |
| contig05379 | 43 | 12 | 31 | 2.58 | 0.001743806 | repat |
| contig01675 | 43 | 12 | 31 | 2.58 | 0.001743806 |  |
| contig02683 | 43 | 13 | 30 | 2.31 | 0.004158306 | angiotensin converting enzyme |
| contig05879 | 43 | 14 | 29 | 2.07 | 0.008910656 |  |
| contig04184 | 41 | 6 | 35 | 5.83 | 2.04472E-06 | **gloverin, GLV2** |
| contig01969 | 41 | 11 | 30 | 2.73 | 0.001436757 | serine protease |
| contig06203 | 41 | 12 | 29 | 2.42 | 0.003591892 | **attacin, ATT1** |
| contig05002 | 40 | 11 | 29 | 2.64 | 0.002102571 | **juvenile hormone binding protein, JHBP2** |
| contig05017 | 39 | 7 | 32 | 4.57 | 2.79778E-05 | Kazal-type serine protease inhibitor |
| contig00795 | 39 | 10 | 29 | 2.90 | 0.001156414 | immune-related Hdd1 [*Hyphantria cunea*] |
| contig06019 | 38 | 12 | 26 | 2.17 | 0.009849737 |  |
| contig00046 | 37 | 10 | 27 | 2.70 | 0.002534435 |  |
| contig02989 | 37 | 11 | 26 | 2.36 | 0.006220887 | CG5023 [*Drosophila melanogaster*] |
| contig02331 | 36 | 8 | 28 | 3.50 | 0.000440346 | beta-N-acetylglucosaminidase |
| contig00428 | 35 | 1 | 34 | 34.00 | 1.01863E-09 |  |
| contig06269 | 34 | 0 | 34 | ∞ | 5.82077E-11 |  |

Table S3. Continued.

| Contig # | # T-read | # A-read | # I-read | Fold activation | Binomial probability | Gene prediction |
| --- | --- | --- | --- | --- | --- | --- |
| contig04100 | 34 | 9 | 25 | 2.78 | 0.003053065 | Gm2263 [*Mus musculus*] |
| contig00341 | 34 | 10 | 24 | 2.40 | 0.007632662 | unc-52 [*Caenorhabditis elegans*] |
| contig04182 | 33 | 5 | 28 | 5.60 | 2.76295E-05 |  |
| contig02897 | 33 | 6 | 27 | 4.50 | 0.000128938 | DEAD-box ATPase |
| contig05024 | 32 | 5 | 27 | 5.40 | 4.68865E-05 |  |
| contig01021 | 31 | 9 | 22 | 2.44 | 0.009387766 | innexin |
| contig00123 | 30 | 1 | 29 | 29.00 | 2.79397E-08 |  |
| contig01992 | 30 | 2 | 28 | 14.00 | 4.05125E-07 | **juvenile hormone binding protein, JHBP3** |
| contig03419 | 30 | 6 | 24 | 4.00 | 0.000552996 | glucose dehydrogenase |
| contig02264 | 30 | 6 | 24 | 4.00 | 0.000552996 |  |
| contig03896 | 30 | 7 | 23 | 3.29 | 0.001895986 | H+ transporting ATP synthase beta subunit |
| contig00971 | 30 | 8 | 22 | 2.75 | 0.005450961 |  |
| contig06075 | 29 | 7 | 22 | 3.14 | 0.002907179 |  |
| contig03730 | 28 | 4 | 24 | 6.00 | 7.62753E-05 |  |
| contig04213 | 28 | 7 | 21 | 3.00 | 0.004410893 |  |
| contig04503 | 27 | 4 | 23 | 5.75 | 0.000130758 | glutathione S-transferase |
| contig02434 | 27 | 7 | 20 | 2.86 | 0.006616339 |  |
| contig05035 | 27 | 7 | 20 | 2.86 | 0.006616339 |  |
| contig03736 | 26 | 0 | 26 | ∞ | 1.49012E-08 |  |
| contig04183 | 26 | 4 | 22 | 5.50 | 0.000222772 | gloverin, GLV3 |
| contig02799 | 26 | 5 | 21 | 4.20 | 0.000980198 | dimeric dihydrodiol dehydrogenase |
| contig04193 | 26 | 6 | 20 | 3.33 | 0.003430694 | Ecdysteroid-regulated [*Pediculus humanus corporis*] |
| contig01180 | 26 | 7 | 19 | 2.71 | 0.009801984 |  |
| contig03796 | 25 | 4 | 21 | 5.25 | 0.000376999 | juvenile hormone diol kinase |
| contig00332 | 25 | 6 | 19 | 3.17 | 0.005277991 |  |
| contig02086 | 25 | 6 | 19 | 3.17 | 0.005277991 | zgc:113227 [*Danio rerio*] |
| contig02556 | 25 | 6 | 19 | 3.17 | 0.005277991 |  |
| contig03494 | 24 | 2 | 22 | 11.00 | 1.64509E-05 | amidophosphoribosyltransferase |
| contig01013 | 24 | 5 | 19 | 3.80 | 0.002533436 | type IV collagen |
| contig04688 | 24 | 6 | 18 | 3.00 | 0.008022547 | Pheromone-binding protein-related |
| contig04710 | 23 | 2 | 21 | 10.50 | 3.016E-05 | glucosamine-6-phosphate isomerase |
| contig00269 | 23 | 4 | 19 | 4.75 | 0.001055598 | protein disulfide isomerase associated 6; Pdia6 |
| contig03946 | 22 | 4 | 18 | 4.50 | 0.001744032 | chemosensory protein |
| contig01553 | 22 | 5 | 17 | 3.40 | 0.006278515 | CG33306 [*Drosophila melanogaster*] |
| contig03957 | 22 | 5 | 17 | 3.40 | 0.006278515 | CG4367 [*Drosophila melanogaster*] |

Table S3. Continued.

| Contig # | # T-read | # A-read | # I-read | Fold activation | Binomial probability | Gene prediction |
| --- | --- | --- | --- | --- | --- | --- |
| contig00441 | 21 | 5 | 16 | 3.20 | 0.009703159 |  |
| contig01098 | 21 | 5 | 16 | 3.20 | 0.009703159 | adenylate cyclase-associated protein |
| contig01757 | 21 | 5 | 16 | 3.20 | 0.009703159 | CG5885 [*Drosophila melanogaster*] |
| contig05982 | 21 | 5 | 16 | 3.20 | 0.009703159 |  |
| contig04169 | 20 | 1 | 19 | 19.00 | 1.90735E-05 |  |
| contig02411 | 20 | 2 | 18 | 9.00 | 0.000181198 | synaptic vesicle protein |
| contig00248 | 20 | 4 | 16 | 4.00 | 0.004620552 |  |
| contig06087 | 20 | 4 | 16 | 4.00 | 0.004620552 |  |
| contig04485 | 19 | 3 | 16 | 5.33 | 0.001848221 | serine protease |
| contig00173 | 19 | 3 | 16 | 5.33 | 0.001848221 | thymosin |
| contig05407 | 19 | 4 | 15 | 3.75 | 0.007392883 | cuticular protein |
| contig06142 | 18 | 0 | 18 | ∞ | 3.8147E-06 |  |
| contig04017 | 18 | 3 | 15 | 5.00 | 0.003112793 | prophenoloxidase |
| contig02605 | 18 | 3 | 15 | 5.00 | 0.003112793 |  |
| contig03549 | 18 | 3 | 15 | 5.00 | 0.003112793 | adhesion-like transmembrane protein [*Spiroplasma citri*] |
| contig03203 | 17 | 0 | 17 | ∞ | 7.62939E-06 |  |
| contig06224 | 17 | 0 | 17 | ∞ | 7.62939E-06 |  |
| contig00825 | 17 | 1 | 16 | 16.00 | 0.0001297 | cecropin |
| contig00260 | 17 | 2 | 15 | 7.50 | 0.001037598 |  |
| contig06025 | 17 | 2 | 15 | 7.50 | 0.001037598 |  |
| contig03884 | 17 | 3 | 14 | 4.67 | 0.005187988 | endopeptidase inhibitor |
| contig05563 | 17 | 3 | 14 | 4.67 | 0.005187988 |  |
| contig06053 | 17 | 3 | 14 | 4.67 | 0.005187988 |  |
| contig01346 | 16 | 0 | 16 | ∞ | 1.52588E-05 | chitinase |
| contig00763 | 16 | 0 | 16 | ∞ | 1.52588E-05 | serine protease |
| contig02355 | 16 | 1 | 15 | 15.00 | 0.000244141 |  |
| contig05970 | 16 | 2 | 14 | 7.00 | 0.001831055 |  |
| contig01814 | 16 | 3 | 13 | 4.33 | 0.008544922 | serine protease |
| contig01154 | 16 | 3 | 13 | 4.33 | 0.008544922 | MYO1 [*Candida albicans*] |
| contig02782 | 16 | 3 | 13 | 4.33 | 0.008544922 | CG5001 [*Drosophila melanogaster*] |
| contig02918 | 16 | 3 | 13 | 4.33 | 0.008544922 | RIKEN cDNA 4933439F18 gene [*Mus musculus*] |
| contig03367 | 16 | 3 | 13 | 4.33 | 0.008544922 |  |
| contig05887 | 16 | 3 | 13 | 4.33 | 0.008544922 |  |
| contig05015 | 15 | 0 | 15 | ∞ | 3.05176E-05 |  |
| contig05544 | 15 | 1 | 14 | 14.00 | 0.000457764 |  |

Table S3. Continued.

| Contig # | # T-read | # A-read | # I-read | Fold activation | Binomial probability | Gene prediction |
| --- | --- | --- | --- | --- | --- | --- |
| contig04016 | 15 | 2 | 13 | 6.50 | 0.003204346 |  |
| contig03977 | 14 | 1 | 13 | 13.00 | 0.000854492 |  |
| contig02694 | 14 | 2 | 12 | 6.00 | 0.005554199 | cytochrome P450 |
| contig01871 | 14 | 2 | 12 | 6.00 | 0.005554199 | cysteine sulfinic acid decarboxylase |
| contig04639 | 13 | 0 | 13 | ∞ | 0.00012207 | Neural Lazarillo |
| contig03660 | 13 | 1 | 12 | 12.00 | 0.001586914 | vitellogenin-like |
| contig02881 | 13 | 1 | 12 | 12.00 | 0.001586914 | cuticular protein |
| contig03100 | 13 | 1 | 12 | 12.00 | 0.001586914 |  |
| contig05576 | 13 | 1 | 12 | 12.00 | 0.001586914 |  |
| contig02477 | 13 | 2 | 11 | 5.50 | 0.009521484 | serine protease |
| contig03442 | 13 | 2 | 11 | 5.50 | 0.009521484 | chemosensory protein |
| contig03161 | 13 | 2 | 11 | 5.50 | 0.009521484 | glutathione S transferase |
| contig00466 | 13 | 2 | 11 | 5.50 | 0.009521484 |  |
| contig03186 | 13 | 2 | 11 | 5.50 | 0.009521484 | proteasome alpha 3 subunit |
| contig03789 | 13 | 2 | 11 | 5.50 | 0.009521484 | CG33966 [*Drosophila melanogaster*] |
| contig04400 | 13 | 2 | 11 | 5.50 | 0.009521484 | carboxypeptidase, vitellogenic-like |
| contig06115 | 13 | 2 | 11 | 5.50 | 0.009521484 |  |
| contig02220 | 12 | 0 | 12 | ∞ | 0.000244141 | serine protease |
| contig00622 | 12 | 0 | 12 | ∞ | 0.000244141 | lipase |
| contig03017 | 12 | 0 | 12 | ∞ | 0.000244141 |  |
| contig02422 | 12 | 1 | 11 | 11.00 | 0.002929688 | fucosidase, alpha-L- 1 |
| contig00845 | 11 | 0 | 11 | ∞ | 0.000488281 | 18 kda serine proteinase inhibitor [Helicoverpa zea] |
| contig02324 | 11 | 0 | 11 | ∞ | 0.000488281 | laminin |
| contig04577 | 11 | 1 | 10 | 10.00 | 0.005371094 | glycosyl hydrolase |
| contig04025 | 11 | 1 | 10 | 10.00 | 0.005371094 | CG4367 [*Drosophila melanogaster*] |
| contig02806 | 10 | 0 | 10 | ∞ | 0.000976563 | cecropin |
| contig02635 | 10 | 0 | 10 | ∞ | 0.000976563 | amma interferon inducible lysosomal thiol reductase |
| contig05439 | 10 | 0 | 10 | ∞ | 0.000976563 |  |
| contig02965 | 10 | 1 | 9 | 9.00 | 0.009765625 | NADH-ubiquinone oxidoreductase |
| contig03993 | 10 | 1 | 9 | 9.00 | 0.009765625 | glycolipid transfer protein [*Danio rerio*] |
| contig01730 | 10 | 1 | 9 | 9.00 | 0.009765625 | Translocase of inner membrane 10 [*Drosophila melanogaster*] |
| contig02996 | 10 | 1 | 9 | 9.00 | 0.009765625 | 26S proteasome non-ATPase regulatory subunit |
| contig04606 | 10 | 1 | 9 | 9.00 | 0.009765625 | cuticular protein |
| contig01920 | 10 | 1 | 9 | 9.00 | 0.009765625 |  |
| contig02673 | 10 | 1 | 9 | 9.00 | 0.009765625 |  |

Table S3. Continued.

| Contig # | # T-read | # A-read | # I-read | Fold activation | Binomial probability | Gene prediction |
| --- | --- | --- | --- | --- | --- | --- |
| contig03211 | 10 | 1 | 9 | 9.00 | 0.009765625 |  |
| contig05742 | 10 | 1 | 9 | 9.00 | 0.009765625 |  |
| contig05936 | 10 | 1 | 9 | 9.00 | 0.009765625 |  |
| contig05937 | 10 | 1 | 9 | 9.00 | 0.009765625 |  |
| contig03155 | 9 | 0 | 9 | ∞ | 0.001953125 | cecropin |
| contig02060 | 9 | 0 | 9 | ∞ | 0.001953125 | HMG176 [Helicoverpa armigera] |
| contig01362 | 9 | 0 | 9 | ∞ | 0.001953125 | K homology RNA-binding protein |
| contig00198 | 9 | 0 | 9 | ∞ | 0.001953125 |  |
| contig03115 | 9 | 0 | 9 | ∞ | 0.001953125 | transthyretin-like periplasmic protein |
| contig03214 | 9 | 0 | 9 | ∞ | 0.001953125 |  |
| contig05327 | 9 | 0 | 9 | ∞ | 0.001953125 |  |
| contig06340 | 9 | 0 | 9 | ∞ | 0.001953125 |  |
| contig05691 | 8 | 0 | 8 | ∞ | 0.00390625 | Basic juvenile hormone-suppressible protein |
| contig00820 | 8 | 0 | 8 | ∞ | 0.00390625 | multiple C2 domains, transmembrane 2 [*Rattus norvegicus*] |
| contig01712 | 8 | 0 | 8 | ∞ | 0.00390625 | Kazal-type serine proteinase inhibitor |
| contig02955 | 8 | 0 | 8 | ∞ | 0.00390625 | serine protease |
| contig00948 | 8 | 0 | 8 | ∞ | 0.00390625 | sugar transporter |
| contig01398 | 8 | 0 | 8 | ∞ | 0.00390625 | zinc carboxypeptidase |
| contig02296 | 8 | 0 | 8 | ∞ | 0.00390625 | glycogen synthase kinase 3 beta |
| contig01460 | 8 | 0 | 8 | ∞ | 0.00390625 |  |
| contig02925 | 8 | 0 | 8 | ∞ | 0.00390625 | folliculin [Homo sapiens] |
| contig02954 | 8 | 0 | 8 | ∞ | 0.00390625 |  |
| contig03924 | 8 | 0 | 8 | ∞ | 0.00390625 |  |
| contig04208 | 8 | 0 | 8 | ∞ | 0.00390625 |  |
