# Supplementary material for: Analysis of Genes Expression of Spodoptera exigua Larvae upon AcMNPV Infection
Source: PLoS One. 2012 Jul 31;7(7):e42462. doi: 10.1371/journal.pone.0042462 (PMC3409162; doi:10.1371/journal.pone.0042462)
Supplement: Table S4 — Number of read sequences originating from AcMNPV. (DOC) [file pone.0042462.s004.doc]

Table S4. Number of read sequences originated from AcMNPV.

| Gene | Protein | Accession # | # A-read | # I-read | gene length |
| --- | --- | --- | --- | --- | --- |
| Ac1 | protein tyrosine phosphatase | [NP_054030.1](http://www.ncbi.nlm.nih.gov/protein/9627743) | 0 | 0 | 507 |
| Ac2 | baculovirus repeated ORF | [NP_054031.1](http://www.ncbi.nlm.nih.gov/protein/9627744) | 15 | 0 | 987 |
| Ac3 | conotoxin-like peptide | [NP_054032.1](http://www.ncbi.nlm.nih.gov/protein/9627745) | 4 | 0 | 162 |
| Ac4 | AcOrf-4 peptide | [NP_054033.1](http://www.ncbi.nlm.nih.gov/protein/9627746) | 4 | 1 | 456 |
| Ac5 | AcOrf-5 peptide | [NP_054034.1](http://www.ncbi.nlm.nih.gov/protein/9627747) | 1 | 0 | 329 |
| Ac6 | late expression factor 2 | [NP_054035.1](http://www.ncbi.nlm.nih.gov/protein/9627748) | 8 | 0 | 633 |
| Ac7 | hypothetical protein | [NP_054036.1](http://www.ncbi.nlm.nih.gov/protein/9627749) | 0 | 0 | 606 |
| Ac8 | major occlusion body protein | [NP_054037.1](http://www.ncbi.nlm.nih.gov/protein/9627750) | 1 | 0 | 783 |
| Ac9 | viral capsid associated protein | [NP_054038.1](http://www.ncbi.nlm.nih.gov/protein/9627751) | 3 | 0 | 1632 |
| Ac10 | protein kinase | [NP_054039.1](http://www.ncbi.nlm.nih.gov/protein/9627752) | 2 | 0 | 819 |
| Ac11 | AcOrf-11 peptide | [NP_054040.1](http://www.ncbi.nlm.nih.gov/protein/9627753) | 13 | 0 | 1023 |
| Ac12 | AcOrf-12 peptide | [NP_054041.1](http://www.ncbi.nlm.nih.gov/protein/9627754) | 2 | 0 | 654 |
| Ac13 | AcOrf-13 peptide | [NP_054042.1](http://www.ncbi.nlm.nih.gov/protein/9627755) | 13 | 0 | 984 |
| Ac14 | late expression factor 1 | [NP_054043.1](http://www.ncbi.nlm.nih.gov/protein/9627756) | 1 | 0 | 801 |
| Ac15 | ecdysteroid UDP-glucosyl transferase | [NP_054044.1](http://www.ncbi.nlm.nih.gov/protein/9627757) | 20 | 0 | 1521 |
| Ac16 | AcOrf-16 peptide | [NP_054045.1](http://www.ncbi.nlm.nih.gov/protein/9627758) | 4 | 0 | 678 |
| Ac17 | AcOrf-17 peptide | [NP_054046.1](http://www.ncbi.nlm.nih.gov/protein/9627759) | 5 | 0 | 495 |
| Ac18 | AcOrf-18 peptide | [NP_054047.1](http://www.ncbi.nlm.nih.gov/protein/9627760) | 2 | 0 | 1062 |
| Ac19 | AcOrf-19 peptide | [NP_054048.1](http://www.ncbi.nlm.nih.gov/protein/9627761) | 0 | 0 | 327 |
| Ac20 | actin rearrangement inducing factor | [NP_054049.1](http://www.ncbi.nlm.nih.gov/protein/9627762) | 1 | 0 | 210 |
| Ac21 | actin rearrangement inducing factor | [NP_054050.1](http://www.ncbi.nlm.nih.gov/protein/9627763) | 0 | 0 | 960 |
| Ac22 | AcOrf-22 peptide | [NP_054051.1](http://www.ncbi.nlm.nih.gov/protein/9627764) | 0 | 0 | 1149 |
| Ac23 | copia-like envelope protein | [NP_054052.1](http://www.ncbi.nlm.nih.gov/protein/9627765) | 4 | 0 | 2073 |
| Ac24 | protein kinase interacting protein | [NP_054053.1](http://www.ncbi.nlm.nih.gov/protein/9627766) | 1 | 0 | 510 |
| Ac25 | ssDNA binding protein | [NP_054054.1](http://www.ncbi.nlm.nih.gov/protein/9627767) | 20 | 0 | 951 |
| Ac26 | AcOrf-26 peptide | [NP_054055.1](http://www.ncbi.nlm.nih.gov/protein/9627768) | 0 | 0 | 390 |
| Ac27 | apoptosis inhibitor | [NP_054056.1](http://www.ncbi.nlm.nih.gov/protein/9627769) | 0 | 0 | 861 |
| Ac28 | late expression factor 6 | [NP_054057.1](http://www.ncbi.nlm.nih.gov/protein/9627770) | 4 | 0 | 522 |
| Ac29 | AcOrf-29 peptide | [NP_054058.1](http://www.ncbi.nlm.nih.gov/protein/9627771) | 1 | 0 | 216 |
| Ac30 | AcOrf-30 peptide | [NP_054059.1](http://www.ncbi.nlm.nih.gov/protein/9627772) | 3 | 0 | 1392 |
| Ac31 | superoxide dismutase | [NP_054060.1](http://www.ncbi.nlm.nih.gov/protein/9627773) | 0 | 0 | 456 |
| Ac32 | fibroblast growth factor | [NP_054061.1](http://www.ncbi.nlm.nih.gov/protein/9627774) | 4 | 0 | 546 |
| Ac33 | putative histidinol-phosphatase | [NP_054062.1](http://www.ncbi.nlm.nih.gov/protein/9627775) | 0 | 0 | 549 |
| Ac34 | AcOrf-34 peptide | [NP_054063.1](http://www.ncbi.nlm.nih.gov/protein/9627776) | 0 | 0 | 648 |
| Ac35 | viral ubiquitin | [NP_054064.1](http://www.ncbi.nlm.nih.gov/protein/9627777) | 0 | 0 | 234 |
| Ac36 | nuclear matrix associated phosphoprotein | [NP_054065.1](http://www.ncbi.nlm.nih.gov/protein/9627778) | 15 | 0 | 828 |
| Ac37 | late expression factor 11 | [NP_054066.1](http://www.ncbi.nlm.nih.gov/protein/9627779) | 0 | 0 | 339 |
| Ac38 | AcOrf-38 peptide | [NP_054067.1](http://www.ncbi.nlm.nih.gov/protein/9627780) | 8 | 0 | 651 |
| Ac39 | hypothetical protein | [NP_054068.1](http://www.ncbi.nlm.nih.gov/protein/9627781) | 2 | 0 | 1092 |
| Ac40 | transcription regulator | [NP_054069.1](http://www.ncbi.nlm.nih.gov/protein/9627782) | 0 | 0 | 1206 |
| Ac41 | AcOrf-41 peptide | [NP_054070.1](http://www.ncbi.nlm.nih.gov/protein/9627783) | 0 | 0 | 546 |
| Ac42 | global transactivator-like protein | [NP_054071.1](http://www.ncbi.nlm.nih.gov/protein/9627784) | 0 | 0 | 1521 |
| Ac43 | AcOrf-43 peptide | [NP_054072.1](http://www.ncbi.nlm.nih.gov/protein/9627785) | 0 | 0 | 234 |
| Ac44 | AcOrf-44 peptide | [NP_054073.1](http://www.ncbi.nlm.nih.gov/protein/9627786) | 0 | 0 | 396 |
| Ac45 | AcOrf-45 peptide | [NP_054074.1](http://www.ncbi.nlm.nih.gov/protein/9627787) | 0 | 0 | 579 |
| Ac46 | occlusion-derived virus envelope protein | [NP_054075.1](http://www.ncbi.nlm.nih.gov/protein/9627788) | 0 | 0 | 2115 |
| Ac47 | AcOrf-47 peptide | [NP_054076.1](http://www.ncbi.nlm.nih.gov/protein/9627789) | 6 | 0 | 267 |
| Ac48 | AcOrf-48 peptide | [NP_054077.1](http://www.ncbi.nlm.nih.gov/protein/9627790) | 4 | 0 | 342 |
| Ac49 | proliferating cell nuclear antigen | [NP_054078.1](http://www.ncbi.nlm.nih.gov/protein/9627791) | 4 | 0 | 771 |
| Ac50 | late expression factor 8 | [NP_054079.1](http://www.ncbi.nlm.nih.gov/protein/9627792) | 1 | 0 | 2631 |
| Ac51 | AcOrf-51 peptide | [NP_054080.1](http://www.ncbi.nlm.nih.gov/protein/9627793) | 0 | 0 | 957 |
| Ac52 | AcOrf-52 peptide | [NP_054081.1](http://www.ncbi.nlm.nih.gov/protein/9627794) | 0 | 0 | 372 |

Table S4. Continued.

| Gene | Protein | Accession # | # A-read | # I-read | gene length |
| --- | --- | --- | --- | --- | --- |
| Ac53 | AcOrf-53 peptide | [NP_054082.1](http://www.ncbi.nlm.nih.gov/protein/9627795) | 0 | 0 | 420 |
| Ac53A | late expression factor 10 (53A) | [NP_054083.1](http://www.ncbi.nlm.nih.gov/protein/9627796) | 2 | 0 | 237 |
| Ac54 | viral capsid associated protein | [NP_054084.1](http://www.ncbi.nlm.nih.gov/protein/9627797) | 0 | 0 | 1098 |
| Ac55 | AcOrf-55 peptide | [NP_054085.1](http://www.ncbi.nlm.nih.gov/protein/9627798) | 0 | 0 | 222 |
| Ac56 | AcOrf-56 peptide | [NP_054086.1](http://www.ncbi.nlm.nih.gov/protein/9627799) | 0 | 0 | 255 |
| Ac57 | AcOrf-57 peptide | [NP_054087.1](http://www.ncbi.nlm.nih.gov/protein/9627800) | 0 | 0 | 486 |
| Ac58 | AcOrf-58 peptide | [NP_054088.1](http://www.ncbi.nlm.nih.gov/protein/9627801) | 0 | 0 | 174 |
| Ac59 | AcOrf-59 peptide | [NP_054089.1](http://www.ncbi.nlm.nih.gov/protein/9627802) | 0 | 0 | 210 |
| Ac60 | AcOrf-60 peptide | [NP_054090.1](http://www.ncbi.nlm.nih.gov/protein/9627803) | 0 | 0 | 264 |
| Ac61 | FP protein | [NP_054091.1](http://www.ncbi.nlm.nih.gov/protein/9627804) | 0 | 0 | 645 |
| Ac62 | late expression factor 9 | [NP_054092.1](http://www.ncbi.nlm.nih.gov/protein/9627805) | 0 | 0 | 1551 |
| Ac63 | AcOrf-63 peptide | [NP_054093.1](http://www.ncbi.nlm.nih.gov/protein/9627806) | 2 | 0 | 468 |
| Ac64 | fusolin; spindle body protein | [NP_054094.1](http://www.ncbi.nlm.nih.gov/protein/9627807) | 0 | 0 | 909 |
| Ac65 | DNA-dependant DNA-polymerase | [NP_054095.1](http://www.ncbi.nlm.nih.gov/protein/9627808) | 2 | 0 | 2955 |
| Ac66 | AcOrf-66 peptide | [NP_054096.1](http://www.ncbi.nlm.nih.gov/protein/9627809) | 0 | 0 | 2427 |
| Ac67 | late expression factor 3 | [NP_054097.1](http://www.ncbi.nlm.nih.gov/protein/9627810) | 18 | 0 | 1158 |
| Ac68 | AcOrf-68 peptide | [NP_054098.1](http://www.ncbi.nlm.nih.gov/protein/9627811) | 4 | 0 | 579 |
| Ac69 | putative methyl transferase | [NP_054099.1](http://www.ncbi.nlm.nih.gov/protein/9627812) | 4 | 0 | 789 |
| Ac70 | AcOrf-70 peptide | [NP_054100.1](http://www.ncbi.nlm.nih.gov/protein/9627813) | 4 | 0 | 873 |
| Ac71 | apoptosis inhibitor | [NP_054101.1](http://www.ncbi.nlm.nih.gov/protein/9627814) | 3 | 0 | 750 |
| Ac72 | AcOrf-72 peptide | [NP_054102.1](http://www.ncbi.nlm.nih.gov/protein/9627815) | 0 | 0 | 183 |
| Ac73 | AcOrf-73 peptide | [NP_054103.1](http://www.ncbi.nlm.nih.gov/protein/9627816) | 1 | 0 | 300 |
| Ac74 | AcOrf-74 peptide | [NP_054104.1](http://www.ncbi.nlm.nih.gov/protein/9627817) | 4 | 0 | 798 |
| Ac75 | AcOrf-75 peptide | [NP_054105.1](http://www.ncbi.nlm.nih.gov/protein/9627818) | 4 | 0 | 402 |
| Ac76 | AcOrf-76 peptide | [NP_054106.1](http://www.ncbi.nlm.nih.gov/protein/9627819) | 3 | 0 | 255 |
| Ac77 | very late expression factor 1 | [NP_054107.1](http://www.ncbi.nlm.nih.gov/protein/9627820) | 0 | 0 | 1140 |
| Ac78 | AcOrf-78 peptide | [NP_054108.1](http://www.ncbi.nlm.nih.gov/protein/9627821) | 2 | 0 | 330 |
| Ac79 | AcOrf-79 peptide | [NP_054109.1](http://www.ncbi.nlm.nih.gov/protein/9627822) | 1 | 0 | 315 |
| Ac80 | occlusion-derived virus glycoprotein | [NP_054110.1](http://www.ncbi.nlm.nih.gov/protein/9627823) | 2 | 0 | 1230 |
| Ac81 | AcOrf-81 peptide | [NP_054111.1](http://www.ncbi.nlm.nih.gov/protein/9627824) | 30 | 0 | 702 |
| Ac82 | telokin-like protein-20 | [NP_054112.1](http://www.ncbi.nlm.nih.gov/protein/9627825) | 30 | 0 | 543 |
| Ac83 | viral capsid associated protein | [NP_054113.1](http://www.ncbi.nlm.nih.gov/protein/9627826) | 2 | 0 | 2544 |
| Ac84 | AcOrf-84 peptide | [NP_054114.1](http://www.ncbi.nlm.nih.gov/protein/9627827) | 9 | 0 | 567 |
| Ac85 | AcOrf-85 peptide | [NP_054115.1](http://www.ncbi.nlm.nih.gov/protein/9627828) | 0 | 0 | 162 |
| Ac86 | polynucleotide kinase/ligase | [NP_054116.1](http://www.ncbi.nlm.nih.gov/protein/9627829) | 6 | 0 | 2085 |
| Ac87 | p15 | [NP_054117.1](http://www.ncbi.nlm.nih.gov/protein/9627830) | 6 | 0 | 381 |
| Ac88 | hypothetical protein | [NP_054118.1](http://www.ncbi.nlm.nih.gov/protein/9627831) | 6 | 0 | 795 |
| Ac89 | major viral capsid protein | [NP_054119.1](http://www.ncbi.nlm.nih.gov/protein/9627832) | 4 | 0 | 1044 |
| Ac90 | late expression factor 4 | [NP_054120.1](http://www.ncbi.nlm.nih.gov/protein/9627833) | 4 | 0 | 1395 |
| Ac91 | AcOrf-91 peptide | [NP_054121.1](http://www.ncbi.nlm.nih.gov/protein/9627834) | 4 | 0 | 675 |
| Ac92 | AcOrf-92 peptide | [NP_054122.1](http://www.ncbi.nlm.nih.gov/protein/9627835) | 0 | 0 | 780 |
| Ac93 | AcOrf-93 peptide | [NP_054123.1](http://www.ncbi.nlm.nih.gov/protein/9627836) | 2 | 0 | 486 |
| Ac94 | occlusion-derived virus envelope protein | [NP_054124.1](http://www.ncbi.nlm.nih.gov/protein/9627837) | 2 | 0 | 687 |
| Ac95 | helicase | [NP_054125.1](http://www.ncbi.nlm.nih.gov/protein/9627838) | 9 | 0 | 3666 |
| Ac96 | AcOrf-96 peptide | [NP_054126.1](http://www.ncbi.nlm.nih.gov/protein/9627839) | 0 | 0 | 522 |
| Ac97 | AcOrf-97 peptide | [NP_054127.1](http://www.ncbi.nlm.nih.gov/protein/9627840) | 0 | 0 | 171 |
| Ac98 | hypothetical protein | [NP_054128.1](http://www.ncbi.nlm.nih.gov/protein/9627841) | 0 | 0 | 963 |
| Ac99 | late expression factor 5 | [NP_054129.1](http://www.ncbi.nlm.nih.gov/protein/9627842) | 0 | 0 | 798 |
| Ac100 | basic protein | [NP_054130.1](http://www.ncbi.nlm.nih.gov/protein/9627843) | 39 | 0 | 168 |
| Ac101 | hypothetical protein | [NP_054131.1](http://www.ncbi.nlm.nih.gov/protein/9627844) | 5 | 0 | 1086 |
| Ac102 | AcOrf-102 | [NP_054132.1](http://www.ncbi.nlm.nih.gov/protein/9627845) | 2 | 0 | 369 |
| Ac103 | hypothetical protein | [NP_054133.1](http://www.ncbi.nlm.nih.gov/protein/9627846) | 0 | 0 | 1164 |

Table S4. Continued.

| Gene | Protein | Accession # | # A-read | # I-read | gene length |
| --- | --- | --- | --- | --- | --- |
| Ac104 | viral capsid associated protein | [NP_054134.1](http://www.ncbi.nlm.nih.gov/protein/9627847) | 4 | 0 | 2076 |
| Ac105 | hypothetical protein | [NP_054135.1](http://www.ncbi.nlm.nih.gov/protein/9627848) | 10 | 0 | 1662 |
| Ac106 | AcOrf-106 peptide | [NP_054136.1](http://www.ncbi.nlm.nih.gov/protein/9627849) | 0 | 0 | 186 |
| Ac107 | AcOrf-107 peptide | [NP_054137.1](http://www.ncbi.nlm.nih.gov/protein/9627850) | 1 | 0 | 333 |
| Ac108 | AcOrf-108 peptide | [NP_054138.1](http://www.ncbi.nlm.nih.gov/protein/9627851) | 0 | 0 | 318 |
| Ac109 | AcOrf-109 peptide | [NP_054139.1](http://www.ncbi.nlm.nih.gov/protein/9627852) | 0 | 0 | 1173 |
| Ac110 | AcOrf-110 peptide | [NP_054140.1](http://www.ncbi.nlm.nih.gov/protein/9627853) | 0 | 0 | 171 |
| Ac111 | AcOrf-111 peptide | [NP_054141.1](http://www.ncbi.nlm.nih.gov/protein/9627854) | 2 | 0 | 204 |
| Ac112 | AcOrf-112 peptide | [NP_054142.1](http://www.ncbi.nlm.nih.gov/protein/9627855) | 2 | 0 | 264 |
| Ac113 | AcOrf-113 peptide | [NP_054143.1](http://www.ncbi.nlm.nih.gov/protein/9627856) | 1 | 0 | 510 |
| Ac114 | AcOrf-114 peptide | [NP_054144.1](http://www.ncbi.nlm.nih.gov/protein/9627857) | 2 | 0 | 1275 |
| Ac115 | AcOrf-115 peptide | [NP_054145.1](http://www.ncbi.nlm.nih.gov/protein/9627858) | 0 | 0 | 615 |
| Ac116 | AcOrf-116 peptide | [NP_054146.1](http://www.ncbi.nlm.nih.gov/protein/9627859) | 5 | 0 | 171 |
| Ac117 | AcOrf-117 peptide | [NP_054147.1](http://www.ncbi.nlm.nih.gov/protein/9627860) | 6 | 0 | 288 |
| Ac118 | AcOrf-118 peptide | [NP_054148.1](http://www.ncbi.nlm.nih.gov/protein/9627861) | 7 | 0 | 474 |
| Ac119 | AcOrf-119 peptide | [NP_054149.1](http://www.ncbi.nlm.nih.gov/protein/9627862) | 4 | 0 | 1593 |
| Ac120 | AcOrf-120 peptide | [NP_054150.1](http://www.ncbi.nlm.nih.gov/protein/9627863) | 0 | 0 | 249 |
| Ac121 | AcOrf-121 peptide | [NP_054151.1](http://www.ncbi.nlm.nih.gov/protein/9627864) | 0 | 0 | 177 |
| Ac122 | AcOrf-122 peptide | [NP_054152.1](http://www.ncbi.nlm.nih.gov/protein/9627865) | 4 | 0 | 189 |
| Ac123 | protein kinase | [NP_054153.1](http://www.ncbi.nlm.nih.gov/protein/9627866) | 9 | 0 | 648 |
| Ac124 | AcOrf-124 peptide | [NP_054154.1](http://www.ncbi.nlm.nih.gov/protein/9627867) | 6 | 0 | 744 |
| Ac125 | late expression factor 7 | [NP_054155.1](http://www.ncbi.nlm.nih.gov/protein/9627868) | 5 | 0 | 681 |
| Ac126 | chitinase | [NP_054156.1](http://www.ncbi.nlm.nih.gov/protein/9627869) | 0 | 0 | 126 |
| Ac127 | viral cathepsin-like protein | [NP_054157.1](http://www.ncbi.nlm.nih.gov/protein/9627870) | 0 | 0 | 972 |
| Ac128 | major budded virus envelope glycoprotein | [NP_054158.1](http://www.ncbi.nlm.nih.gov/protein/9627871) | 17 | 0 | 1539 |
| Ac129 | viral capsid protein | [NP_054159.1](http://www.ncbi.nlm.nih.gov/protein/9627872) | 0 | 0 | 597 |
| Ac130 | hypothetical protein | [NP_054160.1](http://www.ncbi.nlm.nih.gov/protein/9627873) | 0 | 0 | 321 |
| Ac131 | major polyhedral calyx protein | [NP_054161.1](http://www.ncbi.nlm.nih.gov/protein/9627874) | 1 | 0 | 759 |
| Ac132 | AcOrf-132 peptide | [NP_054162.1](http://www.ncbi.nlm.nih.gov/protein/9627875) | 1 | 0 | 660 |
| Ac133 | alkaline exonuclease | [NP_054163.1](http://www.ncbi.nlm.nih.gov/protein/9627876) | 14 | 0 | 1260 |
| Ac134 | hypothetical protein | [NP_054164.1](http://www.ncbi.nlm.nih.gov/protein/9627877) | 28 | 0 | 2412 |
| Ac135 | annihilator | [NP_054165.1](http://www.ncbi.nlm.nih.gov/protein/9627878) | 27 | 0 | 900 |
| Ac136 | hypothetical protein | [NP_054166.1](http://www.ncbi.nlm.nih.gov/protein/9627879) | 9 | 0 | 723 |
| Ac137 | fibrous body protein | [NP_054167.1](http://www.ncbi.nlm.nih.gov/protein/9627880) | 5 | 0 | 285 |
| Ac138 | occlusion-derived virus envelope protein | [NP_054168.1](http://www.ncbi.nlm.nih.gov/protein/9627881) | 0 | 0 | 1938 |
| Ac139 | DNA synthesis regulator | [NP_054169.1](http://www.ncbi.nlm.nih.gov/protein/9627882) | 10 | 0 | 1350 |
| Ac140 | AcOrf-140 peptide | [NP_054170.1](http://www.ncbi.nlm.nih.gov/protein/9627883) | 0 | 0 | 183 |
| Ac141 | hypothetical protein | [NP_054172.1](http://www.ncbi.nlm.nih.gov/protein/9627884) | 0 | 0 | 786 |
| Ac142 | early 49 Daa protein | [NP_054173.1](http://www.ncbi.nlm.nih.gov/protein/9627885) | 2 | 0 | 1434 |
| Ac143 | occlusion-derived virus envelope protein | [NP_054174.1](http://www.ncbi.nlm.nih.gov/protein/9627886) | 2 | 0 | 189 |
| Ac144 | occlusion-derived virus envelope/capsid protein | [NP_054175.1](http://www.ncbi.nlm.nih.gov/protein/9627887) | 16 | 0 | 873 |
| Ac145 | AcOrf-145 peptide | [NP_054176.1](http://www.ncbi.nlm.nih.gov/protein/9627888) | 0 | 0 | 234 |
| Ac146 | AcOrf-146 peptide | [NP_054177.1](http://www.ncbi.nlm.nih.gov/protein/9627889) | 0 | 0 | 606 |
| Ac147 | early gene transactivator | [NP_054178.1](http://www.ncbi.nlm.nih.gov/protein/9627890) | 11 | 0 | 1749 |
| Ac148 | occlusion-derived virus envelope protein | [NP_054179.1](http://www.ncbi.nlm.nih.gov/protein/9627891) | 0 | 0 | 1131 |
| Ac149 | AcOrf-149 peptide | [NP_054180.1](http://www.ncbi.nlm.nih.gov/protein/9627892) | 0 | 0 | 324 |
| Ac150 | AcOrf-150 peptide | [NP_054181.1](http://www.ncbi.nlm.nih.gov/protein/9627893) | 0 | 0 | 300 |
| Ac151 | early gene transactivator | [NP_054182.1](http://www.ncbi.nlm.nih.gov/protein/9627894) | 4 | 0 | 1227 |
| Ac152 | AcOrf-152 peptide | [NP_054183.1](http://www.ncbi.nlm.nih.gov/protein/9627895) | 2 | 0 | 152 |
| Ac153 | hypothetical protein | [NP_054184.1](http://www.ncbi.nlm.nih.gov/protein/9627896) | 5 | 0 | 966 |
| Ac154 | AcOrf-154 peptide | [NP_054185.1](http://www.ncbi.nlm.nih.gov/protein/9627897) | 0 | 0 | 246 |
