# Supplementary material for: Analysis of Genes Expression of Spodoptera exigua Larvae upon AcMNPV Infection
Source: PLoS One. 2012 Jul 31;7(7):e42462. doi: 10.1371/journal.pone.0042462 (PMC3409162; doi:10.1371/journal.pone.0042462)
Supplement: Table S5 — Fasta sequence of the 93 AcMNPV unigenes assembled from the RNA-seq. (DOC) [file pone.0042462.s005.doc]

Table S5. Fasta sequence of the 93 AcMNPV unigenes assembled from the RNA-seq.

>Ac-bro length=1049 numreads=15

CACGTGTATCAAAATGGCTCGCGTTAAAATTGGAGAATTTAAATTTGGAGAAGATACGTTCAACCTGCGG

TACGTGCTAGAGCGCGACCAACAGGTCCGGTTCGTGGCCAAGGACGTCGCTAACAGTTTAAAATATACAG

TTTGTGATAAAGCTATACGTGTTCATGTAGACAATAAATACAAATCGTTATTTGAGCAGACCATCCAAAA

TGGGGGCCCTACCTCTAACAGCGTCGTGAAAAGGGGCGACCCGCTGTATTTGCAGCCGCATACAGTGCTC

ATTACTAAATCCGGCGTGATTCAGCTGATTATGAAGTCCAAATTGCCTTACGCCATAGAATTACAAGAAT

GGCTTTTAGAAGAAGTAATTCCTCAAGTGCTCTGCACCGGCAAGTACGATCCCGCGATCAAACAACGGGA

GGAGGAGAGCAAACAGTTGGTGACTAAGCTGATTGCGACATTCACCGAGCACACAAACGCGCTGCAAGCG

GTGGTGGCGCAAAAACCGAGGAACTTGTTAAAAACAAGAGTTTATTGAACGCATCGTTGCCATCAAGGAC

AAGCAGATCGAGGCCAAAGATTTGCAGGTCACGCGCGTCATGACTGATCTAAACCGCATGTACACCGGCT

TCCAGGAAACCATGCAAAAGAAAGACGAAATAATGCAGAAAAAGACGCGCAGGTCACCGATTTAGTTGCC

AAAGTGGTGGATTTGTCGGATCGCGCGGTTCAATACCCCGCGGACAAGCGCAAACATCCGGTGTTGTGCG

TGACGCGCGACGGCACTACGTTTACGGCTATTACCGGCCAAAAGACGTACGTGGAAAACCAAAAGCATAA

ACGTAACATCAACGTTGCCAACATTGTGGTGGAGAATATCCGGCCTAATCCTACCGTCGATTGGAACAAC

GCCACTGATCGGCTACAAGCTAAACGGAGCAAGCGAAGCATTAGTTTTGGTTCGCTGGAAGAAGCGCAAC

AATTTGAAAATAGGATAAAGTATTTGTTAAAGAATGCAAATAATATTAATTAAATTAATAAATCTTGAA

>Ac-ctx length=488 numreads=4

GAGGTGCAGCGAGTCAACGCGGGCGGGGAGTTGTAGGCGTCATAACTATTTATTAAATAAGATAATTTAA

AAAATCGCCGTTAATATGCAAATCAAAACTGTACTATTGGCTTTTGCGATGTTTGCCGCGTTGAACGCGC

AACACGTTTTAGCCGCTTGCGCCGAGACCGGCGCCGTCTGCGTTCATAACGACGAGTGTTGTAGCGGCGC

ATGTTCCCCCATATTTAATTATTGCTTACCACAATAAGTTTAGTTTGCGTTAAAAACAATAGTTGTTGCA

AAATCGACACACGTGTATCAAAATGGCTCGCGTTAAAATTGGAGAATTTAAATTTGGAGAAGATACGTTC

AACCTGCGGTACGTGCTAGAGCGCGACCAACAGGTCCGGTTCGTGGCCAAGGACGTCGCTAACAGTTTAA

AATATACAGTTTGTGATAAAGCTATACGTGTTCATGTAGACAATAAATACAAATCGTTATTTGAGCAG

>AcOrf-4 length=529 numreads=4

AGATGCGACAACGGGCGCATTGTTAATTCTTGACGTGCGATACATAATATATTATTGGATAAAGTTGCAT

TAAATGAAACTAACTTACAAGATGGCTAGTTTGTTAAAATACGCGCTGCGCTTGACTCGGGAATACAAAG

AAAACATTATTCCACACTTTGATCACTTGACTCGATTGCGCGATTTAATCGACGGCATGATTAAAAGCGA

GGATGTACAACGTTTTAATCGCACTAATCGCAATGATTTAATTTCGGCTTGCATGCAAATCAACGTTCGG

ACGTACATGCCCAACGCCACGATAGATATGCGCAAACAACCCAACTGTATATATTTTCGAATTTGCCAAT

ATTGCCACTTGGAGGCCGACGTGCCTTCGCCCGACGATCATTCGGTGTACAGATACTTGTGCGTCGCGTG

CGGCACGCCGCTGGTCATCGACCACCCGCTCGACGTGTTCGGCCACACGGAGGAAGGCGTCAACGAACTG

CTCGAGGTGCAGCGAGTCAACGCGGGCGGGGAGTTGTAG

>AcOrf-5 length=171 numreads=1

TATCCAATAATATATTATGTATCGCACGTCAAGAATTAACAATGCGCCCGTTGTCGCATCTCAACACGAC

TATGATAGAGATCAAATAAAGCGCGAATTAAATAGCTTGCGACGCAACGTGCACGATCTGTGCACGCGTT

CCGGCACGAGCTTTGATTGTAATAAGTTTTT

>Ac-lef2 length=671 numreads=8

GCGAGAAGCCGCGAAGTATGGCGAATGCATCGTATAACGTGTGGAGTCCGCTCATTAGAGCGTCATGTTT

AGACAAGAAAGCTACATATTTAATTGATCCCGATGATTTTATTGATAAATTGACCCTAACTCCATACACG

GTATTCTACAATGGCGGGGTTTTGGTCAAAATTTCCGGACTGCGATTGTACATGCTGTTAACGGCTCCGC

CCACTATTAATGAAATTAAAAATTCCAATTTTAAAAAACGCAGCAAGAGAAACATTTGTATGAAAGAATG

CGTAGAAGGAAAGAAAAATGTCGTCGACATGCTGAACAACAAGATTAATATGCCTCCGTGTATAAAAAAT

ATTGAACGATTTGAAAGAAAACAATGTACCGCGCGGCGGTATGTACAGGAAGAGGTTTATACTAAACTGT

TACATTGCAAACGTGGTTTCGTGTGCCAAGTGTGAAAACCGATGTTTAATCAAGGCTCTGACGCATTTCT

ACAACCACGACTCCAAGTGTGTGGGTGAAGTCATGCATCTTTTAATCAAATCCCAAGATGTGTATAAACC

ACCAAACTGCCAAAAAATGAAAACTGTCGACAAGCTCTGTCCGTTTGCTGGCAACTGCAAGGGTCTCAAT

CCTATTTGTAATTATTGAATAATAAAACAATTATAAATGTC

>Ac-PH length=339 numreads=1

ATTAGGATCGTCGAGCCTTCATGGGTGGGCAGCAACAACGAGTACCGCATCAGCCTGGCTAAGAAGGCGG

CGGCTGCCCAATAATGAACCTTCACTCTGAGTACACCAACTCGTTCGAACAGTTCATCGATCGTGTCATC

TGGGAGAACTTCTACAAGCCCATCGTTTACATCGGTACCGACTCTGCTGAAGAGGAGGAAATTCTCCTTG

AAGTTTCCCTGGTGTTCAAAGTAAAGGAGTTTGCACCAGACGCACCTCTGTTCACTGGTCCGGCGTATTA

AAACACGATACATTGTTATTAGTACATTTATTAAGCGCTAGATTCTGTGCGTTGTTGAT

>Ac-ORF1629 length=445 numreads=1

CGAGAGATACGACGTTAAATTGTGCGCCGAAATTGTTTTCTGCAAATACAATTTGCTAGTGGGCTTGTGC

GATAAAATAGATATTTTGCCAAAACGCCCGTTGATTATTTTATACCGAGTTTTGACGTTTTTGCAGTTTT

CGTAAAATTGTACCAGCGTTTGCAGCGTAGCATTTGTGGTGGTGGTGGCCATGACGAATCGTAAATATGA

ATCTGTACAATCTTATTTATTCAATAATAGGAACAATAAAATTGATGCGCATCAATTTTTTGTAACACGT

CGATACTGCGGAAGCGCAAATTATCAAAGACAACATTTACGACAACACAGTGTTGCTGAACAGAGATGTT

TTTTTAAATATTTTAAAGTTTGCCAACGACGTTTTTGACAACAAAGCGTACATGTACGTCGACGACAGCG

AAGTGTCTCGCTACTACAATGCTGT

>Ac-ORF1629 length=210 numreads=2

ATCGACAAAGCTAGATTGCTGCTACAAGATTTGGCAAGTTTTGTGGCGTTGAGCGAAATCCATTAGATAG

TCCAGCCATCGGTTCGGAAAACAACCCTTGTTTGAACTAATCGAAACCTATTTTACAAATCTATTGAGGA

TTTAATATTTAAATTCAGATATAAAGACGCTGAAAATCATTTGATTTTCGCTCTAACATACCACCCTAAG

>Ac-pk-1 length=409 numreads=2

CGATTCGTCATGGCCACCACAAATGCTACGCTGCAAACGCTGGTACAATTTTACGAAAACTGCAAAAACG

TCAAAACTCGGTATAAAATAATCAACGGGCGCTTTGGCAAAATATCTATTTTATCGCACAAGCCCACTAG

CAAATTGTATTTGCAGAAAACAATTTCGGCGCACAATTTTAACGCTGACGAAATAAAAGTTCACCAGTTA

ATGAGCGACCACCCAAATTTTATAAAAATCTATTTTAATCACGGTTCCATCAACAACCAAGTGATCGTGA

TGGACTACATTGACTGTCCCGATTTATTTGAAACACTACAAATTAAAGGCGAGCTTTCGTACCAACTTGT

TAGCAATATTATTAGACAGCTGTGTGAAGCGCTCAACGATTTGCACAAGCACAATTTCA

>AcOrf-11 length=1007 numreads=13

GTCTCTCGCTGCAAAAGTAAATAAATTACAATTATTATGCTAAATACAATGAGGTGCACGACGTGTATGG

CGAGTCGTATCACCATTACCGTATAGTTCAGGAATACCTATCAGAATCGTACGTAAACGGTATGTCGTGC

ATAGAGCGGGACGTGACCGCTATGCGGCGTTTGAAGAGCGGCAGTTGCACCTTTGACGAAGCGGTCAAGA

TGATAGACGCGGGCGATTCCATCAAGAGCCTTTCCCATTGGTTCAGCACCAGCGAAACGATGGGCATCGA

CGATAACGTCCGTGAAGTGTTGGAGCAAATCGATGCGGTGGTTCCCGTCAGCGTGCGCGTTCAAAACGGA

TGGCAGATATTCTCTTTGAACAATTTTGAACGAGAAATTTCTCAAGACATGTTGGATTGCTTGCAAATTA

TTTTGGGCCGCTTTGAATATTTCATGAGAAACGGGAAACTGCTGCGCATCGCCAACGTGTTCAACCCTAA

TAACGACGTTGTCGGATGGTGGTACAATAAATTTTGTGTGGTAACTTATGTGCACAGAATAATGTATCGA

AGCGTGCCCGCCGAATTGGTGCCTCGTTTGTCGGAAGCGGTGAAAAAATTTATTCGTTTGCGCAAGAGTG

ATTATGATGATCGTTTACACGTAGACGAATCGTACAATTGTCCGCGTGTAATCGCCGAAATGTATGGCCG

GTTTTGCGGTATAGGCAAGGAGCATTTTAGTAAACACAAGTTGTCGTGCATGCACATTTTATTTCAATAT

CTGCGCGGCAAAACCACGCAAGAAGAAAAGTCTTTTCCTTGTTATAGGGTTATTAAAGATTTTGGTAGAC

AATGTAAAGATGTGTATAAAAATTTAAAAGATGTATTTGATTTGTTACATGCACATAGTATGTCGGATAA

AGACAAAAACTCATTGATGGACCTGTTGTGTGTTATGGATTGTGAAGAAATAGATGTAGATTGTTTTTAT

TATATATTTGAATCGTTTTTAAATAAT

>AcOrf-12 length=369 numreads=2

GTCATTGTAAATTAAATTCTCTGCCTCAAGAATTATTTGACAAGATTGTCGAGTATTTATCTTTATCTGA

TTACTGCAATTTGGTGCTTGTCTGTAAAAGACCTTCTAGTAAATATAACGTGATATTTGATAGTACTAAT

CACCAACATTTGAAAGGCGTGTACAAAAAGACAGACGTGCAAATAACAAGCTACAACGAATACATCAACT

GTATTTGCAACGAACTGAGACAAGACGAATTCTATGCCAAATCATCATGGATTGCGAGTATTTGCGGTCA

CCAGAGAGCGACAATTTTTAGTGTAACAAATAAACAAGTAGAAATGAAATATCATTTGTATAATATAGCA

ATTGTGGAAAGTGAAGATT

>AcOrf-13 length=920 numreads=13

ACAGCGACACGAACGCAAAATGCTACAATGTGATAGATTTTTGCAAAGGTCTTGAAATTGCGCACGACGA

CATACTTGATTGCAATTGGGACGGCGATCAAGTTTACCATTTAAACGAAATTGTTTTTCACAAACAGAGA

TCCAAACGCGATCTCAACTCGTTGGGCGCATTGTTCGCGACCAAGCACGGGTTGTTGGAAATTTTGATGC

GGTTAAATTTTGCCAACAAAAGCAACGCGTTGCTGCACATTCAAACTGAAGGCGAGCGTGATGATTTGCG

CGACAAGATTGAATCTGTTTTAAAAACATGTAAAGAAACTGAATGCAAACAGCGAAAAATTTATGGTCAC

CCACGAGACGTTCAAGAACGAGGTGGGCAACAGGTTTGAGCAGTTTGAATTGCGTTTGCACGAACTCGAC

GCCAAACTTAACATGCTGCAGTCGGCCGAAAAATTGAAAACCGCCGTCGTGGCGGAAAGCAAAAATGGCA

CGGTGACGTTTCCGCGCGACATCACCAAACACCAGCATTTGGCCGTATTTTCGGAACGCATCGACGACCG

CATCAAACTCGCTTTTGTTTTGGGCCAAGAGCGGCATTTTCGCAAGCGAAAAATGCGCTTTGAAGACGAC

ATGGAAGTACTCTATGACGGTGTGCACCCAAATCCCTTGTTGGCTATTCAATGTATTAACGAAAAACTCT

ACGATAAACATTACAAAATTAGAAAAATAGCTAAACGTGTAATTGACGTGGATTGTACTCCTAATGTAGT

TAGAGAGGTTATACAGGAAGTATTGTAAAATAAAAACATGTATAATAGTTATTATTATGTATTTATTTCG

TATAAATAATATATAAAGTTTGATTGTACATTTTGTTTTTCTAAATATTTACACAACGAAACAATAAATG

AATTACGAGG

>Ac-lef1 length=204 numreads=1

GGAATGCTAGACAAGCTAAAGCAATGCTATCCTGGTTATGGAACTGGTGGATGTGGTCCGGTGACAACGA

CAACGACGCCATCGCCGCCGAAGATCGGTTCAATGCAGACGACTACAAAAAGTACCACATAAACGCCCAA

CAATGGTCGCACATCGTTAAATGGGACTCATTCAAATGCAACACGCACAGTTTCAAGTACAGAT

>Ac-egt length=1112 numreads=16

GTGTTAATTTTTGGCTATAAAAAGGTCACCCTTTAAAAATTTGTTACATAATCAAATTACCAGTACAGTT

ATTCGGTTTGAAGCAAAATGACTATTCTCTGCTGGCTTGCACTGCTGTCTACGCTTACTGCTGTAAATGC

GGCCAATATATTGGCCGTGTTTCCTACGCCAGCTTACAGCCACCATATAGTGTACAAAGTGTATATTGAA

GCCCTTGCCGAAAAATGTCACAACGTTACGGTCGTCAAGCCCAAACTGTTTGCGTATTCAACTAAAACTT

ATTGCGGTAATATCACGGAAATTAATGCCGACATGTCTGTTGAGCAATACAAAAAACTAGTGGCGAATTC

GGCAATGTTTAGAAAGCGCGGAGTGGTGTCCGATACAGACACGGTAACCGCCGCTAACTACCTAGGCTTG

ATTGAAATGTTCAAAGACCAGTTTGACAATATCAACGTGCGCAATCTCATTGCCAACAACCAGACGTTTG

ATTTAGTCGTCGTGGAAGCGTTTGCCGATTATGCGTTGGTGTTTGGTCACTTGTACGATCCGGCGCCCGT

AATTCAAATCGCGCCTGGCTACGGTTTGGCGGAAAACTTTGACACGGTCGGCGCCGTGGCGCGGCACCCC

GTCCACCATCCTAACATTTGGCGCAGCAATTTCGACGACACGGAGGCAAACGTGATGACGGAAATGCGTT

TGTATAAAGAATTTAAAATTTTGGCCAACATGTCCAACGCGTTGCTCAAACAACAGTTTGGACCCAACAC

ACCGACAATTGAAAAACTACGCAACAAGGTGCAATTGCTTTTGCTAAACCTGCATCCCATATTTGACAAC

AACCGACCCGTGCCGCCCAGCGTGCAGTATCTTGGCGGAGGAATCCATCTTGTAAAGAGCGCGCCGTTGA

CCAAATTAAGTCCGGTCATCAACGCGCAAATGAACAAGTCAAAAAGCGGAACGATTTACGTAAGTTTTGG

GTCGAGCATTGACACCAAATCGTTTGCAAACGAGTTTCTTTACATGTTAATCAATACGTTCAAAACGTTG

GATAATTACACCATATTATGGAAAATTGACGAAGAAGTAGTAAAAAACATAACGTTGCCCGC

>Ac-egt length=465 numreads=4

AAAAATGGCGGCGTTTATTACGCAAGGCGGACTACAATCGAGCGACGAGGCCTTGGAAGCCGGGATACCC

ATGGTGTGTCTGCCCATGATGGGCGACCAGTTTTACCATGCGCACAAATTACAGCAACTCGGCGTAGCCC

GCGCCTTGGACACTGTTACCGTTTCCAGCGATCAACTACTAGTGGCGATAAACGACGTGTTGTTTAACGC

GCCTACCTACAAAAACACATGGCCGAGTTATATGCGCTCATCAATCATGATAAAGCAACGTTTCCGCCTC

TAGATAAAGCCATCAAATTCACAGAACGCGTAATTCGATATAGACATGACATCAGTCGTCAATTGTATTC

ATTAAAAACAACAGCTGCCAATGTACCGTATTCAAATTACTACATGTATAAATCTGTGTTTTCTATTGTA

ATGAATCACTTAACACACTTTTAATTACGTCAATAAATGTTATTC

>AcOrf-16 length=396 numreads=2

GAGAGGGGCTTTGTGCGACTGCGCACTTCCAGCCTTTATAAACGCTCACCAACCAAAGCAGGTCATTATT

GTGCCAGGACGTTCAAAGGCGAAACATCGAAATGGAGTCTGTTCAAACGCGCTTATGTGCCAGTAGCAAT

CAATTTGCTCCGTTCAAAAAGCGCCAGCTTGCCGTGCCGGTCGGTTCTGTGAACAGTTTGACACACACCA

TCACCTCCACCACCGTCACCAGCGTGATTACCAAAAATTATCAAGAAAAACGTCAGAAAATATGCCACAT

AATATCTTCGTTGCGTAACACGCACTTGAATTTCAATAAGATACAGTCTGTACATAAAAAGAAACTGCGG

CATTTGCAAAATTTGCTAAGAAAAAGAACGAAATTATTGCCGAGTT

>AcOrf-16 length=373 numreads=2

CTTGGAGTAGTCAGATGTGACAACACAATTCGCACAATTATTGGCAACGAAAGTTTGTAAGGAGACGTTT

GGCCGAGCTGTGCACATTGTACAACGCCGAGTACGTGTTTTGCCAAGCACGCGCCGATGGAGACAAAGAT

CGACAGGCACTAGCGAGTCTGCTGACGGCGGCGTTTGGTTCGCGAGTCATAGTTTATGAAAATAGTCGCC

GGTTCGAGTTTATAAATCCGGACGAGATTGCTAGTGGTAAACGTTTAATAATTAAACATTTGCAAGATGA

ATCTCAAAGTGATATTAACGCCTATTAATTTGAAAGGTGAGGAAGAGCCCAATTGCGTTGAGCGCATTAC

CATAATGCCATGTATTTTAATAG

>AcOrf-17 length=322 numreads=5

ACGATGAAGGTACAACAATGCCTTATGTCATTGGACCATTATATTCTGTCGACGCTGCTGTCGCCGACCG

TAAAGTGAAGGACGTGGTGGATTCAATTCAAAACCAACAGACAATGTTAAAAGTATTTATTAACGAGGCT

AATGTGTATAACAAATGGAATATGCTTAAAGGTTTAATTTATAATAATAACAATGAAATCTGTTTTAGTA

AATAATGTAGTAAAATTTATAAAGGTAGATAAAAATTATAATATTAATAAAAAAATAATGTTACTAAATG

GGTTCCTGCGTTAAATATTTTACGGGTAGACAGCTATTAACA

>AcOrf-18 length=445 numreads=2

CGCATTTGAACAGTGTAGTACGTAGTTAGCGTACGCCGCTATTCATTATGGAACGTTTGTTGAATCAACT

AAATCTTGGCGTCCTACCTTATATTACGACAAAGGATATTGAAGATCGCCTGCGCGATAAAATTGTGGCT

AAAGCAAAGTTGGCATTTATCAAAGATTGTTTCGAAGCCGTAGTTTGCGAAAACGGCGGTTTATTCGTGT

TAACTGGAGGCGCAGCCGTGACATGCCATATTGACGACGACAGAAGCGCATTAAAATGCATAGATTTTGA

CTATTACGGCTTTTGCGCAAAAATGTTTTGCAACCTGCAAACTAATTTACAAAAATGCGTCGACCAACAT

TACGCTGAATTGGACGTGCTAACGCGTCAAGTTTACATGTCGGATCCGTTGGTAGTGCTGAAATGTTATC

AAAAACGGAGCCTATAGATTAAACG

>AcOrf-20 length=384 numreads=1

AGAAAGTTTGTGTACCTCACCGGACGCGTGCACAATGTGCGAGCCTCCGTTGCCGGATGAAGAAATCATT

AGTCGGCGCACGCCGACGCTTTCGCCCAAGCTGTTCCGCAAGTCCAGAGAACTGTCGCCCATCAAACCCG

TAAGGACTCCTACTCCACCCGCTCCCACTCCACCGCCCATGTGTTTTAGCGAAGAGTTGCAGCGTAAATT

TCAAGAGAAAAACTATTACCCGTTTATGATGATTAGAAAGTATTATATAAAGTATTATTTTTGATTATAT

AAAGTATTAATTTAGTATTAGCGTAATTTAGTATTAATTTAGTACTAATGTAAATAAAGTATTATAGTGT

AAATAAAGTATTGTAAATAATTGTATTAATAAAT

>Ac-env-prot length=636 numreads=2

GTTATTTCGATTTGTCGCAAGCGTATAAATTGCATTTATATGTTGATTTTAAACACGGTCATTATGTTTA

TTACCATGCCATTGTTAAAATCCACCGCCGTTTCGTTTAATTTGTATCGCGTCATGACGGTGCCTTTTTG

CAGGGGCAAAATGTGTCTGCTTATCATTTCGGGCAATGAATACTTTGGGATTACAGACAGCAAAAACTAT

TATGTGCCCGTATCTGATAACTTTAAACAAGATTGCCAAGAGTTTACGGGCTACAATGAGTTTTTGTGTC

CCGAAACTGAGCCGATTGCCACTATGAACTCGAAAGTGTGCGAGATTGAAATGTTTATGGGTCGATATAG

CGACGACGTGGACAACATGTGCGACATTAGGGTGGCCAATTATAATCCCAAAAAAGCTTACGTGAACACT

TTAATAGACTACCGAAAATGGTTGTACATTTTCCAAACACGACCGTGTCCGTCCACTATTATTGTCACGA

CGCGCTTGTAGAAGTTGATACAAAGTTTCGCCCGGCGTTGGTGTTATGTTTTCGACTATGGCGCAAACGT

GTTCGATTAGAATAACGTATGATGTGACCATAACTGTAGATTCGCGATTTTATGTCAGCCATTTCAACTA

CATACT

>Ac-env-prot length=353 numreads=2

ACGACAGTAATACCACCGTGGTAATTATCGCTATTGTCGCTGCAATGATCCTATTCTGTGGATTATTGTT

ATTTTTGTTTTGCTGTATAAAAAACGGTGTCATCAATCAAATAACGTGGTTGTGCAATACAAAAATAACA

ATGAATTTGTCACAATTTGCAATAATTTAGAAGACAATCGAGCATACATTAATTTACCTAATGAATACGA

TAGCGATGATATGCCAAAACCATTGTACCCTTTACTTGGCTTTAATGATGATTTGTTAAAAGATGATAAA

CCTGTGTTGTACCCTATGATTATAGAAAGAATAAAATAAAACATGTATAATTGAAATAAATATATTATTT

AAT

>Ac-pkip length=507 numreads=1

GCGCTTTGTAAAGAAAAGGTACAAAGCGAAATTTAAATAGTTTGATTAAATTGCAAAATAAAAAAGTTAA

AAATTATTATGTTAAAAACAATGAAACTGCAATCGACAAAATGTTGTGCATAGCGGCCGATATCAAAGGC

CAAGTGGAGCAGCTCGAACTCGTAAACCAGTATCTGGGCGCGCCCGAAAGTGAAAAGCTAGATTTTGTAT

ACGATTGTTCCGACTTGGATATAAATGAAAAAGATTTTAAAATCTCTGTGTTTTGACTAAAAATATTGCG

TACTTTTACCCAAAAATACAATGCGCCGACCGTGCTGAAGGCCCAGGCAGCGGTTTACGATTCGTTCATA

AAACACAGCGAATTATTTATAAACGCCATATGTCAAATGGATGAAAAACAGCAAGTGAATAATTTTTGTT

TAGACGAATTAGTAAAATTAAAACTGATAGCCATTAAACATTTGTGTAGCATTGGAATATGTAATAGAAA

ATAGTATATAAATAAAA

>AcOrf-25 length=1079 numreads=20

GCGTTGTCTACGCACATGCTGGCAACAGAGTCGTCCATATTTATTATATATCTTATATTCTGTGAAACAC

TTCAATTAGACTTGAACCACAGCAGACAGCGCACGTCGGTAGCATGGCAACTAAACGCAAGATTGGCGAT

GGATATTCGAGCAGCGACGACAACCAGCCGAAGCGGGAAAGATCGGAAGGTGGTGAGGACCAGCAGCTGG

TGCCGTACAACAGCGGCGCTTTTAACGTGAAACACGACGAAACGGGCGTAATGTGCTATTTTACGCCGTC

GTCCATTCAATTGGAGCCACACGAGTTGACGAAGATGTTGTGGCAGGAGCAGATGGCGATTAATGTGAAA

CGAGGCAACTTTTCAATACTGAATTGTAGTTGTTTCGAAGGCAGATTTTTGAAAAACGAATTTTGTCGAC

TGTCTAATTTGAACAGTTTGCACGAATGGGAAGACAAGTTGTATCCCGAGCCCGATAAAAATATCGTGGT

GTTGGAGCCGGCCAACGGCAAGACTACGTACACCATCGGTCCAAGAGTGCAGGGCAAGCCCTGCGGATTT

TGGTTTTCCGACTTTGGAACCATAAAACGTGCCAAAAGCAACTTTGGCCAGTTTTTTAGCATTCAATACG

GAGACATTCACAAACACAACAATATATTTGGCAACATTTTGCAAAAACACTTGCAAAGCGATTTTCCTTT

GAAGATGGAACCAAACGTGTGCATTCATTTGCCGGATAAGAACAAAACTAGCGAGCGCGACATGTTGATT

CGTCGGTTTTACATAATCAACCGTGACAACAACGGATCAATTTACGCCACCGGAAAGATCAGAAACGTTC

CTCTTGATATACAGAGAATGAGCGTTGAAGATTTTGATAGATTGTTCGAAATGGACAAAATAGACGGACC

TTCGGAGGAGATTAAAATGTACATGATGGGCACCATTGACGGCGTCAAGTACGGCAAAGAGATGCAGATG

ACGGACATGAATAATAAGAAAATTACCGAAAAGCCCTATTCGTTAGCTTTTAAGCCTGGAATATTTGTTA

TTATTGAACAATAAATGAATATATTAAAA

>Ac-lef6 length=489 numreads=4

CCTGATTTGAAAACAGCGTCGACTGGAACGGCAGCACGCGCAAACAGCTGCGCGTTCTAGACAAGCGCGC

CTACAGGCAGGTGTTGCACTGCAACGGCAGATACTACTGGCCCGATGGCACAAAGTTTGTCTCTCATCCG

TACAACAAATCTATTCGCACGCACAGCGCAACAGTCAAACGGACCGACAGCTCGCATCGATTAAAAAGCC

ACGTGGTCGACAAACGACCGCGCCGCTCTTTAGATTCTCCTCGCTTGGACGGATATGTTTTGGCATCGTC

GCCCATACCACACAGCGACTGGAATGAAGAACTAAAGCTGTACGCCCAGAGCCACGGCTACGACGACTAC

GACGACAATTTAGAAGATGGCGAAATCGACGAACGTGACTCTTTAAAAAGTTTAAATAATCATCTAGACG

ACTTGAATGTATTAGAAAAACAATAAAACATGTATTAAAAATAATAATAATAAAACTATATTTTGTAAT

>AcOrf-29 length=384 numreads=1

CAAAATGTCAATGCCAACACTTATCGCAATAAAAATAACCATTTTATGGAAGATGTGTACGAGCAAATGG

TCGCGATACAAATGAATAATAATTAACTCCTAATTTGGATAAAAGATGCTGTTTTACCTCATCTGTATGT

TTTCTCAAGATGTTCTCAAGGAATTACAACGCATCTCAATCTCAGCGAGATTTAAAATCTCAATTGGAAG

AAATTAATAGACAAAAGCAAAAATAACTATTGACAGCCAGCATTTTGAGAAAATTAAAAGCGTCACAAAA

ACGTAAACGAGCTGCAGAATATGGAGAAGAGAGTTATGAAGTCAAGACAAAACTTTCTCAACTACGGAAT

AGACAATTTTTAAATAAAATACATTATATATTAC

>AcOrf-30 length=498 numreads=2

GCAAAAAGAATATATAAAATAATTGAATTGGCCGTCAAATCTCCCAACAATCGTGTCTATTTGCTTAATT

TGCAAGATGACACGTTTTGGAAGCGCATATCGCGCGAATGTTACGGACGCGTCGATTTTATTCACGTGTT

TCGCAACAAGTTGGATTGGAAAATTATTTCAATTTCGCCGTTGTCGATAACAATCGCCAACAGGTTTAAA

TCACATTTGATCTGGTCGTTGGTGTCTGAGCAAAAATTTTTGACGCAAGATTTTATACTCGCGTTTGGGG

ACTTGTTGGACATGGAAGAAATATCCAAAAATTACAATAACCTCTCGTTGTCGGTGCAGCAAAAATACGC

ACACAAATTAAATTGGAAACGCATAGTGGCCAGTCACATACTACTAAAAGAATGGTTTCAAGAACCCATT

AAACAGTACATCAACTACGATTATGTGTCCAAATACAAACACTTGAACACGGCGCTAATAAACAATGTGA

GCTGTATG

>AcOrf-30 length=142 numreads=1

CTCATTTTGTATTGCTTACGCGAAGGCCGCGTGCAAGAACTAAAATTGATCGCCCACAGCATACCGTGGT

CGGATCACATGCTCGTGTTTGACGAATATCCCGGTTTGGTGAACACGTTACATTCGGACTGGAAGTGTAT

TG

>Ac-fgf length=554 numreads=4

GCTGTGGGGAATTAAAGTTCATCATGTATCGCTTGCTGGCACTTGTAGCTCTAGCCTCTATGGCTGACTG

CTCAGCTCTGCTGACACACATCACGGGCACGTCTATTCCCGGCCAGCTGTTTATTAACCGGCAGTTTTTG

GCAGTAAATCCAGACGGAGCTGTTTACGGAACCATTGAATCGGACAACGTCGACACCATATTCAAGCGCG

TTGCCGTTGACAGAAATCGCATCGTCATTCAAAACGCCATCACGTGTGTGTACCTGTGCATGGACCGGTG

TGGCCAACTGTACGGATCAAAAACTTTATCCAAGGATTGCTTTATGAGGGAATTTCTAGGAAAAAAATAA

CTACAACACGTATTATAAAGTGTACGATCGCAAGTTGACGTATGTGGCACTGAAGAACGACGGTACTCCC

CGAAAACTACAGATTTCAAAGAGCCGCAAGCTGGGCAAGCTTAGCGTGTACGCCATGGCTTTATGAAACG

CTTAAGCTTTCCTATATACACGTCGTGTCCTAATATAAAAAGTGAAATTATTGTTCGCCATCGT

>Ac-39K/pp31 length=941 numreads=15

GCGTAAGGTGTGTCTTCATCACAAACGAATTGCCAGGTTGTTGGGCATTAAAAAAATATATCATCAAGAA

TACAAACGGGTTGTTTCAAAGGTTTACAAGAAGCAAACATGGTAAACGTGCCGGAGCAACAATCTCCGGA

GACTGCGGCCGTGTGCAAAAATGAAAAGCTGTTGAATAAACTGGAATCGAGCTCTTACAACAAATCCAAC

ATGGACCAGCTGGCCGTTATTGTAAATTTCTTGGAAAGAAAGAACATTAACTATATCCTTAACGTGGTGC

CTGTCATGCAAGACGAACGCAAAATGTCCAAACGCAAGAAGAAAGTGATTAACAACAATAAATACATTTT

GTTTAACAGTTGGTACACTAAGATCAAGCAGCCCGAGTGGCCTAGTAGCCCGGCCATGTGGGATTTGGTG

AAAAACAAGCCCGAATTGGCAGATTTTGTGTTCATTTTTGATCACACTGAAAAGCTGGGCAAAAAAATGG

CCGACCGATCGACATCGTCGTCTTCAAGCGAGAACGCAGCAATTCCAGCGAGCAAAAAAAGACAGACTGT

GGTGCTTACCAACGCAAATTTGGCAGAATTGAAGGAGAGCTGCGAAATGCGCGATAAGCTTTATTCGGAG

TTTTACAGCTTGTTAAACGAGACATTTAATCACAATGTTGCGCCTCTGCTAAGTAACATTTACGACGAAG

TGTTAACGCGCGATTTCATCACTAAAAGTATGGCCAAATTTAAGACGGTAGCGCTCAAGTTGCCCGTTGC

ACCGTCTACTACAGAGTACGTGCCCACTCCCATTAGCGGCAGCAGGAAAAGGAAAAGCTCAGTTCCAGCC

AAACAACGATCTTCTATAAAGACTCGCCGCAACACTGTGGCTCCAGCACTTTTAATGGTTAGCGATAATA

CACAAGATACAAATATGTCAGATTAAATAAA

>AcOrf-38 length=371 numreads=8

TGTTGCCGGGCACGTTTGGCAATGACTACCGTATAATGTTAAAACCGCGACGCTTCAATTGCGAAATAGC

GCGCAGCCTGGCCATCGTGCCGCTCAACAAATACTTTAATTATATGAACGACAAACAACTGATCACGTAC

GATTACAGCAATTACATTGAATTTTTTGATTTTGTGCGCAGCGTCAAGGCGCGTTTTGATAATAGGCAAT

TGCAGGACTTTTTCTACGCCACTCTGAAAAAGATAGACAACGATGCCCCCCAAAAACTTAGCACGCACTT

AGGCGGGTGTGATTCGGACTGCTTGACTCGCAGCGAAATACAAGCGCTGTTCAGGGAAGGCCATCAACAC

GCTCAAGCACACGATGAACAC

>Ac-p43 length=415 numreads=2

TGGAATGGAACGCCAACCGCTTGTTTTGACGAGAGTAACTAAAATTTTGCATGTTTACATTGGGCAATAT

TATACAAGCAGTTGTCGCCGCGTAGAAAAAAGTTTTTTCAAATACAACGGGTGGAACAGACAAGGGCGGT

TTTGCAGAACCGAAAAATTTTGTCATTTAGTCAACTTGCAACTGAACAAGGACGGATCTAAAAAATTAAA

ACGGGTTCAACGTAAACTGATAAATTGTAAATAGTTGCAATTAGGTATCAAACGATGGTGCTCGTGTGCG

CAATTTTTTAAGTGTCAAATATGTAAAATGCGAAACGCTGCAGGATTGTTTATGATAATCGAGCCGGACA

AGGCTGTCCTGCTGTGTGCCCGTCGCGCGTACCGGAGCGCGAACGCGCCCGCCGCCGACATGAAC

>AcOrf-47 length=376 numreads=6

ACTAAATGTTTACATCAATGAAACGAGCTAACGTGAAAGTAAACATAAACGTTTAACGATATATATGCAA

CAACCTTTTAATATTCACATTAAAAAGTGCCAGGATGATGGAGCGCACAGTCACTCGCTGGCATTTGGTG

TCGGATAATGTATTATTTAAAATTGGCGAGGTCGCCCAACGACTAGATTATTATTTGCAAGAATACGCAA

ACTTGGAAATGCAAATCGAGGAAGAAATCAAATATATGGAAGTGGATGACGGCGAAGAAATCGACACTAT

CAAGACTTTTCTGCGCAATTCCATGTCCACGTCGGAACAGCGTGATTTGTACGCGTTAGCATTAAAATTA

AACAGTTTAATAAATAAATAATATTT

>AcOrf-48 length=429 numreads=4

AGGAACTGCCGTTCAAATTGGAATATTGCATTAAAGACGTGGGCGTGTTGGCGTGCTTTTTAGCGCCCAA

GATTGTTAACAACGACGAGGAAATTTTTTAATTGCGCCGCATTTGCAGTACGAAATGGATGCGCTGCATG

GAATTTGCGTCAACATTAATAAATTTTATAAATGCCGCAGAATTGTAATAGAATACGACAATTGTAGCGC

TTCGTTTACTTTTAATTGCGTACACGATAATAGAAAAGTGAATTGTTTAGAAATAGTTGGCTTGAGAAGA

AACGAGTACGTGTGTTTGGGCAAAATAATAAACGGTGATAAAATTATATCTGTGCACGAAAACAGTGTCA

ATGGGAAAATAATCGTACCTGTCGAAGACACCTTTGATTTTGGTCTGTTTACTTTAAAAAATAAAATTAC

CGACGCTGT

>Ac-pcna length=638 numreads=4

AGAATCACTGCTAAAAATGTTCGAAGCGGAATTTAAACCGGCGCAGTTTTGAAGAGGCTGGTGGAAACTT

TTAAGGATCTGCTGCCGCACGCTACTTTTGATTGTGATAATCGAGGCGTAAGCATGCAGGTTATGGACAC

CTCGCATGTCGCGCTCGTGTCGTTGCAACTACACGCCGAGGGTTTCAAAAAATATAGATGTGACCGCAAC

GTGACGCTCAACGTGTCCATAAACAGTTTAAGTAAAATTGTCAAATGCGTTAACGAGCGCAGCTCTGTAC

TGATGAAAGCGGAAGATCAGGGCGATGTGATGGCGTTCGTGTTCAACAACGACAATAGAATCTGCACGTA

CACGCTCAAGTTGATGTGCATCGACGTGGAACATTTGGGAATTCCGGATAGCGATTACGATTGCGTCGTC

CACATGTCGAGCGTCGAGTTTGCGCAGGTGTGCAAAGACATGACGCAATTCGACCACGATATTATTGTGT

CGTGTTCCAAGAAGGGTCTGCAGTTTAGAGCAAATGGCGACATTGGCTCGGCGGACGTGCAGATGAGCGC

GGACAACGAAAACTTTTCCGTTCTCAAAGCAAAGCAAACCGTAACGCACACTTTTGCGGGCGATTACTTG

TGTCATTT

>Ac-lef8 length=462 numreads=1

CGAGAGATACTTCTATATACTACAATTCCATTATTCAATGTACGATCGTAGAAAACGATGACGTCAAATC

GCTCATGTCCAAATTGGAGCAGTATTATTACTGTAATTTTATACATTTATTTCATACCACGCCCGTACCC

AAATTGATTGTGTCCCTAACCAATCTAAAGAATGCCATGCCCGTGTTTGAGTACAAAGAAAATAGTTGCG

TATCGGGTTTACCAAACGGCTACTCTGTGGCGGTGAACAAGTCCATTTTGCTCAACAATAAAATGTTTAA

ATTGTGGACGTTGGTGCGAGACAATAAACTCATGACCGCCGAAGATCCGTACATTCCGCACATTGCGCTG

CCCATTTGTTTATACAACAACAAAGTGAACAAGTTGAAGGGCAAGCTTGTGGTCGGCCCTAAACAGTCGT

GTTTAGTAAAATTTACGAATAGTAGCGACAAAAATTACGTCG

>Ac-lef10 length=258 numreads=2

TTCGCGACGGACGTCAACCTGATCAATTGTGTACTGAAAGATAATTTATTTTTGATAGATAATAATTACA

TTATTTTAAATGTGTTCGACCAAGAAACCGATCAAGTTAGACCTCTGTGCCTCGGTGAAATTAACGCCCT

TCAAACCGATGCGGCCGCCCAAGCCGATGCAATGCTGGATACATCCTCGACGAGCGAATTGCAAAGTAAC

GCGTCCACGTAACAATTATTCAGATCCCGATAACGAAAACGACATGTT

>AcOrf-63 length=191 numreads=2

TGATATTTGCGCTTTGCCCTCTGAGTTTCTGTTTCCAGAACATTTGCTCGACATGTTCATTGAATGATAA

TATAAATAGAGCGCATTTGATTGCATGCAATCAGTGTTTTATTAATTTTAGAGCAACATGTACGATAAAT

TTATGATCTATCTTCACTTGAATGGGCTGCACGGAGAAGCAAAATACTACA

>Ac-DNA-pol length=544 numreads=2

CCGATCGCTGCAATATTTAAAAAATGAAAATATATCCTTACAATGAACTCAAAACGCGCTTTGCAGAATA

TGCAAAACCAGGAGAATTCAATATTACTTCAGCCGATACGTTTCGTATCATTCGTTTGCATTACGATGAA

AACAAGGTTGCTTGTTTGCATTTTGTAATACAAATATCAAAGAACGAGTGCTGCAGTTTTACTTTAAAGT

AAACTAAATTTGTATTCTTACAAACAATGCTACGACAAGCACATATTTCCGTCTTGCCGCAACAAGTGCA

TCAGCTACACCACGTTTGTGGCGCCGGGCGTGGAAGGAAATTATTTTGAACAAGATAAAACGTGATTAAA

TACGAAAGAAACAAAGCAGCGCCATCGGACAACGCGGCGTGTTTGGACAAGTTTCTTCACAACGTTAATC

GCGTACACATGCAAACGCCGTTTGTGGAAGGCGCCTATATGCGATTCAAGAAAACACAACGCTGCCAAAA

TAACTATGTCGGTGGATCGACGACGCGCATGTTTAATTTGCAACATTTTAATGA

>Ac-lef3 length=1207 numreads=18

AGAGAAAATTACAAAAGGATTAGTGGTAAATTGATGAGCAAAATGACGTTGAGTATCGATAATGAATACC

ATTACACTTTTCGAATAATGTCCGACAACAAAATTCAAGAATACTACGGCGATTCGCAATCTTTCAAAGA

CATGGAAGAAGGCAAGTGCTATGACATTAGTTTAAATTATGTGAAACAAAGTTTAGTCAAATGATTCAGA

TCAACGAATACAAAGAATGTGAAATGGAAATTGAAAACTGCAACCCCCATGAGCGACTATTTGACTAACA

AACATTTTGAAAATGAAGATGGCGTCAATATTATTGTAAAGTACAAAATTATCTACAAGAAAATCAACAG

CGGTTTGTACAAAGTTGTTTTTGAGGTTGTGTACAAAAATCTCAACGACGATCCCGACGTGGTTCAAGTG

GAATGTTCTGTCAATGCGAAAACACTAATAAATCTATTCAAGAACAATATAAAGGGGTCTGACGACATCA

ATGAGGTGTTTAAATATTTAAAAGACAATGAAAACCAAATCTTTACCATTTACAGTATAAAGTGTCAACA

AATTTTTAATGGATCAAACGTGTACATGAATTGGAACGTGGTCAACTCTACGCGCATCGAATTGTGCGAG

GCTAAAGAAAGCGAAGCATATTCAAATTTGCAAAATTGCACAAACGCAAAGATCAACATTAGTCGATCGA

ACAAACACGTTGCTTCGTACAATGTAAATGTGTTAAAAAGCGAATTGGAAGAAAATGACATGGGCGATAA

CAAGTTTATTGTGCAGTTTAAAAGCGACGAGTTGAACATTGCCGATTCGGATGACTGTTCTACCTCTTCC

GACTTGGGCAAATGGAACAAATCTGTTTTCTACGTCAACACAAATAAAAAACCGAAGCGGATTCGCTACA

AAAGCTATGTGCGGATTTCAATCAAATCTCAATGCTGCTGGAAGATAATTTAATCAAAGTAACTATTTAC

GTAACTGTCGAGAACGGCGAGAATCACAACATGAACGTGTTGGGACTGTTGAAGTATGACGAAGATGAAA

ATGAATATAAATTTTTGTAATTCTATTCGACGTTTGGTTGAACGCTGGCCGCTGTTGTTCGCGCCAACGT

TTCGTACCTTTTAAGCATGCTAGTTTTCCACTCAAATATGTCTTGAGCTTTACGATTATATTTAACAAAT

AAAGAGTTTAATTCATC

>AcOrf-68 length=357 numreads=4

CTTCTTGGGGCTACTGGCCATAGCCATGCGTTTTATAAGCGGCTCACCGCTACTTTCTCCAGACAAAGAT

CTTTTGGTCGCCATATTGCTGTTGTCGATATGTGGGAATCTATCCGATGGCAAATACTGAATGGCGACGA

AATCGAAGTGTCGCCAGAGCACCGTTCGTTAGCGTGGAGGGAGTTGATTATAAACGTGGCCAGCAACACG

CCGCTCGACAACACGTTCAGAACAATGTTTCAAAAAGCCGATTTTGAAAATTTCGACTACAACACGCCGA

TTGTGTACAATTTAAAAACAAAAACTTTAACAATGTACAACGAGAGAATAAGAGCGGCTCTGAACAGACC

CGTCCGA

>AcOrf-69 length=387 numreads=4

AATGTTGTATTTGAGATTAGCATCAAGTGTGGCAACGCGTGCGATCTGGTGTTGGCAGATGGCTCGGTTG

ACGTTAATGGACGCGAAAACGAACAAGAACGTCTCAACTTTGATTTGATCATGTGCGAGACGCAGCTAAT

TTTAATTTGCCTGCGTCCCGGCGGCAATTGCGTTTTAAAAGTTTTCGACGCGTTTGAACACGAAACGATC

CAAATGCTAAACAAGTTTGTTAACCATTTCGAAAAATGGGTTTTATACAAACCGCCTTCTTCTCGGCCTG

CCAATTCCGAACGCTATTTAATTTGTTTCAATAAATTAGTTAGACCGTATTGTAACAATTATGTCAACGA

GTTGGAAAAACAGTTTGAAAAATATTATCGCATACAA

>AcOrf-70 length=238 numreads=1

GCCAATTTGTGCTTGAAAACCCTGCCTTACAAGTTTGAGCCGCCTAAGTTTTTACGAACAAAATATTGCG

ACGCATGTCGCTACAGATTTTTACCAAAATTTTCTGATGAAAAATTTTGTGGACAATGCATATGCAACAT

ATGCAACAATCCAAAAAATATAGATTGTCCATCATCATATATATCGAAAATTAAACCGAAGAAAGAAAAC

AAAGAAATATATATTACCAGCAACAAGT

>AcOrf-70 length=554 numreads=3

GTTACATGTGTTTGGAATATAAAAAGAATTTATACAATGTAAATTTGTATACGATTGATGGTCATTGTCC

TTCGTTTAAAGCCGTTGTTTTTCATGTATAAAAAGAATCAAAACGTGCCAAGTTTGCAATCAACCTTATT

GAAAATGTACAAAGAGAAGCAAGAAGAGCGTTTGAAGATGCAGTCGCTGTACGCAACGTTGGCCGATGTA

GATTTAAAAATATTAGACATTTACGATGTCGACAATTATTCTAGAAAAATGATATTGTGTGCTCAATGTC

ATATATTTTGCACGCTGTTTTTTGTACCAATACCATGCAATGTTTTTGTCCTCGACAGGGTTATAAGTGT

GAATGTATATGCCGACGATCTAAAATATTTTAAAAATAATGTATTGTGTGTTAAAAGTAAAGCGGCTTGT

TTTAATAAAATGAAAATAAAACGTGTTCCAAAATGGAAGCATAGTGTAGATTATACTTTCAAAAGTATAT

ACAAGTTAATAAATGTTTAATTTTTAAGGATATTGTTATGGAATAAACTATAAAATTAAAAAAA

>Ac-IAP2 length=554 numreads=3

CAAGATTAACGCCAAGCAAATTAAACGACACACGTATTCGAATTATTGCATATCGTCAACCAACGCGTTG

ATGTTCAATGAATCGATGAGAAAAATCATTTACGAGTTTTAAAAGCTCTCGGCGTCAGTTTGCATCACAA

TCCGTGGTCGTTGACATGTTGGCTCGTCGCGGCTTCTATTATTTTGGCAAAGCCGGCCATTTGCGTTGTT

CCGGATGCCATATAGTTTTTAAATATAAAAGCGTAGACGACGCCCAACGCCGGCACAAACAAATTGCAAG

TTTCTCAACGCAATAGAAGACTATTCCGTCAATGAACAATTTGGCAAACTCGATGTTGCGGAAAAGAATA

CTGGCTGCCGATTTGATTCCTCCGCGGCTAAGCGTTAAACCTTCGGCGCCGCCCGCCGAACCGCTAACTC

AACAGGTCTCCGAATGCAAAGTTTGTTTTGATAGAGAAAAATCGGTGTGTTTCATGCCGTGCCGTNACCT

GGCTGTGTGCACGGAATGTTCGCGTCGGTGCAAGCGTTGTTGTGTGTGCAACGCTAAATTATGC

>AcOrf-73 length=221 numreads=1

TGGACCAATATCTAAACAGCGACACGCCCGACTACACCATAGAATCCCCTAAACGCGCCGGGCAAACAGT

TTGATTTTTTAGATGAAATGCTAACGAAAAACTAATCGAAAGCAACGCCATAGTGTTTGACGAAAAAGTA

AAATTTAAAAATTATTCACAACAACATCAACATGTGTTAAATTGGTGTATTAATTAATTACAATAAAACA

TTATGTACAAT

>AcOrf-74 length=502 numreads=4

AACCGGATATAGTTAAAACCGAACAGGCCGACGCGCCGGCAACAAGCGGCGATAATAATCGAAAAGTGGT

CGATGCGAACGTGGACGAATACACGGTGGACGGTTTAAAACTTAAATCAAAATACGTCGCCTATTACAAA

TGTTTGAAAATACTTGTAGATTTTTTAGTAATGTACGTTAGCAAAGAGGTCAGCATGAAAGAATACGAGC

AAGTGTACACGTTAGGCAGACAGTTGTACGAGGTGTTGCGCAGCATTTTTGTCGACGAGCCGTTCAAATT

GTGGTTGGAACGCAACACGCACGAATTTGACAACAATAAAGCAAAATTTTAGAAACTTTACAGAGCGAAT

TGAAACTTGCGTTGGCCGATAAGGACAAATTGAAAACGTGCACGTTCAAAGATATCATCACCAATTTGTT

GAACACAAAATTGGATTGCAAATACGATTGCGCCGACGAGTATATTAAGCCGAATTGTATAGTGGACACG

TACAATTGTTGC

>AcOrf-75 length=449 numreads=4

GTTAAAATGTCCAATTTAATGAAAAACTTTTTCACCGAACTGGTCAAATCGACGACGTTCACCACCAAGG

TGTCCGTGGTCAAAACCACTCTTAGCAATTGGCTGTGCGAACAAGTCTATCCGGACAAGGATTTTTCGCT

TAAACTCAAACGAGTAGTTAACATGTTTTTAAACAATGAAATTGAAAATAACAAAATCTACAAACTAGTA

GAAACGGTCGACTCTTCTAATAAACTCAGTAGGCGACAGGTTGATTTTTTAATACATGCATTGTTAAACA

ATGTCAGTGTAACATTTACATTGCATAGATTCGTGGACGACAACGTTTTAACACAGGACGAGCTCAGTTT

TTTAGCAAATTTTCTAGTCACAAAAATGGATGAAGCATACCAACTGCCAGCGTATTAATAGTAAGATCAA

TAAAAATGAAAATAAATTTGTGCAATATA

>AcOrf-76 length=207 numreads=3

AAAAAGGAAAACAGCATTCTTTTTGTATCTGCTCATATTGTTTCTCGTGTTTATTATCGTCAGCCCGGCC

ATTATAAGTAAGAACACCGAGTCTACCGTAGAAGACATACCGAGTCATAAAGCTAAGAGCGTCCGAAAAA

AATTGGAGATCGAGCAAGCGCTTGATGCGATTCTAAACAAAAATACCAGCTCAATAGATTGATAAGT

>AcOrf-78 length=398 numreads=2

ATTTCAAATTGCGTCTCATCAAAGCCAAACCACTTTTGTTGCATAAATTCCTGTTAGCAAATAAGTTGTA

AACATGAATTTGGACGTGCCCTACTATCGGTTGGGCAACCACGAAAAGGTGGAATACATCCCGTTGAAAT

TGGCGCTCAACGACGATACTGCCAGCGAACCGCAACAATTTAGCGAGCCCGTTCACAAAATGCCAATAAA

CGACATGGTGGGCTATGACAACACGACGAGCAACGTGTCGGCGGGAATTATAATTTTAATTAGTGTAGTC

GCTTTTATAGCTTTATTCTTACTGCTGTATGTAATATATTATTTCGTAATATTAAGAGAACAACAACAAT

ATTCGGATAGTATTGACACCGATTCTCCTTTTGTTTTAATAAATTTGA

>AcOrf-79 length=192 numreads=1

TATCACAGCGCGAGCGCGTACGACTACAACACCGCCGCCCGCATGGAATACAATCTTAAGCGTAAATGCA

GCAAGTATTTCAAATTGCGTCTCATCAAAGCCAAACCACTTTTGTTGCATAAATTCCTGTTAGCAAATAA

GTTGTAAACATGAATTTGGACGTGCCCTACTATCGGTTGGGCAACCACGAAA

>Ac-gp41 length=225 numreads=1

GTGCCGCCGGCGACGGTGCCTACGACCGTCGCCACGCGCGACAACAGGATGGATTACACGAGCCGCAGCA

ACAGCACAAACTCGGTAGCGATTGCACCGTACAACAAGAGCAAAGAACCGACGCTCGACGCCGGTGAATC

TATTTGGACAACAAATGTGTGGATTTTGTCAAAAGATTATTAGATATTACAGGTGCAATGACATGTCAGA

ACTTAGTCCTCTCAT

>Ac-gp41 length=389 numreads=1

CAATTACATGGCCGAGCTGGCGACGCGAGCCGGCAAGCAGCCCAGCATGTTCCAGAACGCCACCTTCTTA

ACGTCGGCCGCCAACGCGGTCAATTCGCCGGCCGCTCATTTGACCAAAAGCGCTTGCCAGGAGAGCTTGA

CCGAATTGGCGTTCCAAAACGAAACTCTAAGACGTTTTATTTTCAACAAATAAATTACAACAAGGACGCC

AACGCGATTATTGCCGCGGCTGCGCCTAACGCCACTCGGCCGAACACGAAAGGGCGCACTGCATAATCAT

GGCGACGACTCTGTACACCAACAAGGTGTGGTGCGTGTACATTCTGCGGCAAGACAATGGAAAATTGTAC

ACGGGCATCACGAGCAATCTTAACAGACGCATAAAACAG

>AcOrf-81 length=566 numreads=30

CGCTAAACAAGATTAAATACGATAGTGAACTGTTGCTTCATTATTTGTACGATGATCACCACAATAAAAA

CTCTGATTACGCAAATAACAACATTAATGTTATTAAAATATCAAAAGTAAAGGTTAAAAAACGGGCGCCT

CGATATTAGCCCATTATTTTGCACAGATACACGTGTCCACTGGGTACTCGTTTGAATTTCATCCAGGCAG

TCAACCGCGGACATTTCAAACGATACACACCGACGGCCTCATTATAAAGGTGTTAATTTTGTGCGACGAA

TGTTGTAAAAAAGAACTGCGTGATTATATCAAAGGTGAAAATTCGTTTAATGTGGCTTTTAGAAATTGCG

AAAGCATTTTGTGTCGGCGCGTCAGTTTTCAAACGGTGCTGTTGACCTGCGCCATTTTGTTGTTGTTATT

CAACGTTGAAAAATTTTCCATGATTAACTTGTTGATAATTTTGTTAATTTTATTATCGCTTTTTTGTCAC

AACAACTATATTATAAGTAATCCGTATATTGAGTTTTGTAATCATAAGAGTACAAATAAAAAGTATGACA

GATAAG

>Ac-TLP length=282 numreads=30

AAAACGGGGCGTTGGCGCCCATATTATCAAGGTGGCTAGCTCGCCGCAACTCAGATTGTTGTACAAAAAC

GCCTATTCTACAGTGTCGTGCGGCAACTATGGTGTTTTGTGCAATTTGGTGCAAAACGGCGAATATGATC

TTAATGCAATAATGTTTAACTGCGCCGAAATAAAGTTGAACAAGGGCCAAATGTTATTTCAAACAAAAAT

TTGGCGGTCCGATAATAGTAAGACGGACGCTGCTGTACACACGTCGTCGCCCAAACGCACTGTAGAAACC

GA

>Ac-p95 length=180 numreads=2

AGTAATATTCGTCTTCAATTATAAAATCTAGTACGTTTTCATCTTCACTGTTGATTTGGGCGTTCACGAT

GATGTCTGGCGTAATGTTGCTCATGCTTGCCATTTTTCTTATAATAGCGTTTACTTTAATGTATTTGGCA

ATTTATTTTGAATTTGACGAAACGACTTTCACCAAGCGGC

>AcOrf-84 length=580 numreads=9

TGTAAATTGTTATTCTTTTATTTTAAAAACATTTAAACCACAAATCATACAAAAATAATCTATATACAAA

TTACTCTGCAATTGTTTCTCTTCAACGACATGACCCCGATCACAATTGGCAAACCTTTCCAATTTTGAAT

TCATGAAATATCCCACGCTGCGCTGCGGCACTTTTCTTTGTAGCAAAGCCGGATTAAAAATAATTGGTAA

ACCCAGCGAAAATATAATGTCTCGCATCAAACAAACAGCATTTAACGATTTTAACTGACGAAAAGGTTTC

TTGAGCGAAACGGCTATTTTGTCCAGACACAAATCTTGCAAGCTTTTCAGCGATTTGTTTTTGCGTTTTA

TTACTAACTGGTTGTTAGGTCCATATTCGTTTACAATCGCAACGTTAGGCATCAAAGATAAATAATCTGA

CAAAAAGCGAATATGTGATACATCTTTATAACGAACTTGAATAATGTCACCCACTAAACAACCGGGCACA

GTTGTGTTATTAGGGTATATTCGAAACATGGTGACAAGTAAATGAAAAAAATTTAACTTTGTGCGGTTAT

TTATACTTGCTATCATAAAC

>Ac-PNK/PNL length=505 numreads=6

ATTAAATCCAGAAACTATTAAAAAGCAATATAAAAAGTTTCTAAAGGTAAATAATTTCGAATATTACGTT

CCTGTCGGCGACAAATTTCCCCGCGCTGTATTATGTGATTTAGACGGTACAGTAGCTTTGCCCACAAATA

GATCGTTTTATGATTTTGATAATAGAGTTGCTCAAGATGAAGCCCGCTTAGACGTTATTACTTGCGTTAA

GTATTTGGCAAATTGCCACGACGCAATTATCGTGTTTATGAGCGGGAGAAGCGTAATTTGTGAACAGCCC

ACTCGAAACTGGATTGAAAAGTATTTTGATATTAAATCATATAAATTGTTTATGAGGCCTTCAGACGATA

CTTGTAAAGATTATTTATTAAAATTAAAATTGTTTAACAACTATATCAGAGGAAAATATAATGTAATAGC

GGTGTTCGACGACAGGCCGTGCGTTGTTCGCATGTGGCAAGATTTAAAATACCTACAGTGTTTAATGTTT

GCAGGGATTACTTGG

>Ac-p15 length=374 numreads=6

TGAATACAAGGGGTTGTGTTAATAATAATAAAATGATATTTATGAATGCTTTGGGCTTGCAACCTCAAAG

TAAATTGAAAATTATTGCACATAAAATACTAGAAAAATGTAAACGTGACGCGTACACGCGTTTCAAGGGC

GTAAAGGCGATCAAGAATGAACTAAAAACATACAATCTTACGTTGCAACAATACAACGAGGCGCTCAATC

AGTGCGCTTTAAACGATAGCCGATGGCGCGACACAAATAATTGGCATCACGATATTGAAGAAGGTGTGAA

AATAAACAAGAGACATATATATAGAGTTAATTTTAATTCTAAAACCCAAGAAATTGAAGAATATTATTAC

ATTAAAGTAGAATGTTATGTAAAC

>Ac-cg30 length=448 numreads=6

ACGACGCAGAAGACAACAACGCAAATGCCGACGACACTATGCTGTCTGAAGCACAAGCGATACTAAAAAA

ACTACAGGTAGACATTGCCGAACAAACGCAGCTCAACATTAAACAACAATTAGATTTAGACAAATTACAA

CAAACAAGCGTTTCTATGCAAGAAAAGTTAGACAAAATTAAAAGCGACTACAACAACATGCATAAATCTT

TTAAAGAATTGCAACTGAAACGAATTACAACTGAAAAGGCGCTAAAATCGCTCAATGACGATTACGCAAA

ACTTGCGTCTAAAAACGCCAAATTGAGTAGCGAAAATAAGGTTTTATCAAATAAAAATATTGAATTGATT

AAACACAAAAATTTATTACAAAACGAGTACACAACATTACAATCATATAAATGTATAACCAACGCCACTA

TTACCACAAATGTTACAATAAATGTAGA

>Ac-vp39 length=500 numreads=2

GCCGTTTTCGTCGTACAAATCGAAATATTGTTGTGCCAGCGAATAATTAGGAACAATATAAGAATTTAAA

ATTTTATACAACAAATCTTGGCTAAAATTTATTGAATAAGAGATTTCTTTCTCAATCACAAAATCGCCGT

AGTCCATATTTATAACGGCAACAATATGGCGCTAGTGCCCGTGGGTATGGCGCCGCGACAAATGAGAGTT

AATCGCTGCATTTTCGCGTCCATCGTGTCGTTCGACGCGTGCATAACATACAAATCGCCGTGTTCGCCCG

ACGCGTATCATGACGATGGATGGTTTATTTGCAACAACCACCTCATTAAAACGTTTAAAATGTCAAAAAT

GGTTTGCCCATTTTCGACGAAGACGACAATCAATTCAAATGACGATCGCTAGGCATTTAGTTAGGAATAA

GAAGAGGTATCAAGCGAATTTTAATCCAAGCGCAACCAATTACCAAGACGTGTTTAANCTAAACNGTATG

ATGCAAGCCG

>Ac-vp39 length=359 numreads=2

AATCTAAACAGTATGATGCAAGCCGAACAGCTAATCTTTCATTTGATATATAACAACGAAAACGCAGTTA

ACACTATATGCGACAATCTAAAATATACCGAAGGTTTCACAAGCAACACGCAACGCGTTATACACAGCGT

TTACGCAACTACAAAAAGCATTCTGGACACCACAAACCCGAACACGTTTTGTTCGCGGGTGTCGCGAGAC

GAATTGCGTTTCTTTGACGTGACCAACGCCCGAGCGCTTCGAGGCGGTGCTGGCGATCAATTATTTAACA

ATTACAGTGGATTTTTGCAAAATTTGATTCGACGCGCAGTAGCGCCCGAGTACTTGCAAATCGACACGGA

GGAATTGAG

>Ac-lef4 length=581 numreads=3

TCCACGATGGCGAACAAGATATCCAAACTGCTGACTTTGTTGGAAAATGGGGACGCTTCAGAGACGCTGC

AAAACTCGCAAGTGGGCAGCGATGAAATTTTGGCCCGCATACGTCTCGAATATGAATTTGACGACGACGC

GCCCCGACGACGCGCAGCTAAACGTGATGTGCAACATAATTGCGGACATGGAAGCGTTAACCGACGCGCA

AAACATATCACCGTTCGTGCCGTTGACCACGTTGATTGACAAGATGGCCCCTCGAAAATTTGAACGGGAA

CAAAAATAGTGTACGGCGACGACGCGTTCGACAACGCGTCCGTAAAAAATGGGCGCTCAAATTGGACGGT

ATGCGGGGCAGAGGTCTGTTTATGCGCAATTTTTGCATTATTCAAACCGACGATATGCAATTCTACAAAA

CCAAAATGGCCAATCTGTTTGCGCTAAACACATTGTGGCCTTTCAATGCGAGGTTATGGACAAACAAAGA

TTTACATTACAGATTTGCTGCAAGTGTTTAAATACAAATACAACAATCGAACACAGTACGAATGCGGCGT

GAACGCGTACATACGCTATAG

>Ac-lef4 length=173 numreads=1

AGTTGAGATACGTCAATATAAATGGATGCCAACAACCGAGTTAGAGTATGACGCCGTGAATAAGTCGTTT

AACACACTCAATGGGCCATTGAACGGTCTCATGATTTTAACCGACTTGCCGGGAGTTACTGCAACGAAAC

TATTTACGAATGTGTAATCACGGACACGACAAT

>AcOrf-91 length=129 numreads=1

TATTTAAGCAACATGTGGTACATTATTGTGATAATAATAATAATAATATTAAATTTATTTATAATAATTT

TATTAACTTTAAAAATATTAGACGACATTCAGGAATTGTATCCAAATCCACCAATTGTA

>AcOrf-91 length=320 numreads=3

GCCGCCTTCTCCTATTCCTCCTACACCTACACCTTCGCCGCCGCCTTCTCCCAGTCCTTTGGGCGAACCT

ATGTATTATCCTTCTAATATAGATACAAATGAAGCATTAATAAATTTTCTTAGGCCGTTGTGTGCAACTG

ATAGAACTCGAGCAACATACGCTGTGCCGTGGAATTGTAACGGGATATTTCATTGCAATTACTTCTCTAT

TCCTTTTTATATCCTCAGCTGTCACAACAATGCGTATTCGTTTGTTAACGATGGTTGTATTGACTTTAAC

AGCTCGGACTGCCCGTTGTATCCGCTTAACGTGCTTTAAT

>AcOrf-93 length=556 numreads=2

GTTTAGGTAGAAGGTGCGTCGATTTAAGAGGCTCGCAAAAAGGTCTATGAGACGAAACGAATACAACAGA

TAGCTGTCTTTGTAACGAGAAAAAAGCGGCGTCAGCGGTATCATGGCGACTAGCAAAACGATCGTGCTGT

ACTTGTGTCAGGCGCCGGCCACAGCGTCGTTGTACGTTAGCGCAGACACGGACGCCGACGAGCCTATTAT

TTATTTCGAAAATATTACAGAATGTCTTACGGACGACCAATGCGACAAGTTTACTTATTTTGCTGAACTC

AAACAGGAGCAAGCCTTATTTATGAAAAAAGTATACAAACACTTGGTGCTTAAAAACGAGGGTGCTTTTA

ACAAACACCACGTATTGTTCGATGCAATGATTATGTATAAGACATATGTGCATTTGGTCGACGAGTCTGC

GTTCGGAAGCAACGTTATCAACTATTGCGAACAGTTTATCACGGCCATTTTTGAAATTTTTACGCTCAGC

AGTAAAATCGTCGTGGCCGTGCCCGTCAATTGGGAAAACGATAATTTAAGTGTACTTTTGAAACTA

>Ac-odv-e25 length=520 numreads=2

ATTTGCACAACCTAAATCTCATTGGAATTGAAATTGTAAATTAAAACAAATCATGTGGGGAATCGTGTTA

CTTATCGTTTTGCTCATACTGTTTTATCTTTATTGGACGAATGCATTAAATTTCAATTCCTTAACCGAGT

CGTCGCCCAGTTTAGGGCAGAGCAGCGACTCGGTGGAATTAGACGAGAACAAACAATTAAACGTAAAGCT

GAATAACGGCCGGGTGGCCAACTTGCGCATCGCACACGGCGATAATAAATTGAGCCAAGTGTATATTGCC

GAAAAACCGCTATCTATAGACGACATAGTCAAAGAGGGCTCCAACAAGGTGGGCACTAACAGCGTTTTTC

TGGGCACCGTATACGACTATGGAATCAAATCACCAAACGCGGCCAGCACATCTAGTAATGTAACCATGAC

GCGCGGCGCCGCAAACTTTGATATCAAGGAATTCAAGTCCATGTTTATCGTATTCAAGGGTGTGACGCCC

ACTAAAACTGTAGAGGACAATGGCATGTTG

>Ac-helicase length=423 numreads=1

CGGTGCGGGCGGGCGTGCTTAAATCGTAAATCTGTACATACGCGCCAAACAACAAATGTGTTGTTGAAAT

CTTGTATTAGCGTTTCTATTCTATGTAAGAATGGATGGTGATTTTTGATCGAAATTATTAAATAAATTAA

CACGAAAAGTACAAACACGATAGCCAACATGATTGACAACATTTTACAATTTTTTTTGAAAAACGTGCCT

CAAGACAAAACGTACGAGATTAACAACTTGCAAGATGCAAATCATTTAATCATTAGAAACACGCGAACAG

GAACACGTAGATTGTTTGAGTACGTCAACAACTTTCAACAGTTCTTAAACACAATTAGAAACAACTTTAA

CGGTCCGTGCGCGAAACACGACATGGGCGCATCTTGCGAAGACACCGAAGAACCTGCAGAGAAACACGCA

GCT

>Ac-helicase length=337 numreads=1

TTACATCTCGGCACATTGGCGATGTGATAAGTTCTATAATGCTGAGCTCGTCGGCGCTAGTAGATAGCAT

GTAATTAACAAGAACAGGGTTTCCGACATGTTGCACAACCTAAATGCAAACCGTGTCGGTCAACGGTATC

GGGATCTCGACCAAAATGCAAATGCTACAAGAAATAAGATCAATAGAAAGCGCTAAAAGTCTGCTTGATG

GCGGACATGTTCGGTAACGATGCAGAATTGAGCGAATTGATTTGGATGCTAATTTTTACTAACAAAACGT

AACGTGTCGACCACGTTGATTCGCACGAACAGCGAATTTGTTAACCAACACGGAGAG

>Ac-helicase length=486 numreads=2

TTTTTACCAAAGAACACAACAAAATTATTCAGTACTTGTATCGAACAATTCACAAAATCGAATATGTCGA

CATGTTGATGGACAAGTTTAATGATAAACGTTTGTTTTTAACCGAATTGCGCGATGATGTTGCACGTGAA

CCCGATGTTCAGTTTGAAGAATCGGATAACATTTCCAAATTTTACACGCACCACGCCGACGCGTTAATGA

TCTTGAAGAAGTACAATGTGTGGTGGGACAAAATCATATTGGCGCGCAGCACGGACGATTTGCCCACTTG

GCTGACTCGATTCTACATGCGCATCATCATGTCCAAAGTGGACTTGAAGGAGTACTCGTACAATTATTTG

AAAAAGATTGTCGAAGGCTATTTGTACTTTAAACGCTTCACCAATTTTAATCACGCGAACGCGATTATGT

TGATGCATTTCGCCGCTAGTTTGGCCATTCCCGTAGATTACGGAAGAAGGCTATTTACATGCCCGG

>Ac-helicase length=878 numreads=5

CGCAGTTGTACACTATCAACGAGTTGAAACAATGCAGCGAGTCGACTTTAAGAAGCATGCCGATTCGAGC

AAGAGCGATTCCAAGAGCAGAAAATACCAAGGGCTGCTAAAGTATGAAGCCAACTACAAAATGTTAATTG

TTAACAACAAGCCGCTGTACGTGGACGATTATGACGACGGTGTACAAGACAGATTTTTGATTGTTTACAC

GAACCATAAGTTTGTAGACAGCGTCAAGTTTGCCGGCTCCGTGTACGAACACATCAAGTCGAAACAGTTT

CCAATAGAAAGCATGTATTACGAATCGCTTGTGACGCCAGTGCGTTTGTTTTTGTCGCACGTATTAATGT

ACAGACGCGATCCCAAAACGGGCTTTGTAGTATACAAAACGCTGCTGAGCAACGACCCTATGCATAAGCA

CAATCTAATGTGTTTAAGCACCAACAATAGTCCGCTGTATGCGCTCATTTACATTCTAAACATTAAAACG

GTGCGCAGTGCTACTATTACAATAGGAGAGGACAAAATGGAGGAAATGATAGGTATTGCCGTTCAGCATT

TAAAAAACTTTTGCACCCTTCGTTTGTTCAGTACAATTATAAAAAAATATAAACGCAAGTAGTTCAAAGT

CTTTTGTATTTAATGAACAAGTTTTATTACAGCAAATTAAAATAATTTAAAAATAATTACAATAAAACTA

CAAATGTGTTTTATAACATGACAATGGCGTTGAACAGAAACGATTTGAACACTAGTGTACCAAATTTTGT

ATGTTAATAAATAATATATTATATAAATAGACTTTTGTTTTATTAATTCATTTCTCTGTATGATTTATAG

TTTTGAGATAGGCGGTTTGTTCAGCATTAATGACGGTA

>Ac-p6.9 length=445 numreads=39

TAAATTACACAATTTAAACATGGTTTATCGTCGCCGTCGCCGTTCTTCAACCGGTACCACATATGGTTCG

ACACGCAGGCGCAGAAGCTCGGGTTACAGACGCCGCCCGGGTAGACCCCGCACATACAGAAGATCCAGGA

GCCGTTCATCGACAGGTCGCCGAAGTTACAGAACACGCTACTATTAACAACCAGACATTCCACACAGCCG

ACAGTAGCGAATGAACGAAGCGATTTCGTCGCCTGCCCTCGTTTGGCTTTCGACTGTTACAAAATCATGT

CTGCAAGATTTTAAACTAAGCCCGCTAAGCTCAAATAGTTTATTTTTATTACTGTTTTGTAAATAAATAA

CTTTATCATTCAATATTTGCCTGTGTTTGATTTTCTTTTTGCGTGTACACCCAGTGCTGGTATGTTTTCC

TTTTTTCAACAACAAACTTTTGTAA

>Ac-p40 length=445 numreads=5

CTCGAAACCACGTTTGACGACTACACGTGCAGACCGCAAATGACGCAGGTTCAAACGGACACGCTGTTGG

ACGCCGTGCGATCTCTGTTGGAGATGCCTTCGACCACAATCGACTTGACGACCGTCGATATTATGCGGTC

GTCGTTTGCACGCTGTTTCAACAGTCCGATTATGAGATATGCGAAAATAGTGCTGTTACAAAACGTGGCT

TTACAGCGCGACAAACGCACCACTCTTGAAGAATTGTTGATCGAGCGCGGCGAAAAGATACAAATGTTGC

AGCCGCAACAATACATCAACAGCGGTACCGAAATTCCGTTTTGCGACGATGCAGAGTTTTTGAACAGGCT

GCTCAAACACATAGATCCGTACCCGCTCAGTCGGATGTATTACAATGCAGCCAATACCATGTTTTACACG

ACTATGGAAAACTATGCCGTGTCCAATTGCAAGTTCAACATTGAGGATTACAATAACATATTTAAGGTGA

TGGAAAATATTAGGAAACACAGCAACAAAAATTCAAACGACCAAGACGAGTTAAACATATATTTGGGAGT

TCAGTCGTCGAATGCAAAGCGTAAAAAATATTAATAAGGTAAAAATTACAGC

>AcOrf-102 length=282 numreads=1

AGATATTTGTAACGTGCTAATGACAGAAATGATTGTGCCCGAAACATTTATAAGGCACATCATTACCAAG

TACCAATTGGACAACGAAATATCTCTGCTTATCGAACTCAACCATGATTGCTTCAATAAATGATACCGAT

ATGGACACTGACGACAACATGTCGCAAGCACGAAGAAACCGCCGCAACAGGCCGCCAGCAAGACCTTCGG

CGCAAACGCAAATGGCGGCCGTCGACATGTTGCAAACCATCAACACTGCGGCCTCTCAACGGCTGCGTCG

TT

>AcOrf-102 length=225 numreads=1

AGTTTAAAAATTTTGTCTACTCAATCTGTCGGAGCGCGCAGTCTGTTGGAACCGATGCAAGCCAACGCGT

CCACAATTAAGTTGAATAGAATACGAAACGGTCAACGTGTTAGTACTTTTGGGAAGCGTTTACGACAACA

CGATCCAAGTGATAGTTACAGAGTAAGTGTGCGTCGTCGGTCACGATGAGCGCTATCGCGTTGTATTGGA

ATAACAATTGCGGTT

>Ac-vp80 length=553 numreads=2

CTGTTGCAATATCAAAATTATGTAAACCAAGGCAATTATCAGAACATTGAGGGTTACGATAGTTTATTAA

ATAAGGCGGAAGAGTGTTATGTTAAAATTGATAGACTATTTAAAGAGAGCATTAAAAAAATCATGGACGA

CACGGAAGCGTTCGAAAGAGAACAGGAAGCGGAGAGATTGAGGGCCGAACAAACTGCCGCAAACGCTCTT

CTGGAGAGGCGAGCGCAGACGTCCGCAGACGATGTCGTTAATCGTGCCGACGCCAATATTCCCACGGCAT

TTAGCGATCCGCTTCCAGGCCCCAGCGCGCCGCGGTACATGTACGAAAGTTCAGAGTCGGACACGTACAT

GGAAACCGCCCGACGTACCGCCGAACATTACACCGATCAGGACAAAGACTACAACGCGGCGTACACTGCC

GACGAGTACAATTCCCTGGTCAAGACGGTTCTTTTGCGTTTAATCGAAAAGGCGCTGGCCACTCTAAAAA

ATCGGTTGCACATAACAACTATTGATCAATTGAAAAAAGTTTTAGAGATTATCTGAATAGCGA

>Ac-vp80 length=486 numreads=2

CTAAAAACCCTGTAACCAAAATCTCGAATCATATCGAAAATGAATGTAAATTTTTACGAGTTGCTGTTTC

CATTGACACTGTACAATGACAATGATAACAGTGACAAAACGCTTTCTCATCAATTGGTAAATTACATATT

TTGGCCAGTAACTATTTTCAAAACTGCGCTAAAAACTTCAACTATATGCGCGAAACTTTTAACGTGTTTG

GCCCGTTTAAACAAATCGACTTTATGGTCATGTTTGTTATAAAATTTAACTTTTATGCGACATGCGTAAT

TTTGCCAAATTAATCGACGAGCTGGTGCCCAACAAACAGCCCAACATGAGAATTCACAGCGTGTTGGTCA

TGCGGGATAAAAATTGTTAAACTAGCTTTTAGTAATTTACAATTTCAAACCTTTTCAAAGAAAGACAAGT

CGCGCAACACAAAACATTTGCAAAGACTAATAATGTTGATGAACGCAAACTACAATAGTTATATAA

>Ac-HE65 length=474 numreads=4

TTTGCATTTGATACGGGCAGCCATGCCAAGGGATACGCGGTCGAAAGCAGCGACACTGACTACCATATTT

ACACTAAATGTGATCGAGAGACCTTTGAAAAATTTATCGACAATAAAGAGCTTTTGAAAAATCGACATGC

AAAAGATGAATCGGGCAACGATGTCAAATACGTTGACTTGTACACGGGATTAATTGGCATTTTGACTGGA

AAATCTCCCGAATTAAGCATGTTCAGTAAACGTGAAGATTTTAAAGACAAATACGGTATTGAAAACTTGC

AATTGTACGAGTTTGTAACCAAGTTGATGACGGTGTCGATGGTGAAAATTATTTATACTTTGATGAGATA

CAAAATTTTGAACAATGCCAAAGGATTGTTGACAGCTCATGTTCAATTACGTGTACGTTGAATATTATTT

AGATTATAAACGTGCGCCGAAATCCACCAAAATTTTAAACATGTTGTTCAACGT

>Ac-HE65 length=407 numreads=2

ATGTATTGGATTTAAACAAAAATAATGGAGGATACAAAATCGACGAAACTCTAACGTTATTTGTAAAGAA

TGTCAAACTTTTAAAACTGTACGTAAAACTCATGCAACGCGGCGAGTATCAACAAGAATGGACCGAATAT

TTTCAACAATGGAAGCAACAATTGCAGGACAGACTGCACCACGTGCCAGAACCGCCGGAGCGCACCGACA

TTCGCCACAACATTGTCATGTACGCGTTAAATGAACGCGGACCCGTAATGCCCGAAGATGAGAATAAAAT

TGTGTATCAAATTTATCCATCTGTGTCGCATCTAGATCAAGGTAAAAAAGGTACGTTGGCCGACAAAGAA

ATTATTGTTCAAGAAAAGTTGGACGGTTGTAATTTCAGAATCATTTGCAACCAAAAT

>Ac-HE65 length=306 numreads=2

CGGTTCAATGACGGTTTTATTGTCTACGGCGAATTAATGGGTTGGAAAGACGACGCAAAAACGACACCCA

TTAACGTAATTAACTACGTGGACCAGAAAGAGAGTTTGAAATACTACGCTTACGAAATTCAATTATACGG

CGGGGAGTTTGTGCCCTTTGTGGAGGCGCAAGAGTTGTTGACTAACGTGGGGTTTAATACTATTCCGTGT

CACAAATATTTGTACAACGATTTGTAGAAAGGTTAATTCAAGTCTTAATGTTCTCCCCAAAGCCCGCTGG

AAGGATTTATTATTCGATGTGGAAAT

>Ac-HE65 length=309 numreads=2

TTTACAAACTGAAGTCTGATTATAAAGATTTAAACAAATTAAAAATAGAAAAGGGTCCTTTTGAGTGGTT

AACTTGCGATTACATTAAAAGCAACTGTGACGCCATAGACAAATCAGACATGATGAAAATTTTAATTTTT

GTTATAACATGTGCAAAGTAAAAAATTACAACGAAAAGTTATTGTTTAATAAAGTGTTTAATTTGTTTAG

ACAACAATTCAATTTAAATCATAATGATTATAAGAATTTGTATAAACAATATGTTAATATGTGTAAATGT

ACTGAATATAAATAAAAATTAAAAATTTT

>AcOrf-107 length=273 numreads=1

GGACAAAACAGATGAATTTGATTTTATTAAACCGGCATTGAAACCTTTGCCAGATGCAAGACCGCCATCG

CTTTTGGCCAACGTGATGAACGAACGTAAAAGAAAATTACAAAACACCAACTCAACGGCAAAATGTTTGC

TACCAGCACCACCGCCACAATTGCGTAAACTTGAAAAAAGAATCATTTATTGCCTTTGTTTTCTTTGTAA

TTATATTGTTGCATTTCTATTTCTAATATCATAGTTTTCTAATAAAGTAGTTTCATATTTTTG

>AcOrf-111 length=220 numreads=2

GTGACATTGCCTATGCTGCACTATGGATAATTATTCGGTGCAAATTTTTACAACAACGACCGCAACCTCT

CAAGCCGACAACGCTTCACGATGGTAACATCAAGAAATCGGTGTACGAAGATGTCACATATATTCGCAAG

CTGATGTGCAAAGAATTATGCCTGGCGAGCACGATCACAAGTTTTACAATTACGGTTATAACAAAGAAAC

AAATATAAAT

>AcOrf-112 length=395 numreads=2

CACGTTTATCGGTTAAGATAACTTTCAATATATAAAAGCGTTTGAATTGCGAGACCGTCAACATAACGTT

TATCAACGCGATGACTAAACGACAATTTGCTTTGCTGTTTGTGTGGCACCACGACAACCAATTTGTTTGC

AACACGGACGAATACCCGTTTTGGCACAACATTGAATACCATGCACGGCGCTATAAATGCATCGTTTTGT

ACTGTGTGGAAAACGACGGATCGCTACAACTGCCCGTTTGCAAAAAACATAAATCTCATAAATTATAAAA

AAAGCGTATCCTCATTATTATGGAAACTGTGTTGACAGTATAGTGAAACGTGCTGGCAAAAATTGATTAT

ATGAAAGTAACTGCAAATGTTAAAACCCCCACCTGTTGGACGTCG

>AcOrf-113 length=516 numreads=1

GTTGACAGTATAGTGAAACGTGCTGGCAAAATTGATTATATGAAAGTAACTGCAATGTTAAACCCCCACC

TGTTGGACGTCGCGTACAATTATTTGCTGTTGATGGACATGGATTGTGTGGTGCAAAGCGTGCAATGGAA

ACAATTGTCAACCGACACGTATTGTTTTGAGCCGTTTTACGACTCTCAAATTAAATGGTTGTACGCGCCC

AAAAGCGGGCAAAGTTTTGATAGTTATCTTGAAAACTATGCAACTCTAATTCGAGTCAAACAAGTGCAGC

AACATCGAAAAGAATTAATACTGCATTGTGTGGATTTTCTTACAATGAAAGCAAATGACAATTTTATGGT

GTTCAAAAATTATATTAACATGATTATAAAAGTGTATTTGCAATTTTACAATTACAGATTTCCTATCAAT

TTTGAGGACAACACGATGAAACCTTGTGTAAATTTAACTTTTAGACGTGGCGGCAGTTGGAAAACTCAAC

TGCAACCCGTATGCAATTATGTTTAC

>AcOrf-114 length=664 numreads=2

AATTTGTACAGACGCGTTTACAACTGAAATGTAAGAGATTCTGTCAACATGAGCAAGTACGCGCTTTTAC

AAAAAATGATCATCAACGAGTTGCTGTTTTTGAACGACAACGTGAATTACGCTACCAATAAACTGTTTAG

CAAAGATCAAGCTAACGGCGAGTTACACAAACTGTTGGCGATGTTGTTAAATTACAAAAGTCCAACGAAA

ACGCGCCAAATATTAAATTCGACCTAAAAAATTTGTCGTTTATGCTGGAGAACAAGGACAAGATCGATAT

AATTCAATTTGACGACATAAAAAACTATGTACAACCTGCGATAGTAAACTTGTTTGAATCGCACAATCGA

TCGTTGAATAACTACTCGACCGAACTGAGCACGCTACTGGAAGACGGCAATGAAAATCTGGTGCCAAACA

TCACCGACATTGATAACATTAAGTTGTCGCACATGCAACTGGCGCGCTTGTTATGTTACACGGCCGTCGT

TGAGTCACGCAACAGTAAACCCTGGAAAGCGATATTTAACAACGACACAAACGTTTTGACAGACTCTTTT

TCAACCATATCATGAATATTTTGAATATGATTAAAACCAATCAAGGATCGCTGGCTCACAACGTGTCGGT

GGTATACCATATTGAAATATCCAAATGAATTTAC

>AcOrf-116 length=229 numreads=5

AACACATCACACCTTCAATTACACTGTAATTTTTCAAATAGGTATTATAAATGTATTTCACGTCCCGCTT

TTTGAGCGCGTTAGGAACGAGCAATACATTAGCCGTCAGATGCATGACGGTAAAAATGAACGCGGTCGAC

GCCGAGTTATATAGACCCCGGTTTATTTTTGCGCAACCAGTCATTTCGTGCGTCACACCACGCTTTTCAC

ACTCAGTTAAGACGATCAT

>AcOrf-117 length=429 numreads=6

AAATGACTGGTTGCGCAAAAATAAACCGGGGTCTATATAACTCGGCGTCGACCGCGTTCATTTTTACCGT

CATGCATCTGACGGCTAATGTATTGCTCGTTCCTAACGCGCTCAAAAAGCGGGACGTGAAATACATTTAT

AATACCTATTTGAAAAATTACAGTGTAATTGAAGGTGTGATGTGTTGCAATGGCGATTGTTTGGCCGTGG

TGGTGTTGGACCGAAATCAGCTGCAAAACACGGACATGGAAGTGTTGGAGAGTTTAGAATACACTAGTGA

CAACATTGAACTGTTATGCGAAAAAATATGTGTGATAGTTGATAATTACGACAAGTATTACCAAAAAAAT

TGTGTATAAATAAAATACCAAAATTTATTATATCATTTTGTTTTATTTAATAATTAAAGAATACAACGCC

ACATCTATT

>AcOrf-118 length=634 numreads=7

TCTTCATGGTGCACATCTATCAAGTCTACATACGTGTACACTATTGCAATAATCACTAGAAGAAACAACA

GTATAATAGCAAAATGCATTTTACTTATTGGAGAATGTCCGAGTATTTTGTACGTACGAGATATTTGCGG

CCAACGCGATTATGTGCGCGCTCATGCCGTGTTTAGCAATTTTCTTCATTATAGAATTGTTATGTAAAAA

CTTTAACACTATGTATAACGTCATGTACGTGGTTTTGTTTGCAATTTTTCAATTGTTTGAAAAAGGCTTT

GATATCGCAAAAACAATTGCGGACAAATGGCATATTAGAAAAATTTTGTTATTTGTGAATAATGCGTATC

AACATTTAACGTTGTACGTTGAAAAATATCAAAGTATAAATTACGCTATCAAAATTGCAGCTATTTACTA

TAATATTTATGTATTGTATTATTCTGTAGTATTTTTACACTGTTTGTTATTTTTTAATGTGCACTCAAAA

ATAATAATCAAATTATTTGTTGTACTAGGAATAGATGTGGCGTTGTATTCTTTAATTATTAAATAAAACA

AAATGATATAATAAATTTTGGTATTTTATTTATACACAATTTTTTTGGTAATACTTGTCGTAATTATCAA

CTAT

>AcOrf-119 length=501 numreads=4

TCGCGTTGGCCGCAAATATCTCGTACGTACAAAATACTCGGACATTCTCCAATAAGTAAAATGCATTTTG

CTATTATACTGTTGTTTCTTCTAGTGATTATTGCAATAGTGTACACGTATGTAGACTTGATAGATGTGCA

CCATGAAGAGGTGCGTTATCCTATTACGGTTTTTGACAACACACGCGCGCCGCTCATTGAACCGCCGTCC

GAAATAGTAATCGAAGGCAATGCACACGAATGTCACAAAACTTTGACGCCGTGCTTCACACACGGCGATT

GCGATCTGTGCCGCGAAGGATTAGCCAACTGCCAGTTGTTTGACGAAGATACAATAGTCAAGATGCGTGG

AGATGACGGCCAAGAACACGAGACGCTTATTCGAGCGGGAGAAGCGTACTGCTTGGCTTTGGATCGAGAA

CGCGCCCGATCGTGTAACCCCAACACGGGTGTGTGGTTGTTGGCCGAAACTGAAACTGGTTTCGCTCTTT

TGTGCAACTGC

>AcOrf-122 length=183 numreads=4

TGGCCGCGTTTGTCACACTCGCCATGGGCAAAATCGTTGAAAGATCCATTAACAATTACTACGGCGCATT

TTGACCATCCCGAGGGGCTGAAACACAAGATTCGAAGACCTTACAACAAACACGAAGACAGATTGATTCA

ATGTAAAATTTATTTGTAATAAATTATATTTACACACAAAAAA

>Ac-pk-2 length=453 numreads=9

TATAGGGCGTGCGCTCGTGTCTGAATTTAGTGACGCGTTAACACTTAACTGTATACCATGAAACCCGAAC

AATTGGTTTATTTGAATCCGCGGCAGCACCGCATATATTTTGCGTCGCCTCTAAACGAGTACATGTTGAG

CGACTATTTGAAACAACGCAATTTGCAAACTTTTGCAAAGACCAACATTAAAGTTCCGGCGGATTTTGGC

TTTTATATTAGCAAGTTTGTTGATTTGGTGAGCGCCGTGAAAGCGATTCATTCCGTAAATATCGTGCACC

ACAATATTAATCCCGAAGATATTTTCATGACTGGGCCCGACTTTGATTTGTATGTGGGCGGCATGTTTGG

CAGTCTATACAAAACGTTTATCAAAAACAACCCTCAAAATATAACTTTGTACGCTGCACCAGAACAAATC

AAAAAAGTGTACACCCCCAAAAATGACATGTAT

>AcOrf-124 length=765 numreads=6

ATTTAATCAATCGAACCGTGCACTGATATAAGAATTAAAAATGGGTTTGTTTGCGTGTTGCACAAAATAC

ACAAGGCTGTCGACCGACACAAAAATGAAGTTTCCCTATGTTGCGTTGTCGTACATCAACGTGACGCTGT

GCACCTACACCGCCATGTTGGTGGGATACATGGTAACATTCAATGACTCCAGCGAATTGAAATATTTACA

ATACTGGTTGCTGTTGTCGTTTTTGATGTCCGTGGTGCTAAACGCTCCGACTCTGTGGACGATGCTCAAA

ACCACAGAAGCCCATGAAGTAATTTACGAAATGAAGCTGTTCCACGCCATGTACTTTAGTAACGTGCTGT

TGAATTATGTGGTGTTTTTGGACAATCAAATGGGTACAAATTTTGTTTTTGTTAACAATTTAATTCACTG

TTGTGTACTTTTTATGATATTTGTTGAATTGCTTATCCTGTTGGGCCACACAATGGGCACGTACACGGAT

TATCAATATGTCAAATCGTGTTATATGGTTATATTGTTTGTTTCAGTTATGAGTGTTACTATTGTTATGG

GTTTAGAGTGTTTGAAAACGAAACTAATTGATAACAGTTTGATGTTTAACGCGTTTGTGTGCGCTTTGTA

CATTGTGATTGCAATAATGTGGTCTTTAAAAATAATTTGACTAGTTATTACGTTTCAAATTTACAAAGTA

TTCAAGTTGTTCCGTTTTCATACAACGATCCGCCGCCACCGTTCTCTAACATTGTAATGGATGAC

>Ac-lef7 length=654 numreads=6

GCGCCCACGAGCAAAACGCATACGTTTACCATTAGAAATTATAGACACGATTCTGCAGTATTTGGATCCG

ATTTTACATGCAAAAGTCGTGGGATTAACAACGCGTGTAAAATGCAGACTTTTGCGCGACAACAACGTTG

AAGATTATTTAAAGCTGACACCTGCCAGTTATCACCCCACGACGGATCAGTTTATTTGTAATTATCTTGG

AATAACAAATCAGCCAATGGCGCCATATTTAGTACCTTTGCTTTCGTTTGGTAAAGCGAGCTGTGTGTTT

TTTAACAAATGCATACCCGAGGACGTGCGCATCGTCACGTTGAATTGGCCGTTACCATTGTTGGAAAACT

TTTTGTCCAAACAATTTTTATGGTACAAGTTGGCGAGAAAGTTGATCGAACACGAACGTCGAATGGATCG

CTGTGTAACACCGTCTACAGTGCAAATAAACTTATACGACGACAATGAAGATTATTTAAATTGTTTTAAT

TGCTTTAATTGTTGTAAAGATAATTTGGTTAGTTTTAATTGTTGTATAGTAGACTGTAATATCAATGATA

TGAATAGGTGTCCAGATTTACAAATTGATGTTTATTTAGACGACAACATAATAAGTTTGTATTTATATTT

TTTGTTTAGAATTTACAGAATTGT

>Ac-gp64 length=837 numreads=9

CGCAAATGAAGACGGGTCCGTACAAGATTAAAAACTTGGACATTACCCCGCCCAAGGAAACGCTGCAAAA

GGACGTGGAAATCACCATCGTGGAGACGGACTACAACGAAAACGTGATTATCGGCTACAAGGGGTACTAC

CAGGCGTATGCGTACAACGGCGGCTCGCTGGATCCCAACACACGCGTCGAAGAAACCATGAAAACGCTGA

ATGTGGGCAAAGAGGATTTGCTCATGTGGAGCATCAGGCAGCAGTGCGAGGTGGGCGAAGAGCTGATCGA

CCGTTGGGGCAGTGACAGCGACGACTGTTTTCGCGACAACGAGGGCCGCGGCCAGTGGGTCAAAGGCAAA

GAGTTGGTGAAGCGGCAGAATAACAATCACTTTGCGCACCACACGTGCAACAAATCGTGGCGATGCGGCA

TTTCCACTTCGAAAATGTACAGCAGGCTCGAGTGCCAGGACGACACGGACGAGTGCCAGGTATACATTTT

GGACGCTGAGGGCAACCCCATCAACGTGACCGTGGACACTGTGCTTCATCGAGACGGCGTGAGTATGATT

CTCAAACAAAAGTCTACGTTCACCACGCGCCAAATAAAAGCTGCGTGTCTGCTCATTAAAGATGACAAAA

ATAACCCCGAGTCGGTGACACGCGAACACTGTTTGATTGACAATGATATATATGATCTTTCTAAAAACAC

GTGGAACTGCAAGTTTAACAGATGCATTAAACGCAAAGTCGAGCACCGAGTCAAGAAGCGGCCGCCCACT

TGGCGCCACAACGTTAGAGCCAAGTACACAGAGGGAGACACTGCCACCAAAGGCGACCTGATGCATA

>Ac-gp64 length=510 numreads=8

TTTTGCTGATGCCGTGCACCAATCCGCCGGCACACACCAGTAATTGCTACAACAACAGCATCTACAAAGA

AGGGCGTTGGGTGGCCAACACGGACTCGTCGCAATGCATAGATTTTAGCAACTACAAGGAACTAGCAATT

GACGACGACGTCGAGTTTTGGATCCCGACCATCGGCAACACGACCTATCACGACAGTTGGAAAGATGCCA

GCGGCTGGTCGTTTATTGCCCAACAAAAAGCAACCTCATAACCACCATGGAGAACACCAAGTTTGGCGGC

GTCGGCACCAGTCTGAGCGACATCACTTCCATGGCTGAAGGCGAATTGGCCGCTAAATTGACTTCGTTCA

TGTTTGGTCATGTAGTTAACTTTGTAATTATATTAATTGTGATTTTATTTTTGTACTGTATGATTAGAAA

CCGTAATAGACAATATTAAATGTAATAATAAAAATTGTATCATTATTAATGTAATAATAAAAAATTGTAT

AGTTTTTAATTGTATATTAT

>Ac-PE/pp34 length=339 numreads=1

CGGCGTTGGAACCGCTAAAGATGTTATTTTGACCAGATTAAACACATTGCTTGCCGAGATTACAGACTCG

TTACCCGACTTGACGTCCATGTTAGATAAATTAGCTGAACAATTGTTGGACGCCATCAACACGGTGCAGC

AAACGCTGCGCAACGAGTTGAACAACACCAACTCTATTTTGACCAATTTAGCGTCAAGCGTCACAAACAT

CAACGGTACGCTCAACAATTTGCTAGCCGCTATCGAAAACTTAGTAGGCGGCGGCGGCGTGGCAATTTTA

ACGAAGCCGACAGACAAAAACTGGACCTCGTGTACACTTTGGTTAACGAAATCAAAAAT

>AcOrf-132 length=123 numreads=1

AGAGAATGCAGTTGTAGTATTAGAAAATGAACAAAAACGNTTGTATCCCATATTAGATACGCCTCTTGAT

AATTTTATTGTCGCATTCGTGAATCCGACGTATCCCATGGCCTATTTTGTCAA

>Ac-alk-exo length=565 numreads=7

AGCACGTCGACATCATGTTTGCGTCGTTGACCTCAGAGCAAAAGCTGTTATTAAAAAATATAAATTTAAC

AATTATGTGAAAACGATCGAGTTGAGTCAAGCGCAGTTGGCTCATTGGCGTTCAAACAAAGATATTCAGC

CAAAACCTTTGGATCGTGCAGAAATTTTACGTGTCGAAAAGGCCACCAGGGGACAAAGCAAAAATGAGCT

GTGGACGCTATTGCGTTTGGATCGCAACACAGCGTCTGCATCGTCCAACTCGTCCGGCAACATGTTACAA

CGACCAGCGCTTTTGTTTGGAAACGCGCAAGAAAGTCACGTCAAAGAAACCAACGGCATCATGTTAGACC

ACATGCGCGAAATCATAGAAAGTAAAATTATGAGCGCGGTCGTTGAAACGGTTTTGGATTGCGGCATGTT

CTTTAGCCCCTTGGGTTTGCACGCCGCTTCGCCCGATGCGTATTTTTCTCTCGCCGACGGAACGTGGATC

CCAGTGGAAATAAAATGTCCGTACAATTACCGAGACACGACCGTGGAGCAGATGCGTGTCGAGTTGGGGA

ACGGC

>Ac-alk-exo length=507 numreads=7

GGTTAACAAGAAAGGCACGCCCCAGTTCGAAATGGTCAAAACGGATGCGCATTACAAGCAAATGCAACGG

CAGATGTATGTGATGAACGCGCCTATGGGCTTTTACGTGGTCAAATTCAAACAAAATTTGGTGGTGGTTT

CTGTGCCGCGCGACGAAACGTTCTGCAACAAAGAACTGTCTACGGAAAACAACGCGTACGTGGCGTTTGC

CGTGGAAAACTCCAACTGCGCGCGCTACCAATGCGCCGACAAGCGACGGCTTTCATTCAAAACGCACAGC

TGCAATCACAACTATAGTGGTCAAGAAATCGATGCTATGGTCGATCGCGGAATATATTTAGATTATGGAC

ATTTAAAATGTGCGTACTGTGATTTTAGCTCAGACAGTCGGGAAACGTGCGATTCTGTTTTAAAACGCGA

GCACACCAACTGCAAAAGTTTTAACTTGAAACATAAAAACTTTGACAATCCTACATACTTTGATTATGTT

AAAAGATTGCAAAGTTT

>Ac-94K length=1505 numreads=28

CGACGAAGCGTACGATTACAGCAACGTAGAAAATATTCGATTCCCCGATTGTATTTTAGCCAACGATAGC

GGTGTGCCGGCCATATTGTTAACACATTACAACTTGTTTGAAACAATTCAGGGCAGTTTGACCAAGTTTA

AAAGCCGACTCGAGTTTCCTCTTTTATGGAGCCAAAATAAAGAGATTAAAATTCAATTGAATATTGTTAT

AATTTGGAAAGTTTGAAGCAGTTGATTCAGCATGGAACGCGTTTGTCACCGCGCAGCAGACGCCCTTTTA

CGGGCGCCATAGTGCCCAACGAGCAATTCGACGAATACAACGATTACGTGTTGGCGTGCACGTATTTTGA

CGCTAAAAAGGTCGCGTTTAACGCGGGCCTTATGTACTATTTGCTGTACAAGCACATAAACGACGCAGAA

TACATTGACGATAATGTAAAGGACTACTTTAAACGCTATGTCATTTACCGCATTAACAACACGGAATGCA

TGATCGGGTTCAGCAACCTGGCTATGGAGCCGCTGATCAAAGTAAAATTGCCCACGGCTTTGTGGTATGT

CTCGGAAATTTCAACGTTGCTTTTTAAACACGACAATCAACATTTTGGAAAGGAAAAGCTGCGGCAGTTC

GCGCATTTTGCCGAGGACATGTTGCAAATCCTGCAATGGTGCGACTACACGGACGTGAACGTTGAGGCGG

TAAAAAAACGAGCGTATTGTCTAAAACGTATCAACATGTTTAAACGCATGTCCGTTTTGGACGCTGTGGA

GTGGATTGCAAACAGAGCGTTTGAATGCAAAGACAAATTTATTATCAACAAGTTGACCAACGCGGACGCT

TTGCAAGATTTGAAATTTTTGAAAGTAAATCATAACGGAGTTGTTGACGAACATGTTTTAAACGACACGT

CCATAAACGCGGAGAGATATTTGTATTTCTATCATATAATTGAAGATTTTGACAAGTACATAAGCGTTGT

AGATAACACGATGCGCCCCGCGTTTGTGCTTGAAGAAGGAAAAACTTTTTACGACAGTTTATTGAAACAA

TTGCAAAGTGTACATTTCAACGGTCAAGAAATAACTTTTGAGAAATGTTCTCGTTTGGATTTTAACAGAA

TTTTGTCACTGCACAAATTGTACATTGAATGCGTCAAGAGTTTAAACAAATATCCTACGCTGGAAGAATA

TCAAAACTATGTATACAATCAAAAACATGTCAAATTTAATAGAATTGCCATTTTTCCCGAAAACATTCTA

CAAAACCTGGCCGCAGTGCACAATGAGTACGCAAACAAAATTGTTAATTTGCCAGTTGAGGAGTTTATTG

TTCGCGCCAACAATACCGTAAACCGGATTACGCGCATTCAAAACGAACGCGTTGGCAGCCCTTTGCAAGC

CGAAGAAATTGACAAGCTTATTAAACTTTCTGAACAACGAGTCAATATTTGTCGTAAATAAAATTGTATA

AACAAATGTATAAATAAAAGAATTATATATAATAA

>Ac-35K/p35 length=929 numreads=27

TTACCATTGCAAAATGTGTGTAATTTTTCCGGTAGAAATCGACGTGTCCCAGACGATTATTCGAGATTGT

CAGGTGGACAAACAAACCAGAGAGTTGGTGTACATTAACAAGATTATGAACACGCAATTGACAAAACCCG

TTCTCATGATGTTTAACATTTCGGGTCCTATACGAAGCGTTACGCGCAAGAACAACAATTTGCGCGACAG

AATAAAATCAAAAGTCGATGAACAATTTGATCAACTAGAACGCGATTACAGCGATCAAATGGATGGATTC

CACGATAGCATCAAGTATTTTAAAGATGAACACTATTCGGTAAGTTGCCAAAATGGCAGCGTGTTGAAAA

GCAAGTTTGCTAAAATTTTAAAGAGTCATGATTATACCGATAAAAAGTCTATTGAAGCTTACGAGAAATA

CTGTTTGCCCAAATTGGTCGACGAACGCAACGACTACTACGTGGCGGTATGCGTGTTGAAGCCGGGATTT

GAGAACGGCAGCAACCAAGTGCTATCTTTCGAGTACAACCCGATTGGTAACAAAGTTATTGTGCCGTTTG

CTCACGAAATTAACGACACGGGACTTTACGAGTACGACGTCGTAGCTTACGTGGACAGTGTGCAGTTTGA

TGGCGAACAATTTGAAGAGTTTGTGCAGAGTTTAATATTGCCGTCGTCGTTCAAAAATTCGGAAAAGGTT

TTATATTACAACGAAGCGTCGAAAAACAAAAGCATGATCTACAAGGCTTTAGAGTTTACTACAGAATCGA

GCTGGGGCAAATCCGAAAAGTATAATTGGAAAATTTTTTGTAACGGTTTTATTTATGATAAAAAATCAAA

AGTGTTGTATGTTAAATTGCACAATGTAACTAGTGCACTCAACAAAAATGTAATATTAAACACAATTAAA

TAAATGTTAAAATTTATTG

>Ac-p26 length=763 numreads=9

AAATCAAACGGACGTTATGGAATTGTATAATATTAAATATGCAATTGATCCAACAAATAAAATTGTAATA

GAGCAAGTCGACAATGTGGACGCGTTTGTGCATATTTTAGAACCGGGTCAAGAAGTGTTCGACGAAACGC

TAAGCCAGTACCACCAATTTCCTGGCGTCGTTAGTTCGATTATTTTCCCGCAACTCGTGTTAAACACAAT

AATTAGCGTTTTGAGCGAAGACGGCAGTTTGCTCACGTTGAAACTCGAAAACACTTGTTTTAATTTTCAC

GTGTGCAATAAACGCTTTGTGTTTGGCAATTTGCCAGCGGCGGTCGTGAATAATGAAACGAAGCAAAAAC

TGCGCATTGGAGCTCCAATTTTTGCCGGCAAAAAGCTGGTTTCGGTCGTGACGGCGTTTCATCGTGTTGG

CGAAAACGAATGGCTGTTACCGGTGACGGGAATTCGAGAGGCGTCCCAGCTGTCGGGACATATGAAGGTG

CTGAACGGCGTCCGTGTTGAAAAATGGCGACCCAACATGTCCGTCTACGGGACTGTGCAATTGCCGTACG

ATAAAATTAAACAGCATGCGCTCGAGCAAGAAAATAAAACGCCAAACGCGTTGGAGTCTTGTGTGCTATT

TTACAAAGATTCAGAAATACGCATCACTTACAACAAGGGGGACTATGAAATTATGCATTTGAGGATGCCG

GGACCTTTAATTCAACCCAACACAATATATTATAGTTAAATAAGAATTATTATCAAATCATTT

>Ac-p10 length=501 numreads=5

TATACTGTAAATTACATTTTATTTACAATCATGTCAAAGCCTAACGTTTTGACGCAAATTTTAGACGCCG

TTACGGAAACTAACACAAAGGTTGACAGTGTTCAAACTCAGTTAAACGGGCTGGAAGAATCATTCCAGCT

TTTGGACGGTTTGCCCGCTCAATTGACCGATCTTAACACTAAGATCTCAGAAATTCAATCCATATTGACC

GGCGACATTGTTCCGGATCTTCCAGACTCACTAAAGCCTAAGCTGAAAAGCCAAGCTTTTGAACTCGATT

CAGACGCTCGTCGTGGTAAACGCAGTTCCAAGTAAATGAATCGTTTTTAAAATAACAAATCAATTGTTTT

ATAATATTCGTACGATTCTTTGATTATGTAATAAAATGTGATCATTAGGAAGATTACGAAAAATATAAAA

AATATGAGTTCTGTGTGTATAACAAATGCTGTAAACGCCACAATTGTGTTTGTTGCAAATAAACCCATGA

TTATTTGAATT

>Ac-ME53 length=899 numreads=10

GTTTTTGTATGTTGTGGTTCACATCGAAAAGCCGTTGGACGATCCTGACCGCATAGACATCTGCTGTCAA

AAATGCTATCTATATCACAACGTTCCAAAAACCTCGTACGAAATATATCCTTCTATCAATTTGGTCGACC

TCAGCTATTTAGCCAGAGAGAGGTTTTTTTACCAATACATTTTCCCTGTAAGTTTAGAGCACACGACGGA

AGTTAAAGAATTGAGAATTGACGATCACAACTGCAAAGTGTTTGAAATAATTCGGCGCATAATTCGAAAT

CACAAAGAGCCCAACGAGCGTATTCAAACAATCGATCTTAGCACCACCGGCGGTCTCGTTCTCAGGGAAA

CATACACCAATATAGTGTTGCAGCGCTACCGAAGCATGTGCACCAGACCCGACGTGGTGGATGACGTCAA

CTGTTTTATACTTCAAGAGCCAAGCGAAATGATGGCCGCTCTGCAAGACAACCGATTTTCGGGTATCAAA

GGAACCGTGTTTGCCACTGTTAAAGTCAAAAAGTTTACCCAGGTATTGGACGGCGCAATCACGTTTCCTT

TGAAACCTACAACTAACAACTATTGCAAATTGTGTAAAAAACTAAACTGTATTATAAAAATCCCGTGTTA

TATTGTACCAAATGCGGGTTTACCAACGTTTATCATTTTCCTGAATACTCTAAATTCATGTATTACTTTG

AAGCTATCAAAAGCTTTGAAATGCACAACGAAATGATTATATATTATGATTTAAAAATGTATAAAAAGTT

AATTAATATTGTAAATAACAATGTCTAAATAAAAATGTATCGTTATAATATAATCAGATTAAATAAAAAG

TATTGTTATAATAAATGTAAATGTATTGCTATAATATAATCTAATAAATATTTCATTAA

>Ac-49K length=496 numreads=2

GCTCATGAACTATTTCAATTACTTGCAAAGCATGCAATTGAAACATTTGGTGGGCAGCACGTCGACAAAC

ATTTTCAAGTTTGTAAAGCCACAATTTAGATTTGTGTGCGATCGCACAACTGTGGACATTTTAGAATTTG

ACACGCGCATGTACATAAAACCCGGCACGCCCGTGTACGCCACGAACCTGTTCACGTCCAATCCCCGCAA

GATGATGGCTTTCCTGTACGCTGAATTTGGCAAGGTGTTTAAAAATAAAATATTCGTAAACATCAACAAC

TACGGCTGCGTGTTGGCGGGCAGTGCCGGTTTCTTGTTCGACGATGCGTACGTGGATTGGAATGGTGTGC

GAATGTGTGCGGCGCCGCGATTAGATAACAACATGCATCCGTTCCGACTGTATCTACTGGGCGAGGACAT

GGCTAAGCACTTTGTCGATAATAATATACTACCGCCGCACCCTTCTAACGCAAAGACTCGCAAATCAACA

ATTCAA

>Ac-odv-e18 length=222 numreads=2

AACAGGCTAACTCCAAACATGTTCTTGACCATCTTGGCTGTAGTAGTAATTATTGCTTTAATAATTATAT

TTGTTCAATCTAGCAGTAATGGAAACAGCTCGGGGGGTAATGTACCTCCAAACGCCCTGGGGGGTTTTGT

AAATCCTTTAAACGCTACCATGCGAGCTAATCCCTTTATGAACACGCCTCAAAGGCAAATGTTGTAGATA

AGTGTATAAAAA

>Ac-odv-ec27 length=1262 numreads=16

CGAAAGTTCGTCTGAGTTGAAATCCCTTCGCGATTTGAATCCGTGGGTTCAGAACACGCTTCTCAAATTA

TTAATCCCCGACTCGGTACAATAATATGATTTACACTGATCCCACTACTGGCGCTACGACTAGCACAGAC

GCGCCGTCCACAAACTATTTAAACAGGCTAACTCCAAACATGTTCTTGACCATCTTGGCTGTAGTAGTAA

TTATTGCTTTAATAATTATATTTGTTCAATCTAGCAGTAATGGAAACAGCTCGGGGGGTAATGTACCTCC

AAACGCCCTGGGGGGTTTTGTAAATCCTTTAAACGCTACCATGCGAGCTAATCCCTTTATGAACACGCCT

CAAAGGCAAATGTTGTAGATAAGTGTATAAAAAATGAAACGTATCAAATGCAACAAAGTTCGAACGGTCA

CCGAGATTGTAAACAGCGATGAAAAAATCCAAAAGACCTACGAATTGGCTGAATTTGATTTAAAAAATCT

AAGCAGTTTAGAAAGCTATGAAACTCTAAAAATTAAATTGGCGCTCAGCAAATACATGGCTATGCTCAGC

ACCCTGGAAATGACTCAACCGCTGTTGGAAATATTTAGAAACAAAGCAGACACTCGGCAGATTGCCGCCG

TGGTGTTTAGCACATTAGCTTTTATACACAATAGATTCCATCCCCTTGTTACTAATTTTACTAACAAAAT

GGAGTTTGTGGTCACTGAAACCAACGACACAAGCATTCCCGGAGAACCCATTTTGTTTACGGAAAACGAA

GGTGTGCTGCTGTGTTCCGTGGACAGACCGTCTATCGTTAAAATGCTAAGCCGCGAGTTTGACACCGAGG

CTTTAGTAAACTTTGAAAACGACAACTGCAACGTGCGGATAGCCAAGACGTTTGGCGCCTCTAAGCGCAA

AAACACGACGCGCAGCGATGATTACGAGTCAAATAAACAACCCAATTACGATATGGATTTGAGCGATTTT

AGCATAACTGAGGTTGAAGCCACTCAATATTTAACTCTGTTGCTGACCGTCGAACATGCCTATTTACATT

ATTATATTTTAAAATTACGGGGTGTTTGAATATTGCAAATCGCTAACGGACCATTCGCTTTTTACCAACA

AATTGCGATCGACAATGAGCACAAAAACGTCTAATTTACTGTTAAGCAAATTCAAATTTACCATTGAAGA

TTTTGACAAAATAAACTCAAATTCTGTAACATCAGGGTTTAATATATATAATTTTAATAAATAATTAAAT

AA

>Ac-IE-1 length=371 numreads=11

CAGAATGTAGATAATGTAAAGGGTCACAATTTTATAGTATTGTCTTTCAAAAACGAGGAGCGATTGACTA

TAGCTAAGAAAAACAAAGAGTTTTATTGGATTTCTGGCGAAATTAAAGATGTAGACGTTAGTCAAGTAAT

TCAAAAATATAATAGATTTAAGCATCACATGTTTGTAATCGGTAAAGTGAACCGAAGAGAGAGCACTACA

TTGCACAATAATTTGTTAAAATTGTTAGCTTTAATATTACAGGGTCTGGTTCCGTTGTCCGACGCTATAA

CGTTTGCGGAACAAAAACTAAATTGTAAATATAAAAAATTCGAATTTAATTAATTATACATATATTTTGA

ATTTAATTAATTATACATATA

>Ac-IE-2 length=327 numreads=2

TGTGGATCGTGAACGGTTTCCCGACATCACTGCCCAGCAGTACCAGGATAACATTGCGTCGGAGACAGCT

GCGCAGAGGGCTCTGCAACGAGGTTTAGATCTTGAGGCTCAGCTGATGAATGAGATTGCCCCAAGGTCTC

CACTTATAGTCCATCTTATTCGCCGAATTACGTAATACCACAGTCGCCAGATTGTTTGCCTCGCCGCAGT

CTCCGCAGCCGCAGCAGCAGCAGCAGCAGCAATCAGAACCCGAAGAAGAAGTAGAGGTTTCGTGTAATAT

TTGTTTACTACTTTTAAAGACACTTAACGTAATTCCTCGTTTGTGAC

>Ac-IE-2 length=436 numreads=2

ACTCGTAACCAGGATTCCATCAAACAGCATTGGCGCGAGCTTTTTAGAAAATAACACGGTCAACAATCAC

ACCACGGACTTGAACTATGTGGAGCAATTGCAAAAAGAACTGTCCGAGCTGCGAGCCAAGACCAGCCAAG

TTGAACATAAAATGACCATGTTAAACAGCGACTACATTATGCTTAAACACAAGCATGCTGTCGCCGAATT

AGATTTACAAAAGGCAAACTATGACTTGCAAGAATCTACCAAGAAATCAGAAGAGTTGCAATCGACTGTG

AATAATCTGCAAGAACAATTGCGTAAGCAGGTGGCCGAGTCTCAAGCCAAATTTTCAGAGTTTGAGCGCA

GTAACTCTGATTTAGTTTCTAAGTTACAAACTGTTATGTCTAGACGTTAAGTGTTGTAAATAATATAAAT

AGTATAATATATTAAA

>AcOrf-152 length=386 numreads=2

GGTGAGTGCCTGCTTTTATAGCTGCACGCCTGAGTGGGGGACAGATAACAGAAACTGCAGCCTGTGATAT

GATAAATTTAAAATGAATTTTTTTGTCAATTGCAAAAAAGTTCACTTTTGCCTGACACTCCATATACAGT

ACAATCTCTACAAATCGTAGACTATTTTATTAGAATAGTCTACACTGTACGATACGCTCCCAATATACTA

CTACACTATCAACTTTTTTGCATTACAAAAAAGTTCATTTTTGCCTGGCAAGTTCCCCCACCACTATTGT

CTTATCAGTCGTGCAGTACTGATAAACAGTATAAATACAGCTGCCGTTCTACTCGTAAGCACAGTTCAAG

CCTCACAGCCTAGTGAACAGTATCCTACCAGCCCAG

>Ac-PE38 length=680 numreads=5

GCGACCACGGTTTTGTTCAAATGCGTCATCAATCTGCAAAGCAACGCGATGAATATTCCGCATTCCACTG

TGTGCTGTCCATTGTGCAATACCCAGGTAAAAATGTGGCGTTCCTTAAAGCCTAACGCTGTTGTGACGTG

TAAGTTTTACAAGAAAACTCAAGAAAGAGTTCCGCCCGTGCAGCAGTATAAAAACATTATTAAAGTGCTA

CAAGAACGGAGCGTGATTAGTGTCGAAGACAACGACAATAATTGTGACATAAATATGGAGAATCAGGCAA

AGATAGCTGCTTTGGAAGCTGAATTGGAAGAAGAAAAAATCACAGTGATCAAGTAGCTTCTGAAAACCGA

CAGCTGATAGAAGAAAATACTCGTCTCAATGAACAGATTCAAGAGTTGCAGCATCAGGTGAGGACATTGG

TGCCGCAACGTGGCATTACGGTTAATCAGCAAATTGGCCGTGACGACAGTGCGCCAGCCGAGCTGAACGA

GCGTTTTCGCTCACTTGTCTATTCGACTATTTCAGAGCTGTTTATTGAAAATCGCGTTCATAGTATTCAA

AATTATGTTTATGCCGGAACTTCTGCTGCTAGTTCATGTGATGTAAATGTTACTGTTAATTTTGGGTTTG

AAAATTAATGTGATATGAAATGTATATATAAAAATGATGGAATAAATAAT
