# Supplementary material for: Analysis of Genes Expression of Spodoptera exigua Larvae upon AcMNPV Infection
Source: PLoS One. 2012 Jul 31;7(7):e42462. doi: 10.1371/journal.pone.0042462 (PMC3409162; doi:10.1371/journal.pone.0042462)
Supplement: Table S6 — Primer sequences used in quantitative real-time PCR analysis. (DOC) [file pone.0042462.s006.doc]

Table S6. Primer sequences used in quantitative real-time PCR analysis.

| Amplified target | Contig No. | Primer sequence |
| --- | --- | --- |
| 28S rRNA |  | 5'-CGACGTTGCTTTTTGATCCT-3' |
|  |  | 5'-GCAACGACAAGCCATCAGTA-3' |
| Diapausin | 05891 | 5'-GTACCCGCTGTACCCGTGAG-3' |
|  |  | 5'-GACCACCCTCGTGCTGCTC-3' |
| HMG176A | 00758 | 5'-GTTGAGTTGCCACGGTAGTTGATG-3' |
|  |  | 5'-CCTGATTCTTGTTGCGGTGCTC-3' |
| IFDR | 00274 | 5'-CGAGCGAGACAGTGCTGAGT-3' |
|  |  | 5'-GGACCATGTGTGAGGTTCGT-3' |
| Myofilin | 00148 | 5'-GAAGACATCAGAGCCGAGGAGAG-3' |
|  |  | 5'-GGTGGACGGGCAGGTAACG-3' |
| LIM | 02069 | 5'-CAGGTCCGTCGCAAGCAATG-3' |
|  |  | 5'-CCGTGAATGGCATCGCAAGTG-3' |
| Nimrod B2 | 03515 | 5'-TGACTGCCGCTGTTCTTCTGG-3' |
|  |  | 5'-TAAGTTGGTGGTCTGCCGTAAGG-3' |
| CycP450 | 00461 | 5'-GGACTCTCACAATGACAAGGACAAC-3' |
|  |  | 5'-TGGAGCGTTAATCAACAAGAAGAAGG-3' |
| CG15449 | 04171 | 5'-CTACGATTCCGACGCTCAGACC-3' |
|  |  | 5'-GAACAAGGCAACTCCGATCAACG-3' |
| HMG176B | 03440 | 5'-CGATGAAGAGTCTGATTCTGGTTGC-3' |
|  |  | 5'-GGTCTGATGGCTCCCTGGTAAAG-3' |
| Lipase | 00324 | 5'-GGTCCAAACCTGTGACACGA-3' |
|  |  | 5'-CAGTTCCTCGGCAACTTCCT-3' |
| LCP-1 | 00209 | 5'-CGTGAAGAGGTTGGTGAAGTGAAG-3' |
|  |  | 5'-CTGGCGGGCTTGGGAATGG-3' |
| Profilin | 00304 | 5'-TTCATACAGAGAAATTACGACGGCTTG-3' |
|  |  | 5'-TGGCACAGACCGCATCATACG-3' |
| DEAD helicase | 05985 | 5'-GAGTATTCGGAGGCGGGTATCAC-3' |
|  |  | 5'-CTTTAGCGACAGCGAGTTCTTGG-3' |
| Glutamine synthase | 00359 | 5'-ATGTTACTTGGTGTTGCTCTTGCG-3' |
|  |  | 5'-CTATTATGTTCGTGCCTGGTTGCC-3' |
| Tetraspanin 39D | 04140 | 5'-CGGTGGTGTAGGCATAGGAGTTG-3' |
|  |  | 5'-ACGAATGGAGCGAGCGAGAC-3' |
| Ornithine AT | 00125 | 5'-CGACACCGATGCCCTAGAGAAG-3' |
|  |  | 5'-ACCAGTGCGACCCAAACCG-3' |
| CSP | 04227 | 5'-ACACCACCGCAGCCAAGAC-3' |
|  |  | 5'-GCACCAGTCATCATCAACCGAATC-3' |
| Lysozyme | 05810 | 5'-TTTACATGAACTAGGTCTTTGGTCTG-3' |
|  |  | 5'-CGATGCCTGGTATGGATGGAAG-3' |
| Calreciculin | 01245 | 5'-TCTACGCTCTGTCTCGCAAGTTC-3' |
|  |  | 5'-CCACCTCCGCAGTCAATGTCC-3' |
| Hexamerin 2 | 00429 | 5'-CAGCTGTCGTCGGCCATA-3' |
|  |  | 5'-ACGTGTCCGTTGTTGGTACG-3' |
| GLV1 | 00520 | 5'-CTCTTTATTCGGCCGAGGTG-3' |
|  |  | 5'-TGGCGTTATCATTGGACCAG-3' |
| ATT | 06204 | 5'-AACAAGGTGGGAGCATCTCTG-3' |
|  |  | 5'-AGCCGCTACTCATGAAGGGA-3' |
| JHBP1 | 03571 | 5'-CCATCGATGTCGTGGGTACTT-3' |
|  |  | 5'-CTCCATCAGACCCGTCAACA-3' |
| JHBP2 | 05002 | 5'-GCTGATTCCGGAGAATTGAAG-3' |
|  |  | 5'-CCGTGTATTCACCCACCAAA-3' |
